# Association between short-term exposure to ambient air pollutants and biomarkers indicative of inflammation and oxidative stress: a cross-sectional study using KoGES-HEXA data

**DOI:** 10.1265/ehpm.23-00199

**Published:** 2024-03-16

**Authors:** Ji Hyun Kim, Hae Dong Woo, Jane J Lee, Dae Sub Song, Kyoungho Lee

**Affiliations:** Division of Population Health Research, Department of Precision Medicine, Korea National Institute of Health, Korea Disease Control and Prevention Agency, 200 Osongsaengmyeong2-ro, Osong-eup, Heungdeok-gu, Cheongju-si 28160, Chungcheongbuk-do, Republic of Korea

**Keywords:** Air pollutants, Biomarkers, Inflammation, Oxidative stress, Middle-aged adults, Korea

## Abstract

**Background:**

Air pollution-induced systemic inflammation and oxidative stress are hypothesized to be the major biological mechanisms underlying pathological outcomes. We examined the association between short-term exposure to ambient air pollutants and biomarkers of inflammation and oxidative stress in 2199 general middle-aged Korean population residing in metropolitan areas.

**Methods:**

Serum levels of inflammatory cytokines (interleukin [IL]-1β, IL-6, IL-8, IL-10, and tumor necrosis factor [TNF]-α) and urinary levels of 8-hydroxy-2′-deoxyguanosine (8-OHdG) were measured. Daily concentrations of a series of air pollutants (particulate matter [PM]_10_, PM_2.5_, SO_2_, NO_2_, CO, and O_3_) were predicted using the Community Multiscale Air Quality modeling system, and participant-level pollutant exposure was determined using geocoded residential addresses. Short-term exposure was defined as the 1- to 7-day moving averages.

**Results:**

The multivariable-adjusted linear models controlling for the sociodemographic, lifestyle, temporal, and meteorological factors identified positive associations of PM with IL-1β, IL-8, IL-10, TNF-α, and 8-OHdG levels; SO_2_ with IL-10 levels, CO with IL-1β, IL-10, and TNF-α levels; and O_3_ with IL-1β, IL-8, and 8-OHdG levels. O_3_ levels were inversely associated with IL-10 levels. For each pollutant, the strongest associations were observed for the 7-day average PM and CO with IL-1β (per 10-µg/m^3^ increase in PM_10_: 2.7%, 95% confidence interval [CI] = 0.6–4.8; per 10-µg/m^3^ increase in PM_2.5_: 6.4%, 95% CI = 2.4–10.5; per 0.1-ppm increase in CO: 3.3%, 95% CI = 0.3–6.5); the 2-day average SO_2_ with IL-10 levels (per 1-ppb increase in SO_2_: 1.1%, 95% CI = 0.1–2.1); and the 7-day average O_3_ with IL-8 levels (per 1-ppb increase in O_3_: 1.3%, 95% CI = 0.7–1.9).

**Conclusions:**

Short-term exposure to ambient air pollutants may induce oxidative damage and pro-inflammatory roles, together with counter-regulatory anti-inflammatory response.

**Supplementary information:**

The online version contains supplementary material available at https://doi.org/10.1265/ehpm.23-00199.

## 1. Background

Air pollution is defined as a contaminated ambient atmosphere caused by the presence of a heterogeneous combination of particulate and gaseous constituents that vary according to the prevailing sources and physicochemical composition [1, 2]. The global burden of disease and death attributable to ambient air pollutants is substantial [3, 4]. Exposure to air pollutants contributes to morbidity and mortality through an increased onset of chronic diseases, which are probably mediated by pathophysiological processes involving oxidative stress and inflammation [5–7].

To date, multiple sensitive and critical biomarkers reflecting inflammation and oxidative stress have been used to assess the short-term health effects of air pollutants, including circulating pro-inflammatory cytokines and urinary markers of oxidative DNA damage [7–11]. Stimulation of the interplay between pro-inflammatory cytokines (e.g., tumor necrosis factor [TNF]-α, interleukin [IL]-1, IL-6, and IL-8) as a cascade of reactions can upregulate acute-phase responses (e.g., C-reactive protein [CRP], fibrinogen, and serum amyloid A levels) [12–14]. In contrast, IL-10 suppresses the capacity of monocytes and macrophages to present antigens to T cells, downregulating the expression of pro-inflammatory cytokines [14–16]. The delicate balance between pro- and anti-inflammatory cytokines controls the prevalence of normal and pathological conditions [17]. 8-hydroxy-2′-deoxyguanosine (8-OHdG) is a marker of oxidatively modified guanine derivatives, which are directly proportional to oxidative stress levels and represent the balance between oxidative DNA damage and repair across all cells in an organism [18]. Urine is the most preferred biospecimen for measuring 8-OHdG in epidemiological studies because of its noninvasive and highly stable properties [18].

Despite previous attempts to establish associations between ambient pollutants and these biomarkers, the results remain inconclusive. The effect sizes in these studies varied largely in relation to the research methodologies, the types of air pollutants evaluated, and the geographical regions where these studies were conducted [9, 11]. The existing studies mostly focused on associations with particulate matter (PM: PM_10_ and PM_2.5_), wherein pooled estimates of TNF-α or IL-6 levels showed significant and positive associations with PM_2.5_ levels, and supporting evidence on gas-phase pollutants and other cytokines was scarce, especially in Asian countries [8–10]. Studies investigating the effect of short-term PM exposure on oxidative stress levels have reported conflicting findings, although many studies showed induced associations [11], and studies focusing on gaseous pollutants and oxidative stress are scarce. Therefore, a study incorporating a series of air pollutants and a panel of cytokines and oxidative damage is required to investigate the early biological health effects of air pollutants.

Herein, we selected a series of circulating cytokines (pro-inflammatory: IL-1β, IL-6, IL-8, and TNF-α; and anti-inflammatory: IL-10) and urinary 8-OHdG as biomarkers of inflammation and oxidative stress, respectively. We examined the association between short-term exposure to ambient air pollutants and the concentrations of these markers in a general non-current smoking midlife Korean population residing in metropolitan areas.

## 2. Methods

### 2.1. Study population

**Fig. 1:**
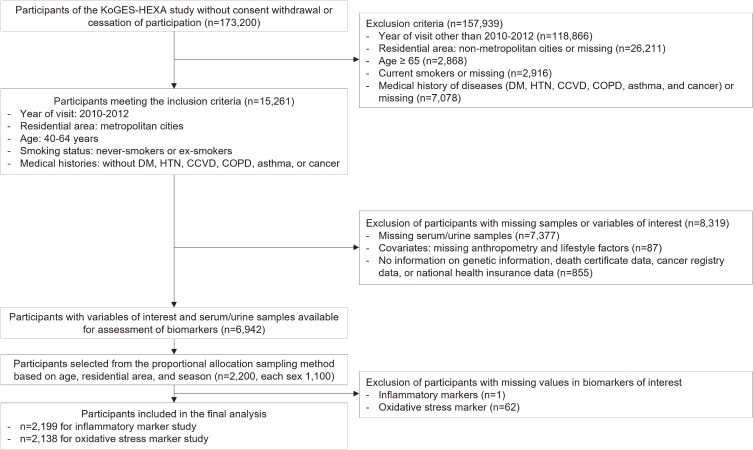
Flowchart of the study participants. KoGES-HEXA: Korean Genome and Epidemiology Study-Health Examinee; DM: diabetes; HTN: hypertension; CCVD: cardia- and cerebrovascular diseases; COPD: chronic obstructive pulmonary diseases.

This cross-sectional analysis used baseline data from the Health Examinee (HEXA), a nationwide population-based cohort of the Korean Genome and Epidemiology Study (KoGES). Information on the background, design, and methodology of the survey has been described elsewhere [19]. In brief, participants enrolled in the KoGES-HEXA were aged ≥40 years at baseline and visited health examination centers and hospitals located in the metropolitan areas and major cities. They underwent anthropometric measurements and clinical investigations and answered self-administered questionnaires assessing sociodemographic characteristics, health-related lifestyle factors, and medical histories. This research is a sub-study originated from the KoGES-HEXA cohort, utilizing the biospecimen collected in 2010–2012 to focus on investigating the biomarkers of inflammation and oxidative stress (Fig. 1). The current study evaluated a subset of 2199 participants (1099 men and 1100 women) meeting the following criteria: (1) middle-aged (aged 40–64 years) at baseline; (2) enrolled in 2010–2012; (3) residing in the metropolitan cities (Seoul, Incheon, Busan, Daegu, Gwangju, and Ulsan); (4) non-current smokers; (5) without medical history of diseases (diabetes, hypertension, cardio- and cerebrovascular diseases, chronic obstructive pulmonary disease, asthma, and cancer); and (6) without missing values for any of the biomarkers of interest. Written informed consent was obtained from all participants prior to the examinations, and the study protocol was approved by the appropriate Institutional Review Boards.

### 2.2. Ambient air pollution and meteorological factors

Data for ambient air pollutant concentrations and meteorological factors were estimated using the Community Multiscale Air Quality (CMAQ) model, which were assimilated with surface and aerosol data; extensive explanations for this process have been reported elsewhere [20–22]. The data for meteorological factors, including daily average temperature (°C) and humidity (%), and gaseous material (sulfur dioxide [SO_2_], nitrogen dioxide [NO_2_], carbon monoxide [CO], and ozone [O_3_]) were provided in every 9-km grid resolution. For PM, multiple linear regression with aerosol optical depth (AOD) data from the National Aeronautics and Space Administration Terra and Aqua satellites was further applied to enhance the accuracy of CMAQ estimates assimilated with surface data, resulting in a 1-km grid unit. Since AOD indicates the extent to which solar radiation is attenuated by aerosols in the atmosphere, this data was specifically applicable to PM but not to gaseous substances. On the daily scale, the root mean square errors of estimated air pollutant levels compared to the measured levels were 15.16 µg/m^3^ for PM_10_, 8.31 µg/m^3^ for PM_2.5_, 3.0 ppb for SO_2_, 6.9 ppb for NO_2_, 0.16 ppm for CO, and 7.2 ppb for O_3_ [21]. To estimate the individual exposure of each participant, the KoGES database was merged with a database of air pollution and meteorological factors based on the participants’ dates of examination and their geocoded grid units using ArcGIS (ESRI, Inc., Redland, CA, USA). We defined short-term exposure to ambient air pollutants as the 1-, 2-, 3-, 5-, and 7-day moving averages (calculated from lag 1 to lag 7) based on previous research [23, 24].

### 2.3. Laboratory assays for biomarker measurement

Participants’ venous blood and first morning spot urine samples for the clinical chemistry tests were obtained after a minimum of 8 hr overnight fasting. Serum aliquots were centrifuged; and both serum and urine samples were stored at −80 °C before being assayed. Serum levels of inflammation-related cytokines (pro-inflammatory markers: IL-1β, IL-6, IL-8, and TNF-α; and an anti-inflammatory marker: IL-10) and a urinary oxidative stress marker (8-OHdG) were quantified by commercially available enzyme-linked immunosorbent assay kits according to the manufacturers’ protocols. The inter- and intra-assay coefficients of variation (CVs) were, respectively, 7.88 and 3.56 for IL-1β, 6.45 and 3.94 for IL-6, 9.12 and 6.99 for IL-8, 7.27 and 4.55 for IL-10, and 8.97 and 4.46 for TNF-α; and the minimum detection limits for the corresponding biomarkers were, respectively, 0.08, 0.14, 0.04, 0.21, and 0.29 pg/mL. The inter- and intra-assay CV values of urine 8-OHdG levels were 16.42 and 2.27, respectively, and the detection limit was 0.01 ng/mL. Urine creatinine concentrations were measured using Jaffe and enzymatic assays (Roche Diagnostics, Cobas c702 module). Urinary 8-OHdG levels were expressed as a ratio of creatinine levels to correct for the effect of urine dilution.

### 2.4. Covariates

The following individual-level covariates were selected a priori and included in the main model after a variance inflation test with a cut-off value of 5 to minimize the presence of multicollinearity: date of examination as a linear term [23, 24], continuous variables of age (years), body mass index (kg/m^2^), and the corresponding moving averages of major meteorological factors (temperature [°C] and relative humidity [%]); and categorical variables of season (spring, summer, fall, and winter), weekday of examination, residential region, sex, smoking history (non or former), alcohol consumption status (non, former, or current), regular exercise (yes or no), education attainment (less than middle school [9 years], middle to high school [9–12 years], or college or more [>12 years]), occupation (professional, administrative; office, sales, and service; laborer, agricultural; others, unemployed; or missing), and marital status (married or cohabiting; and single/divorced/widowed/separation, or others).

### 2.5. Statistical analyses

Summary statistics of the participants’ characteristics and biochemical parameters are described as mean (standard deviation) or number (percentage). The distributions of ambient air pollution exposure and meteorological factors were summarized according to the 7-day average exposure, and the corresponding correlation coefficients were obtained. To investigate the associations between short-term exposure to ambient air pollutants and the levels of biomarkers, first, values were natural log-transformed (ln) before the analysis to achieve approximate normality; second, the estimated regression coefficients (β) from the crude and multivariable-adjusted linear regression models were obtained; and finally, the effects were exponentiated back to the original units of the biomarker and reported as percentage changes of each biomarker per unit increase in air pollutants (PM: 10 µg/m^3^; SO_2_, NO_2_, and O_3_: 1 ppb; and CO: 0.1 ppm). To evaluate the robustness of our findings, we performed sensitivity analyses on the association between air pollutants and biomarkers (1) by excluding extreme outlying values of biomarkers, which may arise from measurement errors and can be located at the lower (<0.5th percentile) or higher (>99.5th percentile) ends of the distribution; (2) by including only non-smokers; and (3) by mutually adjusting for co-pollutants using a two-pollutant model. Of note, we applied the restriction of correlation of |0.70| or higher for the combination of pollutants included in the two-pollutant model. All statistical analyses were performed using SAS version 9.4 (SAS Institute, Cary NC, USA), and two-sided *P*-values less than 0.05 were considered statistically significant. Figures were visualized using R version 4.0.2 (R Foundation).

## 3. Results

### 3.1. Study population

Table 1 presents the characteristics of the participants (mean age, 50.6 ± 6.9 years). Participants were more likely to be non-obese (69.5%), non-smokers (69.4%), current drinkers (54.2%), regularly engaged in physical activity (58.5%), educated for more than 9 years (90.5%), married (90.4%), and recruited in this study during the summer or fall seasons (70.0%). In terms of the levels of six analytes (inflammatory and oxidative stress biomarkers), the median values were 0.98 pg/mL for IL-1β, 1.53 pg/mL for IL-6, 14.97 pg/mL for IL-8, 0.89 pg/mL for IL-10, 12.09 pg/mL for TNF-α, and 4.34 µg/g creatinine for 8-OHdG, respectively.

**Table 1:** General characteristics of the study participants

**Characteristics**	**Mean ± SD or No. (%)**		

	**Total (n = 2199)**	**Men (n = 1099)**	**Women (n = 1100)**
**Age (years)**	50.63 ± 6.94	51.49 ± 7.03	49.78 ± 6.74
**Sex**			
Males	1099 (50.0)	-	-
Females	1100 (50.0)	-	-
**Body mass index (kg/m^2^)**	23.67 ± 2.79	24.16 ± 2.62	23.17 ± 2.86
**Body mass index in categories**			
<25	1529 (69.5)	411 (37.4)	259 (23.6)
≥25	670 (30.5)	688 (62.6)	841 (76.5)
**Smoking history**			
None	1527 (69.4)	450 (41.0)	1077 (97.9)
Ex-smoker	672 (30.6)	649 (59.1)	23 (2.1)
**Alcohol consumption**			
None	935 (42.5)	237 (21.6)	698 (63.5)
Ex-drinker	72 (3.3)	61 (5.6)	11 (1.0)
Current drinker	1192 (54.2)	801 (72.9)	391 (35.6)
**Regular exercise, yes**			
Yes	1287 (58.5)	385 (35.0)	527 (47.9)
No	912 (41.5)	714 (65.0)	573 (52.1)
**Occupation**			
Professional, administrative	441 (20.1)	303 (27.6)	138 (12.6)
Office, sales, and service	737 (33.5)	449 (40.9)	288 (26.2)
Laborer, agricultural	335 (15.2)	241 (21.9)	94 (8.6)
Others, unemployed	683 (31.1)	103 (9.4)	580 (52.7)
Missing	3 (0.1)	3 (0.3)	0 (0.0)
**Education**			
Less than middle school (<9 years)	209 (9.5)	73 (6.6)	136 (12.4)
High school (9–12 years)	1018 (46.3)	413 (37.6)	605 (55.0)
College or more (>12 years)	972 (44.2)	613 (55.8)	359 (32.6)
**Marital status**			
Married	1988 (90.4)	1026 (93.4)	962 (87.5)
Single, divorced, widowed, or separation	198 (9.0)	70 (6.4)	128 (11.6)
Missing	13 (0.6)	3 (0.3)	10 (0.9)
**Year of enrollment**			
2010	1244 (56.6)	649 (59.1)	595 (54.1)
2011	793 (36.1)	389 (35.4)	404 (36.7)
2012	162 (7.4)	61 (5.6)	101 (9.2)
**Season of examination**			
Spring (Mar–May)	415 (18.9)	195 (17.7)	220 (20.0)
Summer (Jun–Aug)	771 (35.1)	382 (34.8)	389 (35.4)
Fall (Sep–Nov)	768 (34.9)	394 (35.9)	374 (34.0)
Winter (Dec–Feb)	245 (11.1)	128 (11.7)	117 (10.6)
**Circulating biomarkers**			
IL-1β (pg/mL)	1.32 ± 1.49	1.33 ± 1.57	1.32 ± 1.41
(Median, IQR)	(0.98, 0.91)	(0.95, 0.87)	(1.02, 0.94)
IL-6 (pg/mL)	2.05 ± 4.94	1.90 ± 2.98	2.20 ± 6.31
(Median, IQR)	(1.53, 1.24)	(1.49, 1.22)	(1.57, 1.28)
IL-8 (pg/mL)	23.21 ± 35.34	24.79 ± 30.12	21.63 ± 39.82
(Median, IQR)	(14.97, 13.60)	(16.02, 15.56)	(14.02, 11.70)
IL-10 (pg/mL)	1.67 ± 24.52	2.17 ± 34.65	1.17 ± 1.49
(Median, IQR)	(0.89, 0.84)	(0.85, 0.79)	(0.92, 0.86)
TNF-α (pg/mL)	12.66 ± 4.59	13.18 ± 4.86	12.14 ± 4.23
(Median, IQR)	(12.09, 4.31)	(12.55, 4.49)	(11.74, 4.20)
**Urinary biomarker** ^a^			
8-OHdG (µg/g creatinine)	5.11 ± 3.44	4.48 ± 2.85	5.74 ± 3.84
(Median, IQR)	(4.34, 4.03)	(3.87, 3.43)	(4.99, 4.27)

Values are presented as mean ± standard deviation for continuous variables and n (%) for categorical variables; IL: interleukin; TNF: tumor necrosis factor; 8-OHdG: 8-hydroxy-2′-deoxyguanosine; IQR: interquartile range. ^a^2138 participants (1075 men and 1063 women).

### 3.2. Levels of ambient air pollutants

Summary statistics of ambient air pollutants and meteorological factors are summarized in Table 2. The 7-day average exposure values for PM_10_, PM_2.5_, SO_2_, NO_2_, CO, and O_3_ were 47.79 µg/m^3^, 24.35 µg/m^3^, 5.53 ppb, 27.23 ppb, 0.45 ppm, and 22.75 ppb, respectively. Correlations between pollutant levels ranged from −0.59 to 0.92. The strongest positive correlation was observed between PM_10_ and PM_2.5_ (r = 0.92), followed by NO_2_ and CO levels (r = 0.76), and PM with gaseous pollutant levels (NO_2_ and CO). O_3_ level was inversely correlated with the levels of other gas-phase pollutants (NO_2_ and CO). Among meteorological factors, temperature and relative humidity were positively correlated (r = 0.53), and they showed inverse correlations with the pollutant levels, except for O_3_ and temperature.

**Table 2:** Summary statistics and correlation coefficients of the ambient air pollutants and meteorological factors (7-day average)

**Pollutant (unit)**	**Mean ± SD**	**Max**	**Q3**	**Median**	**Q1**	**Min**	**IQR**	**Pearson’s correlation coefficient**							

								**PM_10_**	**PM_2.5_**	**SO_2_**	**NO_2_**	**CO**	**O_3_**	**Temp**	**RH**
PM_10_ (µg/m^3^)	47.79 ± 15.92	151.55	56.20	46.21	36.35	18.12	19.85	1.00	0.92*	0.33*	0.62*	0.61*	0.07*	−0.42*	−0.38*
PM_2.5_ (µg/m^3^)	24.35 ± 7.94	52.11	29.10	23.31	18.47	8.67	10.63		1.00	0.33*	0.61*	0.63*	0.11*	−0.34*	−0.30*
SO_2_ (ppb)	5.53 ± 2.93	27.19	6.41	4.86	3.72	1.31	2.68			1.00	0.29*	0.11*	0.02	−0.09*	−0.14*
NO_2_ (ppb)	27.23 ± 9.53	57.15	34.35	26.87	19.57	6.52	14.78				1.00	0.76*	−0.29*	−0.39*	−0.35*
CO (0.1 ppm)	4.49 ± 1.38	10.33	5.24	4.33	3.55	1.84	1.69					1.00	−0.39*	−0.59*	−0.25*
O_3_ (ppb)	22.75 ± 8.10	49.14	29.38	21.61	16.20	6.69	13.17						1.00	0.33*	−0.16*
Temp (°C)	16.33 ± 8.80	30.50	24.05	17.31	10.50	−9.34	13.55							1.00	0.53*
RH (%)	70.22 ± 9.85	96.56	77.44	70.84	62.95	39.67	14.49								1.00

SD: standard deviation; Max: maximum; Min: minimum; Q1, Q3: first and third quartile, respectively; IQR: interquartile range; PM_10_, PM_2.5_: particulate matter with aerodynamic diameter <10 µm and <2.5 µm, respectively; SO_2_: sulfur dioxide; NO_2_: nitrogen dioxide; CO: carbon monoxide; O_3_: ozone; Temp: temperature; RH: relative humidity; *P < 0.001.

### 3.3. Association between short-term exposure to air pollutants and biomarker levels

**Fig. 2:**
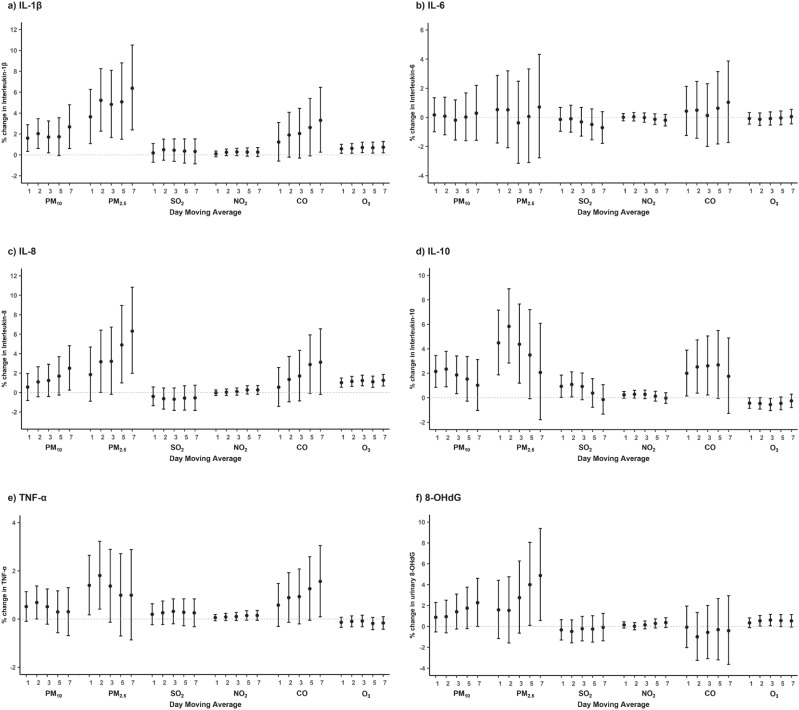
Associations between short-term exposure to air pollutants and biomarkers of inflammation and oxidative stress. The models are adjusted for date of examination as a linear term, continuous variables of age, body mass index, and the corresponding moving averages of temperature and relative humidity; and categorical variables of season, weekday of examination, residential region, sex, smoking history, alcohol consumption status, regular exercise, education attainment, occupation, and marital status. Estimates are presented as percentage changes with 95% confidence intervals in each biomarker level per 1-unit increase in 1- to 7-day average ambient air pollution exposure (units for PM: 10 µg/m^3^; SO_2_, NO_2_, and O_3_: 1 ppb; and CO: 0.1 ppm). PM_10_, PM_2.5_: particulate matter with aerodynamic diameter <10 µm and <2.5 µm, respectively; SO_2_: sulfur dioxide; NO_2_: nitrogen dioxide; CO: carbon monoxide; O_3_: ozone; IL: interleukin; TNF: tumor necrosis factor; 8-OHdG: 8-hydroxy-2′-deoxyguanosine.

The correlations between short-term exposure to air pollutants and biomarker levels are shown in Table 3. PM, NO_2_, and CO levels were significantly and positively correlated with cytokine and 8-OHdG levels, whereas SO_2_ and O_3_ levels were negatively correlated with cytokine levels. The associations between short-term exposure to air pollutants and circulating biomarkers of inflammation and oxidative stress are shown in Fig. 2 and Supplementary Table 1. Several pollutants demonstrated positive associations with some biomarkers after controlling for potential confounders. For each one-unit increase in the short-term concentrations of air pollutants, the circulating IL-1β levels increased, with a larger magnitude of associations at longer duration of exposure (per 10-µg/m^3^ increase in PM_10_: 2.69% [95% confidence interval (CI): 0.62, 4.80] and PM_2.5_: 6.38% [2.40, 10.52]; per 0.1-ppm increase in CO: 3.31% [0.26, 6.46]; and per 1-ppb increase in O_3_: 0.74% [0.20, 1.28]). IL-8 levels were also positively associated with air pollutants, with the strength of the association being greater at longer exposure durations (PM_10_: 2.51% [0.26, 4.81], PM_2.5_: 6.32% [1.99, 10.84], and O_3_: 1.27% [0.68, 1.86]). The levels of IL-10 were associated with air pollutants, with the largest effect sizes ranging between 2- to 3-day average exposure (PM_10_: 2.34% [0.91, 3.80] and PM_2.5_: 5.83% [2.84, 8.91]; per 1-ppb increase in SO_2_: 1.09% [0.07, 2.12]; CO: 2.61% [0.23, 5.05]; and O_3_: −0.55% [−1.05, −0.05]). The TNF-α level was elevated by 2-day average exposure to PM (PM_10_: 0.69% [0.01, 1.38] and PM_2.5_: 1.81% [0.41, 3.22]) and 7-day average exposure to CO (1.56% [0.10, 3.05]). Urinary 8-OHdG levels were elevated by a 7-day average exposure to PM_2,5_ (4.88% [0.56, 9.38]) and a 3-day average exposure to O_3_ (0.62% [0.07, 1.16]).

**Table 3:** Pearson’s correlation coefficients of the ambient air pollutants (7-day average) and the levels of biomarkers

**Pollutant (unit)**	**IL-1β**	**IL-6**	**IL-8**	**IL-10**	**TNF-α**	**8-OHdG**
PM_10_ (µg/m^3^)	0.10***	0.05*	0.06**	0.05*	0.05*	0.13***
PM_2.5_ (µg/m^3^)	0.10***	0.04	0.07**	0.05*	0.05*	0.11***
SO_2_ (ppb)	−0.01	−0.05*	−0.08***	0.02	−0.03	0.04
NO_2_ (ppb)	0.11***	0.04	0.08***	0.05*	0.11***	0.09***
CO (0.1 ppm)	0.10***	0.08***	0.08***	0.06**	0.12***	0.08***
O_3_ (ppb)	−0.01	−0.01	0.03	−0.03	−0.09***	0.04*

PM_10_, PM_2.5_: particulate matter with aerodynamic diameter <10 µm and <2.5 µm, respectively; SO_2_: sulfur dioxide; NO_2_: nitrogen dioxide; CO: carbon monoxide; O_3_: ozone; IL: interleukin; TNF: tumor necrosis factor; 8-OHdG: 8-hydroxy-2′-deoxyguanosine. The levels of air pollutants and biomarkers were natural log-transformed before the correlation analysis. *P < 0.05; **P < 0.01; ***P < 0.001.

### 3.4. Sensitivity analysis

Sensitivity analyses excluding outliers (<0.5th or >99.5th percentile) did not substantially influence the distribution of biomarkers or the associations between short-term air pollution exposure and the biomarkers of interest; and including only non-smokers slightly elevated the strength of associations (Supplementary Figs. 1–6 and Tables 2–4). In the two-pollutant models simultaneously adjusting for each other, the size and direction of effect estimates generally remained consistent except for a slightly attenuated or elevated changes compared to the main model. However, we observed a trend of association in terms of a pollutant combination of PM and CO with 8-OHdG levels: the association between PM_2.5_ with 8-OHdG were increased to a greater extent when controlling for CO. In contrast, CO, which showed null association with 8-OHdG in the main model, exhibited a reduced association when controlling for PM (Supplementary Figs. 1–6 and Table 5).

## 4. Discussion

This study investigated the association between short-term exposure to air pollutants and biomarkers of inflammation and oxidative stress among participants who were non-current smokers. The findings showed positive associations of PM with IL-1β, IL-8, IL-10, TNF-α, and 8-OHdG levels; SO_2_ with IL-10 levels; CO with IL-1β, IL-10, and TNF-α levels; and O_3_ with IL-1β, IL-8, and 8-OHdG levels. An inverse association was observed between O_3_ and IL-10 levels.

The current study showed positive associations between short-term PM exposure and the levels of several pro-inflammatory cytokines. Prior meta-analyses reported that PM elevates the circulating levels of acute-phase proteins, which are upregulated by a complex signaling cascade, along with their key mediators (e.g., IL-6 and TNF-α) [8, 10, 25]. For gaseous pollutants, a limited number of observational studies have investigated the associations with short-term exposure and the levels of cytokines; induced associations have been observed with TNF-α and CRP, but the literature to draw robust conclusions regarding associations with other biomarkers is limited [9]. Several plausible lines of evidence have linked the association between short-term exposure to air pollutants, particularly PM, and higher elicitation of cytokines. Pollutants can be involved in airway inflammation and oxidative stress, or particles can be directly translocated into the bloodstream, both of which result in systemic inflammation [26]. IL-1β is a potent inflammatory mediator whose production relies on the activation of NLRP3 inflammasome complex, which can be promoted by exogenously applied pollutants [27, 28]. It can induce the levels of other cytokines from many cells, stimulate hematopoiesis, and activate endothelial cells, leading to acute-phase responses [29]. An animal study using lung macrophages from O_3_-exposed mice showed higher mitochondrial reactive oxygen species (ROS) levels and increased caspase-1 activation, thereby increasing the production of IL-1β [30]. IL-8 is an important chemokine produced under inflammatory stimulation (e.g., diesel exhaust particles, which are a major constituent of air pollutants in urban regions) and attracts and activates neutrophilic granulocytes [31]. TNF-α also plays a crucial role in the processes underlying innate or adaptive immune responses and insulin resistance and can be affected by short-term exposure to air pollutants [8, 9, 32].

Short-term air pollutant exposure showed null associations with IL-6, which is induced with other alarm cytokines, namely, TNF and IL-1, which are involved in the elicitation of acute-phase proteins [14, 33]. IL-6 is a multifunctional cytokine; in addition to the pro-inflammatory regulation of hepatocytes to synthesize acute-phase proteins, it stimulates both innate and adaptive immunity (humoral B cell and cellular T cell responses) by activating T-helper 17 cells and inhibiting regulatory T cells [29, 34]. Several factors could explain this observation. First, the physiological condition may have affected the results. In agreement with our study, a subgroup meta-analysis of a general population showed smaller effect sizes for the associations between PM_2.5_ and IL-6 levels, in comparison with the diseased population (for each 10-µg/m^3^ increase in short-term exposure to PM_2.5_, IL-6 levels increased by 20.7% and 3.5% in the diseased and general populations, respectively) [10]. Second, air pollutants may induce immediate responses in IL-6 levels. Interestingly, a repeated-measures study of myocardial infarction survivors demonstrated an immediate response of IL-6 levels to ambient air pollution; the levels were slightly increased at 6–11 hr after exposure, followed by a clear increase at 12–17 hr after exposure, and dropped back to null association thereafter [35]. Another study of healthy middle-aged adults found inconsistent associations between 2 hr air pollutant exposure or inhaled doses with changes in multiple inflammatory markers (IL-6, IL-8, IL-10, TNF-α, and CRP), as well as a marker of lung epithelial damage (Clara cell protein 16) measured before and 6 hr after the exposure [36]. Our findings are also supported by the results of several experimental studies that showed null findings for IL-6 when stimulated with pollutants for less than 15 min or more than 24 hr [37, 38]. Taken together, the circulating levels of IL-6 exhibited fluctuations depending on the duration of air pollutant exposure, demonstrating a time-dependent and time-sensitive relationship. This may have resulted in absence of detectable associations for IL-6 in the present study, which needs to be identified in the future epidemiological studies. However, we should note that the inconsistencies across previous studies may have attributed to differences in the study designs, participants’ demographic characteristics or genetic backgrounds, exposure assessment methods, or residual confounding. Moreover, the composition of air pollutants was heterogeneous across the study regions, forming a complex mixture of materials [39, 40].

In contrast to pro-inflammatory cytokines, IL-10 is an immunoregulatory cytokine known to inhibit the capacity of monocytes and macrophages to present antigens to T cells and further downregulate the expression of pro-inflammatory cytokines [15]. In the current study, PM, SO_2_, and CO levels were associated with elevated IL-10 levels. Although the mechanism by which inhaled air pollutants affect cytokine expression has not been fully characterized, enhanced anti-inflammatory cytokine expression can be partially explained as a counter-regulatory response to arrest and limit inflammatory cascades in the ongoing inflammation within the tissue [41]. However, the reason for the inverse association between O_3_ and IL-10 levels remains unclear. Similar findings were reported in experimental studies. When the effect of short-term ozonation on isolated peripheral blood mononuclear cells from nine donors was measured, a progressive reduction in IL-10 levels was observed during the 48- and 72-hr incubation with O_3_ concentrations greater than or equal to 20 µg/mL, while no differences were observed in IL-6 production between cells exposed or unexposed to O_3_ [38]. An in vivo study suggested that other inflammatory mediators or injuries may not be sufficiently regulated by IL-10, which may further contribute to the pathophysiology of O_3_-induced inflammation [42].

In addition to these inflammatory responses, our study suggests that oxidative damage occurs during episodes of short-term exposure to air pollution (PM_2.5_, O_3_). 8-OHdG is a specific DNA repair product that may reflect oxidative stress as well as a reduced capacity for DNA repair due to the damage caused by PM inhalation [43]. According to a meta-analysis of 13 studies with cohort or panel designs, the pooled percent change of 8-OHdG per 10 µg/m^3^ increase in short-term exposure to PM_2.5_ was null, probably due to large variations in the effect size by research methods and the types of pollutants [11]. Studies investigating the effects of gaseous pollutants on 8-OHdG levels are scarce. Among gas-phase pollutants, O_3_ is a powerful oxidizing agent generated in the troposphere through photochemical reactions involving oxides of nitrogen and hydrocarbons [44]. Upon inhalation, O_3_ undergoes initial reactions with lipid substrates present on the airway surfaces, potentially giving rise to reactive chemicals (e.g., ROS); the mediator cytokines released from the airway cells may enter into the circulation, activate blood elements, and augment the production of acute-phase reactants [45, 46]. Given the important role of O_3_ in these reactions, a repeated-measures study involving 61 healthy college students in China reported that short-term exposure to O_3_ was associated with elevated levels of serum 8-OHdG [45]. Another longitudinal study of 89 healthy office workers in China reported that a 10-ppb increase in 24 hr O_3_ exposure was associated with a significant 13.5% increase in urinary 8-OHdG [47].

The strengths of this study are three-folds. First, the study participants showed residential variability within metropolitan areas, ensuring robust statistical estimates of the effect of ambient air pollution on the levels of biomarkers. Second, a wide range of individual-level covariates (e.g., socioeconomic status and lifestyle factors) were obtained from detailed questionnaires. Third, ambient air pollutant modeling using the CMAQ method assimilated with observations enabled us to obtain high-resolution estimates of air pollutants. However, the following limitations also require consideration. First of all, selection bias might present due to selection of a subgroup from the entire cohort. We included participants residing only in the metropolitan areas. It should be noted that comparing the associations of air pollutant exposure on inflammation and oxidative stress among residents in metropolitan and non-metropolitan areas is an important point to consider; thus, further studies are suggested to explore the association in various residential areas. Next, the associations observed in this study may not be applied to other individuals living in the areas with higher or lower air pollutant levels than the present study. Specifically, when the annual average air pollutant concentration is compared across the capital cities of East Asian countries, China (Beijing) had the highest PM_2.5_ and PM_10_ levels followed by Korea (Seoul) and Japan (Tokyo); Among gaseous pollutants (SO_2_ and NO_2_), Korea had the highest levels followed by China and Japan [48, 49]. Despite declining trends of air pollutants observed in all three countries, the annual levels still exceeded the World Health Organization annual air quality guideline (PM_10_ < 15 µg/m^3^; PM_2.5_ < 5 µg/m^3^, and NO_2_ < 10 µg/m^3^), which may contribute to adverse health outcomes [50]. The future study incorporating a wide range of air pollutant exposure levels are warranted to fully understand the degree of deleterious health effects of airborne environmental toxicants. Although each air pollutant is regulated separately through national and global emission limits or air quality standards, the overall health effects are driven by a mixture of these pollutants. They can interact with other aspects of the environment, ultimately leading to adverse health effects [40]. Moreover, due to the dynamic fluidity of inflammation-driven circulation of cytokines, the underlying processes may not be fully captured by collecting blood at a single time point [41]. Therefore, the cross-sectional nature of this study precluded us from identifying the causal effects. Longitudinal designs with multiple measurements have been suggested to minimize bias and accurately investigate associations. In addition, although we observed acute effects of air pollutants on cytokine expression and oxidative DNA damage, we did not account for genetic factors that may interact with cytokine expression [45, 51]. Additional analyses incorporating genetic information to investigate potential gene-environment interactions are suggested (i.e., genetic polymorphisms involved in the process of detoxification enzymes or cytokine receptors). Furthermore, the estimated concentrations of ambient air pollutants were based on the participants’ residential addresses. Because individuals’ indoor and outdoor living is a continuum and cannot be dichotomized, further confirmation of the health effects of continuous composites through a multidisciplinary effort is warranted [52]. Finally, residual confounding factors may have been present. For instance, medication-related factors may affect the oxidative or inflammatory responses [34], and information on proximity to roadways could serve as a major covariate [53]. However, we could not control for these factors in our analysis and determine whether the results remained robust.

## 5. Conclusions

In conclusion, our findings support the evidence that the systemic inflammatory state and oxidative stress can be activated by short-term exposure to series of air pollutants. These responses may be linked to the induction of ROS and pro-inflammatory roles, together with an elevation of counter-regulatory anti-inflammatory response. Further studies with repeated measurements of biomarkers, relevant genetic variants, and a comprehensive list of covariates are warranted to clarify deleterious health outcomes associated with air pollution in the general population.

## Abbreviations

TNF: tumor necrosis factor
IL: interleukin
CRP: C-reactive protein
8-OHdG: 8-hydroxy-2′-deoxyguanosine
PM: particulate matter
HEXA: health examinee
KoGES: Korean Genome and Epidemiology Study
CMAQ: Community Multiscale Air Quality
AOD: aerosol optical depth
SO_2_: sulfur dioxide
NO_2_: nitrogen dioxide
CO: carbon monoxide
O_3_: ozone
CV: coefficients of variation
ROS: reactive oxygen species

## Supplementary information

**Additional file 1: Supplementary Figure 1.** Summary of main and sensitivity analyses on the associations between short-term exposure to air pollutants and IL-1β. **Supplementary Figure 2.** Summary of main and sensitivity analyses on the associations between short-term exposure to air pollutants and IL-6. **Supplementary Figure 3.** Summary of main and sensitivity analyses on the associations between short-term exposure to air pollutants and IL-8. **Supplementary Figure 4.** Summary of main and sensitivity analyses on the associations between short-term exposure to air pollutants and IL-10. **Supplementary Figure 5.** Summary of main and sensitivity analyses on the associations between short-term exposure to air pollutants and TNF-α. **Supplementary Figure 6.** Summary of main and sensitivity analyses on the associations between short-term exposure to air pollutants and 8-OHdG. **Supplementary Table 1.** Associations between short-term exposure to air pollutants and biomarkers of inflammation and oxidative stress. **Supplementary Table 2.** Summary statistics of the levels of biomarkers after excluding outliers. **Supplementary Table 3.** Associations between ambient air pollutants and biomarkers of inflammation and oxidative stress after excluding outliers. **Supplementary Table 4.** Associations between ambient air pollutants and biomarkers of inflammation and oxidative stress among non-smokers. **Supplementary Table 5-1.** Associations between ambient air pollutants and IL-1β in the two-pollutant model. **Supplementary Table 5-2.** Associations between ambient air pollutants and IL-6 in the two-pollutant model. **Supplementary Table 5-3.** Associations between ambient air pollutants and IL-8 in the two-pollutant model. **Supplementary Table 5-4.** Associations between ambient air pollutants and IL-10 in the two-pollutant model. **Supplementary Table 5-5.** Associations between ambient air pollutants and TNF-α in the two-pollutant model. **Supplementary Table 5-6.** Associations between ambient air pollutants and urinary 8-OHdG in the two-pollutant model.

## Declarations

### Availability of data and material

The data that support the findings of this study are available from the corresponding author upon reasonable request.
