# Supplementary material for: Association between short-term exposure to ambient air pollutants and biomarkers indicative of inflammation and oxidative stress: a cross-sectional study using KoGES-HEXA data
Source: Environ Health Prev Med. 2024 Mar 16;29:17. doi: 10.1265/ehpm.23-00199 (PMC10957338; doi:10.1265/ehpm.23-00199)
Supplement: Supplementary file 1 — Additional file 1: Supplementary Figure 1. Summary of main and sensitivity analyses on the associations between short-term exposure to air pollutants and IL-1β. Supplementary Figure 2. Summary of main and sensitivity analyses on the associations between short-term exposure to air pollutants and IL-6. Supplementary Figure 3. Summary of main and sensitivity analyses on the associations between short-term exposure to air pollutants and IL-8. Supplementary Figure 4. Summary of main and sensitivity analyses on the associations between short-term exposure to air pollutants and IL-10. Supplementary Figure 5. Summary of main and sensitivity analyses on the associations between short-term exposure to air pollutants and TNF-α. Supplementary Figure 6. Summary of main and sensitivity analyses on the associations between short-term exposure to air pollutants and 8-OHdG. Supplementary Table 1. Associations between short-term exposure to air pollutants and biomarkers of inflammation and oxidative stress. Supplementary Table 2. Summary statistics of the levels of biomarkers after excluding outliers. Supplementary Table 3. Associations between ambient air pollutants and biomarkers of inflammation and oxidative stress after excluding outliers. Supplementary Table 4. Associations between ambient air pollutants and biomarkers of inflammation and oxidative stress among non-smokers. Supplementary Table 5-1. Associations between ambient air pollutants and IL-1β in the two-pollutant model. Supplementary Table 5-2. Associations between ambient air pollutants and IL-6 in the two-pollutant model. Supplementary Table 5-3. Associations between ambient air pollutants and IL-8 in the two-pollutant model. Supplementary Table 5-4. Associations between ambient air pollutants and IL-10 in the two-pollutant model. Supplementary Table 5-5. Associations between ambient air pollutants and TNF-α in the two-pollutant model. Supplementary Table 5-6. Associations between ambient air pollutants and urinary 8- [file ehpm-29-017-s001.docx]

**Association between short-term exposure to ambient air pollutants and biomarkers indicative of inflammation and oxidative stress: a cross-sectional study using KoGES-HEXA data**

Ji Hyun Kim, Hae Dong Woo, Jane J Lee, Dae Sub Song and Kyoungho Lee^*^

**[Table of Contents]**

**Supplementary Figures**

Supplementary Figure 1. Summary of main and sensitivity analyses on the associations between short-term exposure to air pollutants and IL-1β

Supplementary Figure 2. Summary of main and sensitivity analyses on the associations between short-term exposure to air pollutants and IL-6

Supplementary Figure 3. Summary of main and sensitivity analyses on the associations between short-term exposure to air pollutants and IL-8

Supplementary Figure 4. Summary of main and sensitivity analyses on the associations between short-term exposure to air pollutants and IL-10

Supplementary Figure 5. Summary of main and sensitivity analyses on the associations between short-term exposure to air pollutants and TNF-α

Supplementary Figure 6. Summary of main and sensitivity analyses on the associations between short-term exposure to air pollutants and 8-OHdG

**Supplementary Tables**

Supplementary Table 1. Associations between short-term exposure to air pollutants and biomarkers of inflammation and oxidative stress

Supplementary Table 2. Summary statistics of the levels of biomarkers after excluding outliers

Supplementary Table 3. Associations between ambient air pollutants and biomarkers of inflammation and oxidative stress after excluding outliers

Supplementary Table 4. Associations between ambient air pollutants and biomarkers of inflammation and oxidative stress among non-smokers

Supplementary Table 5-1. Associations between ambient air pollutants and IL-1β in the two-pollutant model

Supplementary Table 5-2. Associations between ambient air pollutants and IL-6 in the two-pollutant model

Supplementary Table 5-3. Associations between ambient air pollutants and IL-8 in the two-pollutant model

Supplementary Table 5-4. Associations between ambient air pollutants and IL-10 in the two-pollutant model

Supplementary Table 5-5. Associations between ambient air pollutants and TNF-α in the two-pollutant model

Supplementary Table 5-6. Associations between ambient air pollutants and urinary 8-OHdG in the two-pollutant model

| 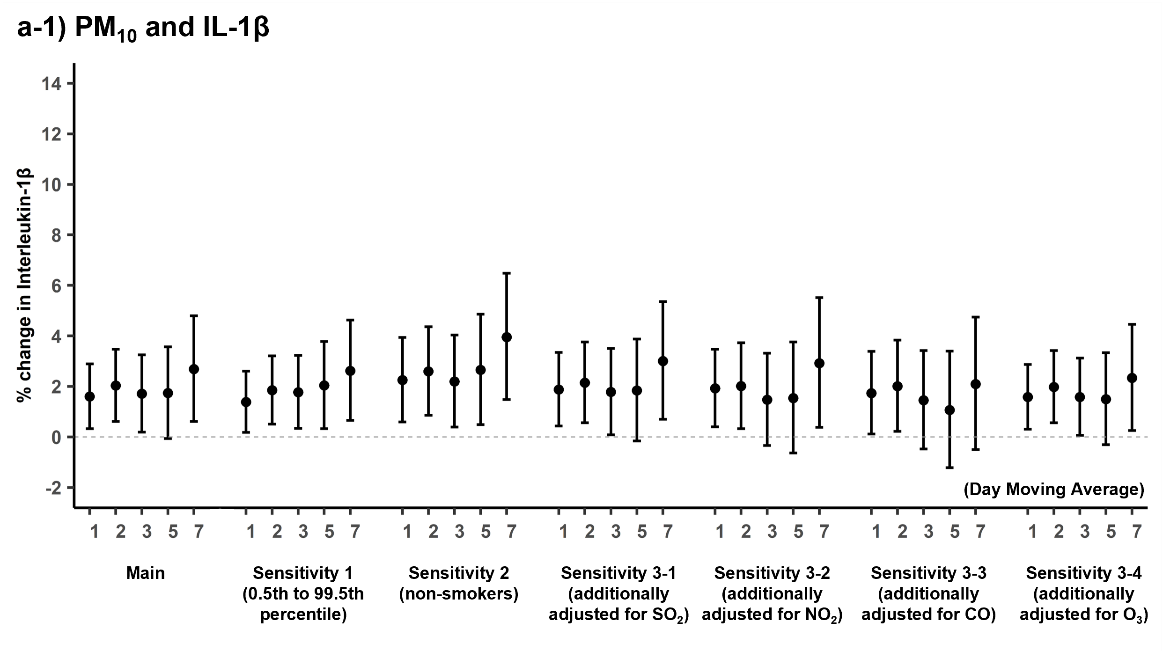 | 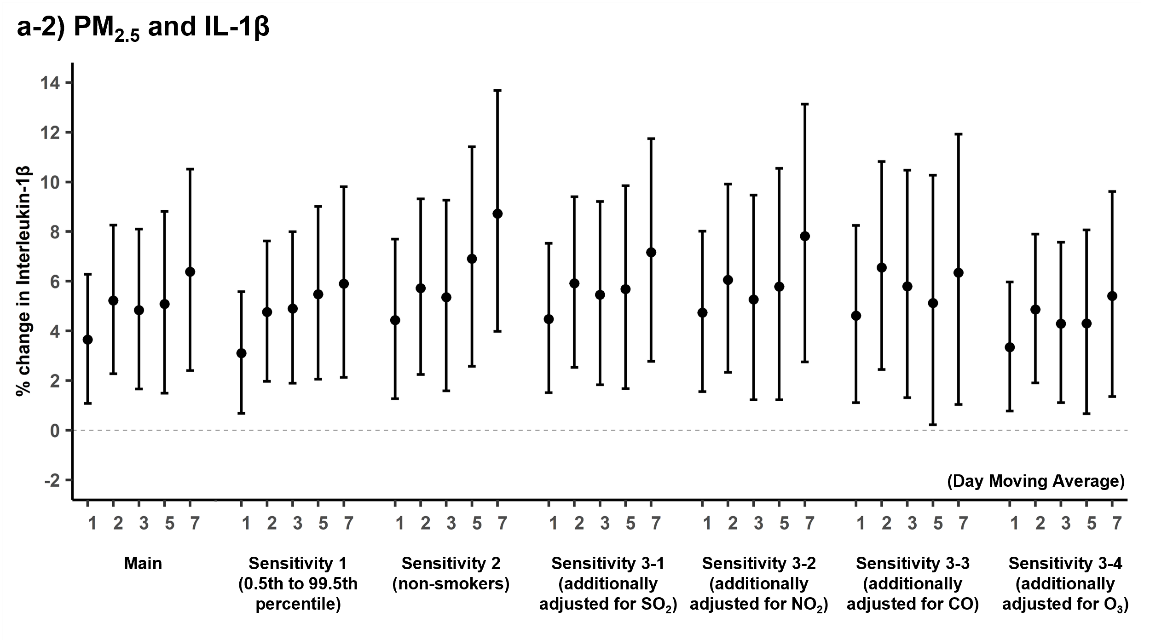 |
| --- | --- |
| 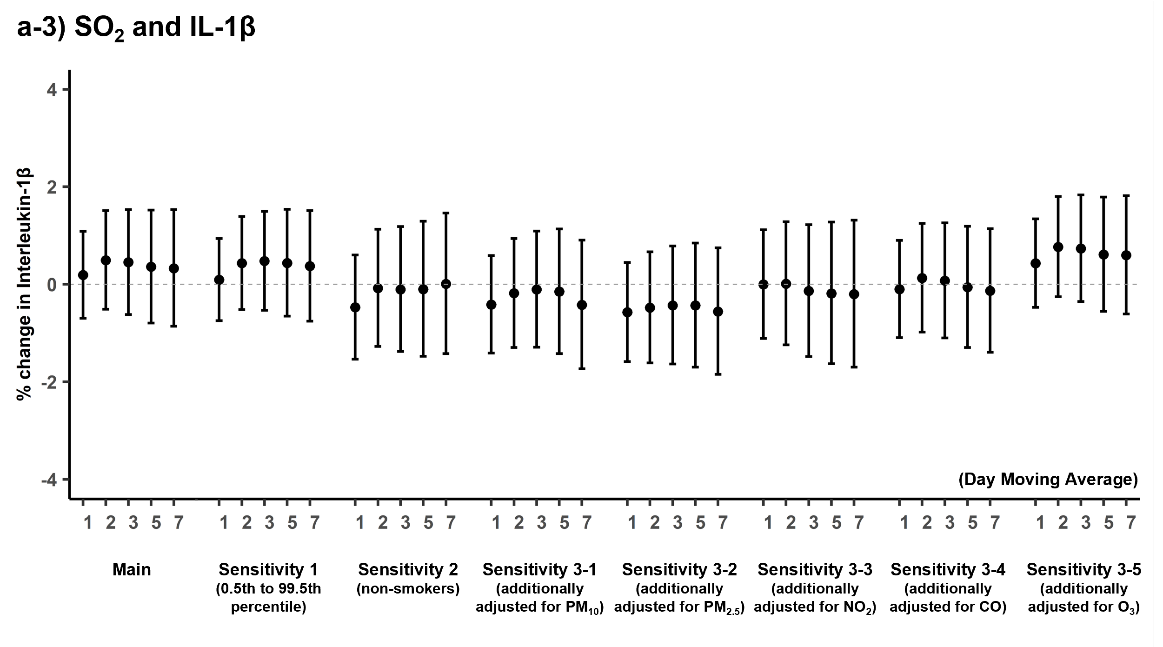 | 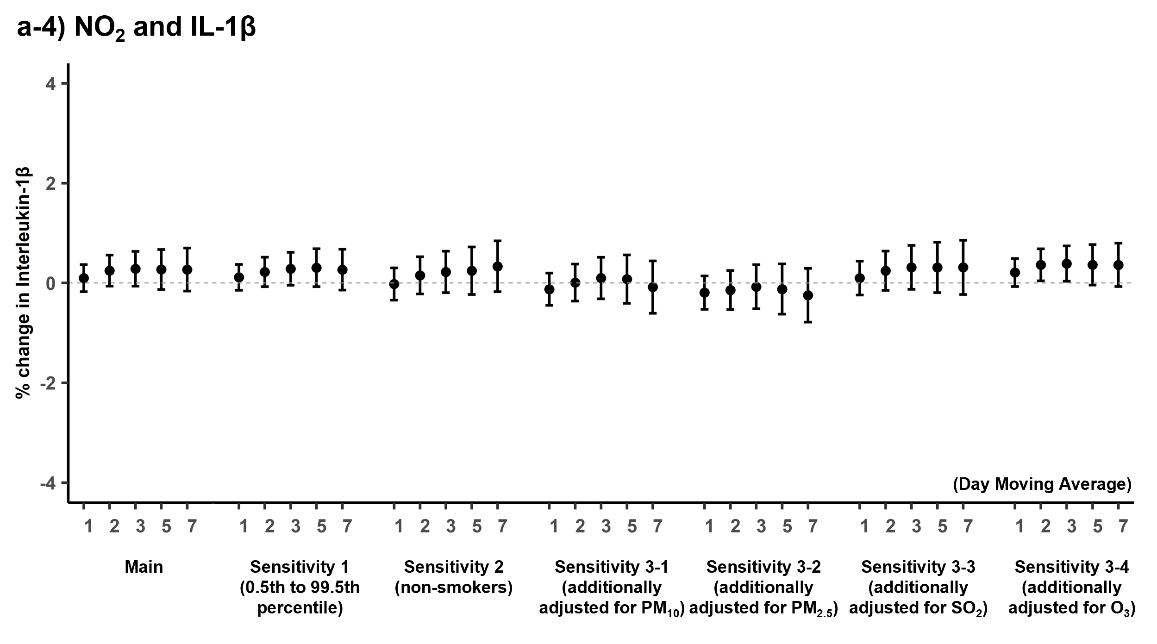 |

**Supplementary Figure 1.** Summary of main and sensitivity analyses on the associations between short-term exposure to air pollutants and IL-1β. The models are adjusted for date of examination as a linear term, continuous variables of age, body mass index, and the corresponding moving averages of temperature and relative humidity; and categorical variables of season, weekday of examination, residential region, sex, smoking history, alcohol consumption status, regular exercise, education, occupational status, and marital status. The sensitivity analysis on non-smokers did not account for smoking history; and two-pollutant model additionally controlled for the other air pollutant shown above. Estimates are presented as percentage changes with 95% confidence intervals in each biomarker level per 1-unit increase in 1- to 7-day average ambient air pollution exposure (units for PM: 10 μg/m^3^; SO_2_, NO_2_, and O_3_: 1 ppb; and CO: 0.1 ppm). PM_10_, PM_2.5_: particulate matter with aerodynamic diameter < 10 μm and < 2.5 μm, respectively; SO_2_: sulfur dioxide; NO_2_: nitrogen dioxide; CO: carbon monoxide; O_3_: ozone; IL: interleukin.

| 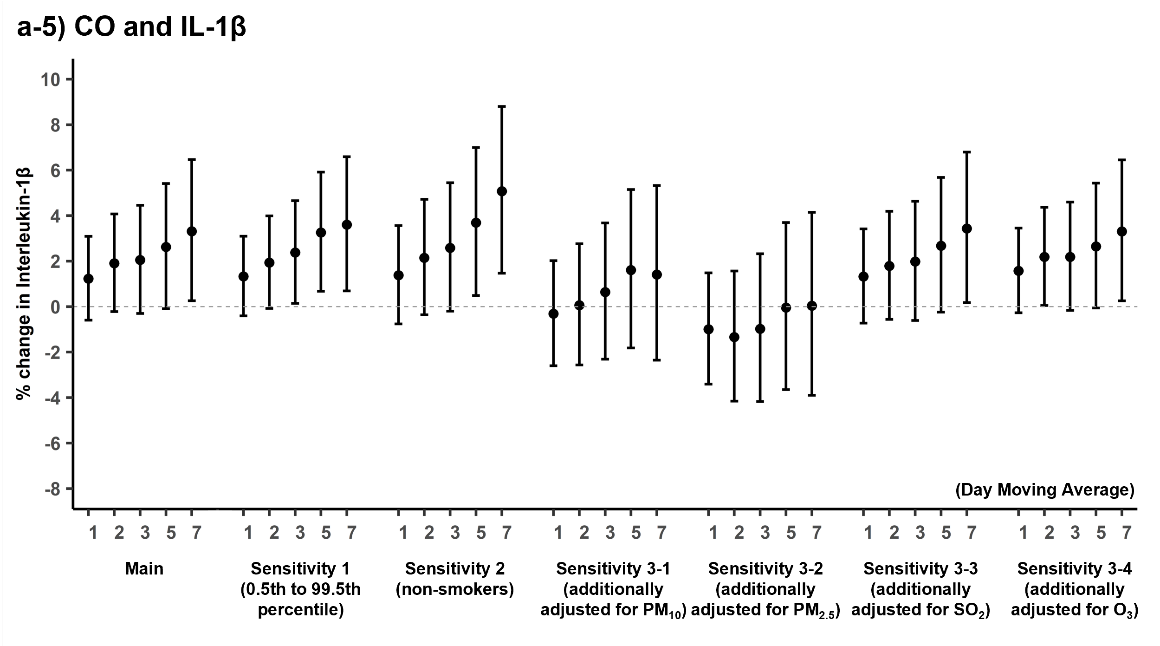 | 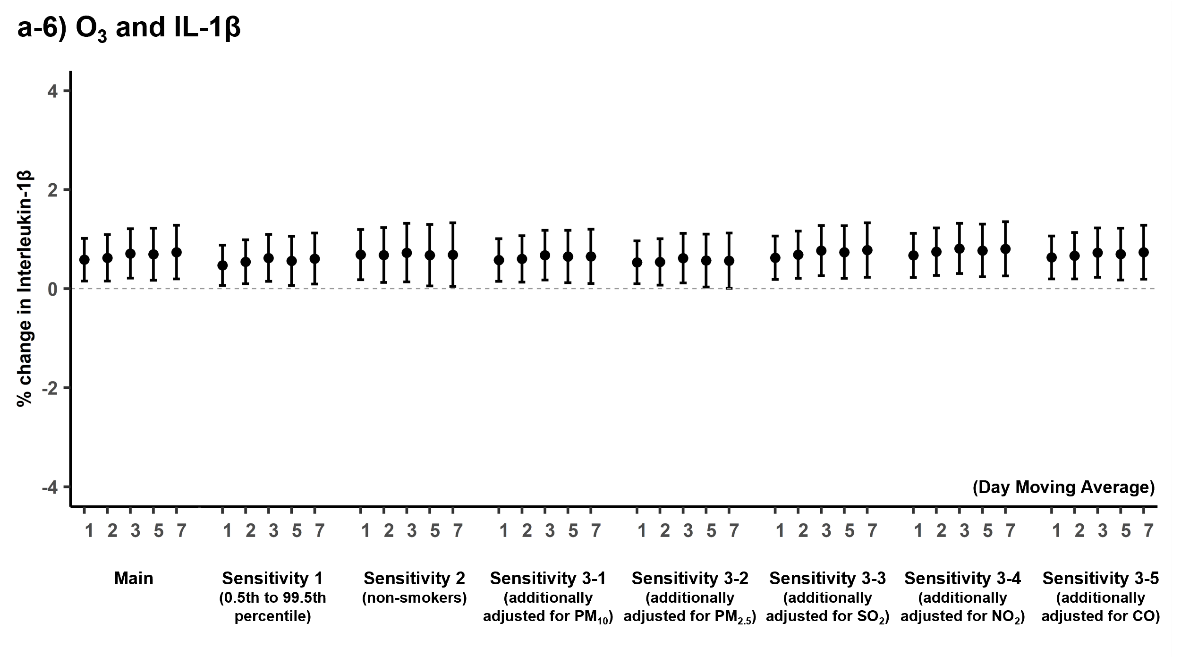 |
| --- | --- |

**Supplementary Figure 1.** Cont’d.

| 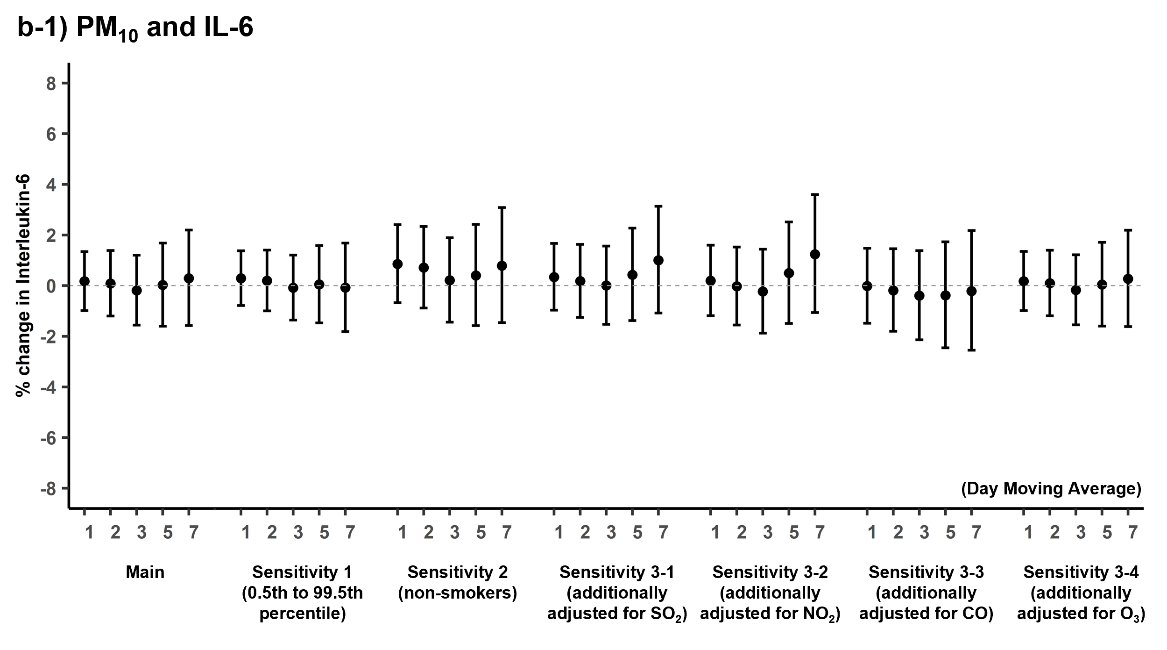 | 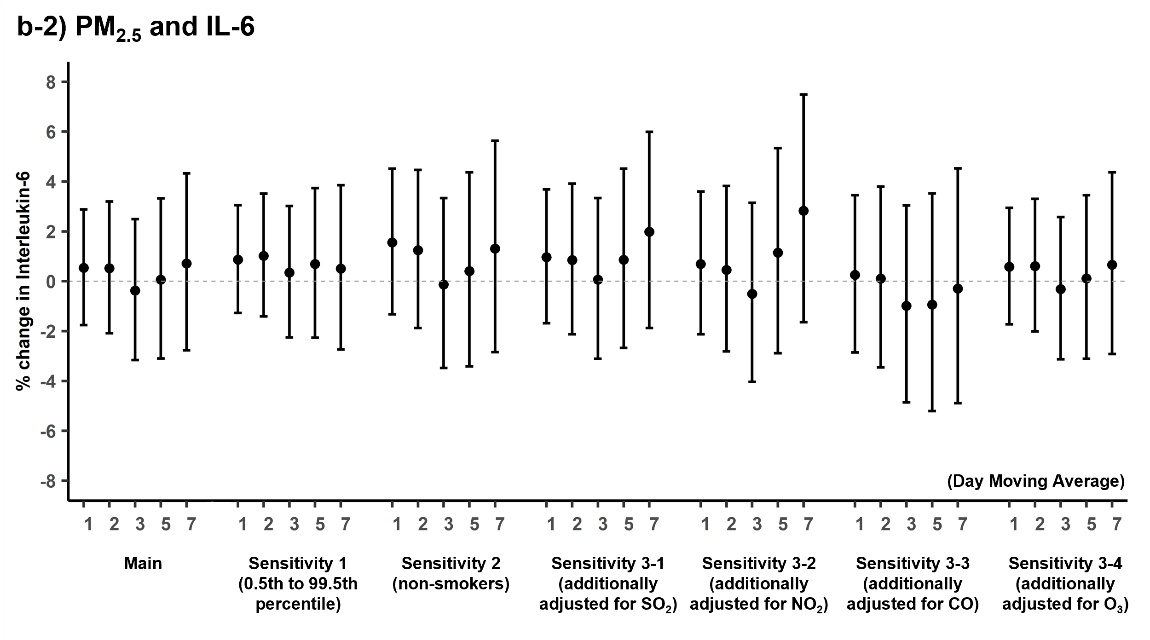 |
| --- | --- |
| 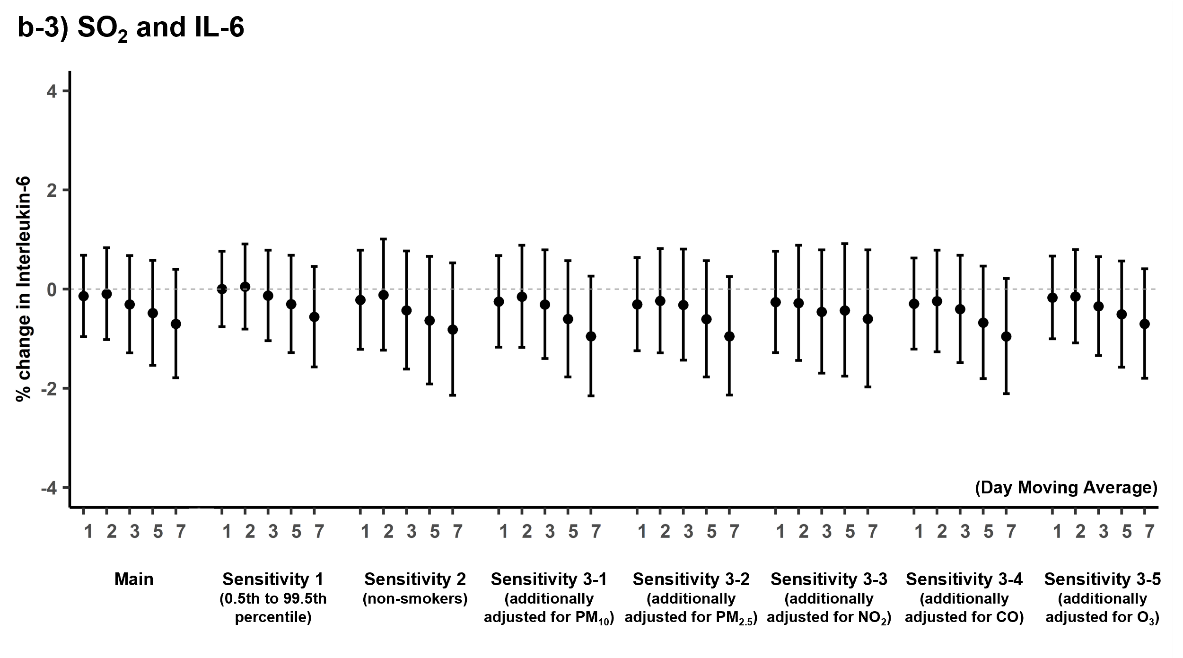 | 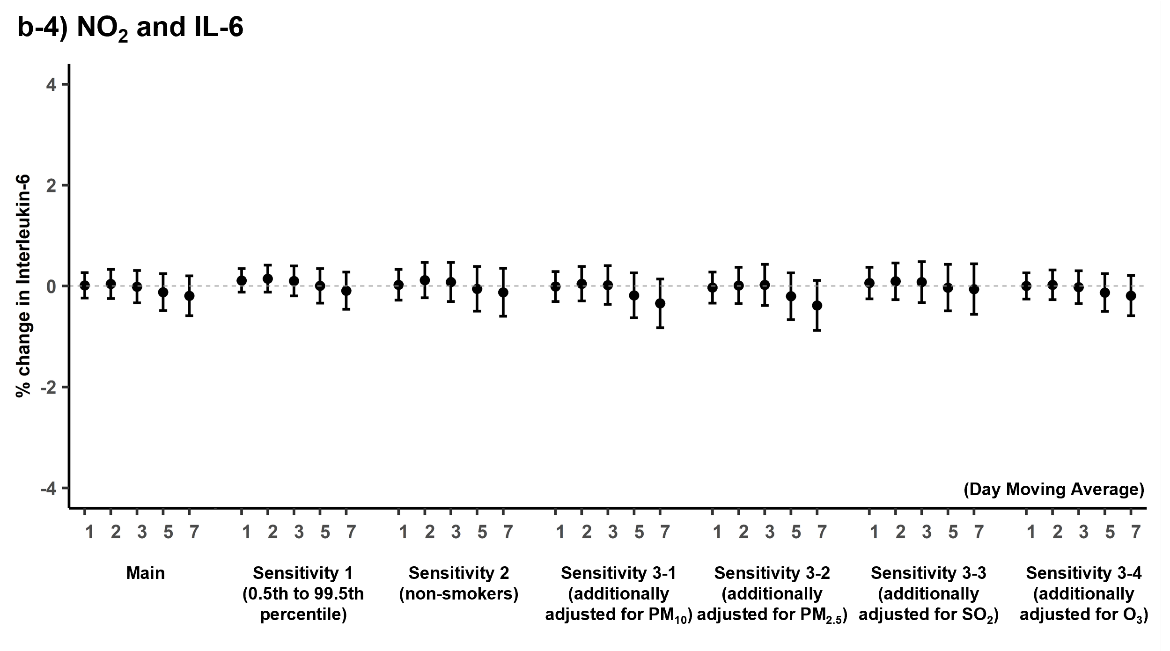 |

**Supplementary Figure 2.** Summary of main and sensitivity analyses on the associations between short-term exposure to air pollutants and IL-6. The models are adjusted for date of examination as a linear term, continuous variables of age, body mass index, and the corresponding moving averages of temperature and relative humidity; and categorical variables of season, weekday of examination, residential region, sex, smoking history, alcohol consumption status, regular exercise, education, occupational status, and marital status. The sensitivity analysis on non-smokers did not account for smoking history; and two-pollutant model additionally controlled for the other air pollutant shown above. Estimates are presented as percentage changes with 95% confidence intervals in each biomarker level per 1-unit increase in 1- to 7-day average ambient air pollution exposure (units for PM: 10 μg/m^3^; SO_2_, NO_2_, and O_3_: 1 ppb; and CO: 0.1 ppm). PM_10_, PM_2.5_: particulate matter with aerodynamic diameter < 10 μm and < 2.5 μm, respectively; SO_2_: sulfur dioxide; NO_2_: nitrogen dioxide; CO: carbon monoxide; O_3_: ozone; IL: interleukin.

| 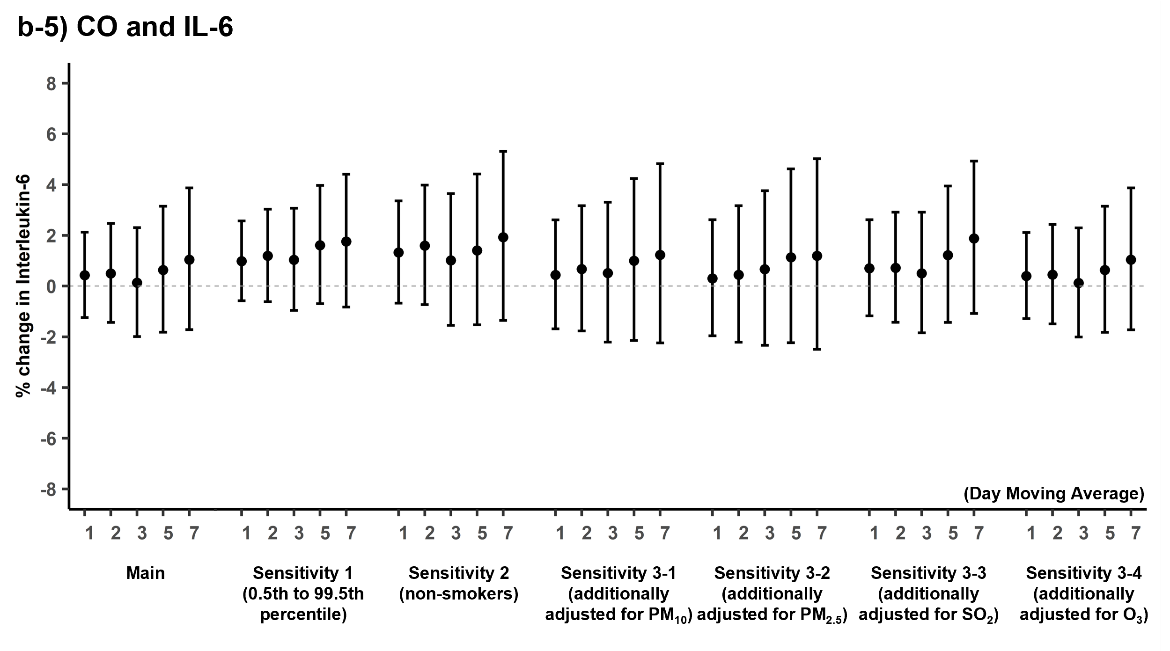 | 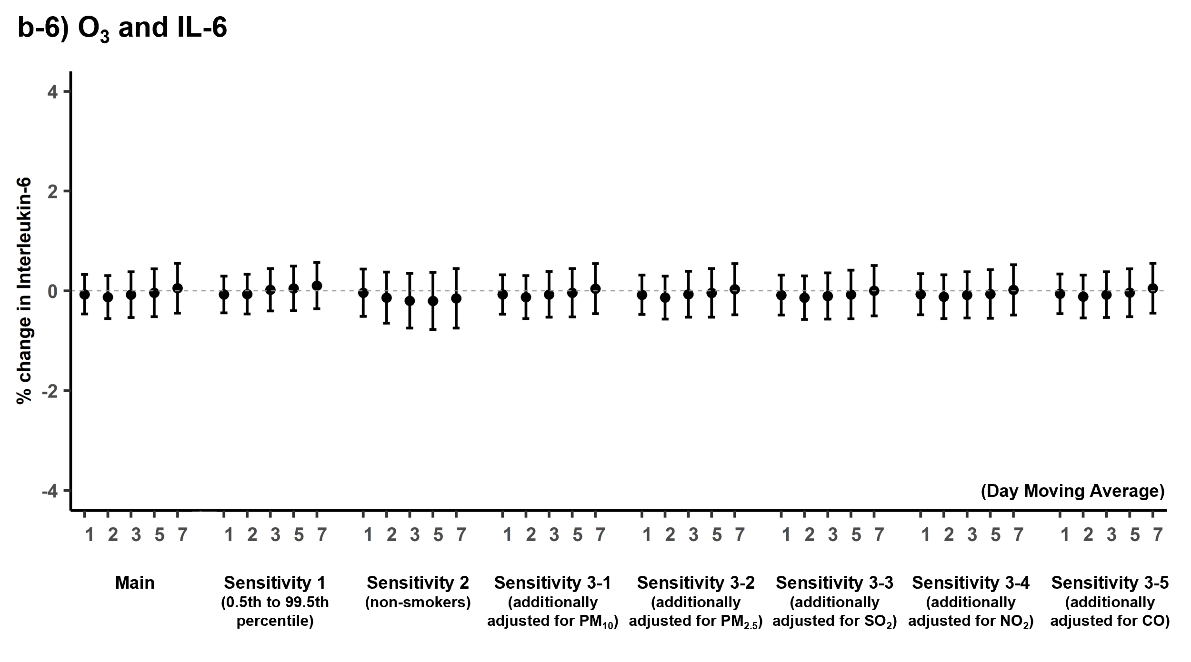 |
| --- | --- |

**Supplementary Figure 2.** Cont’d.

| 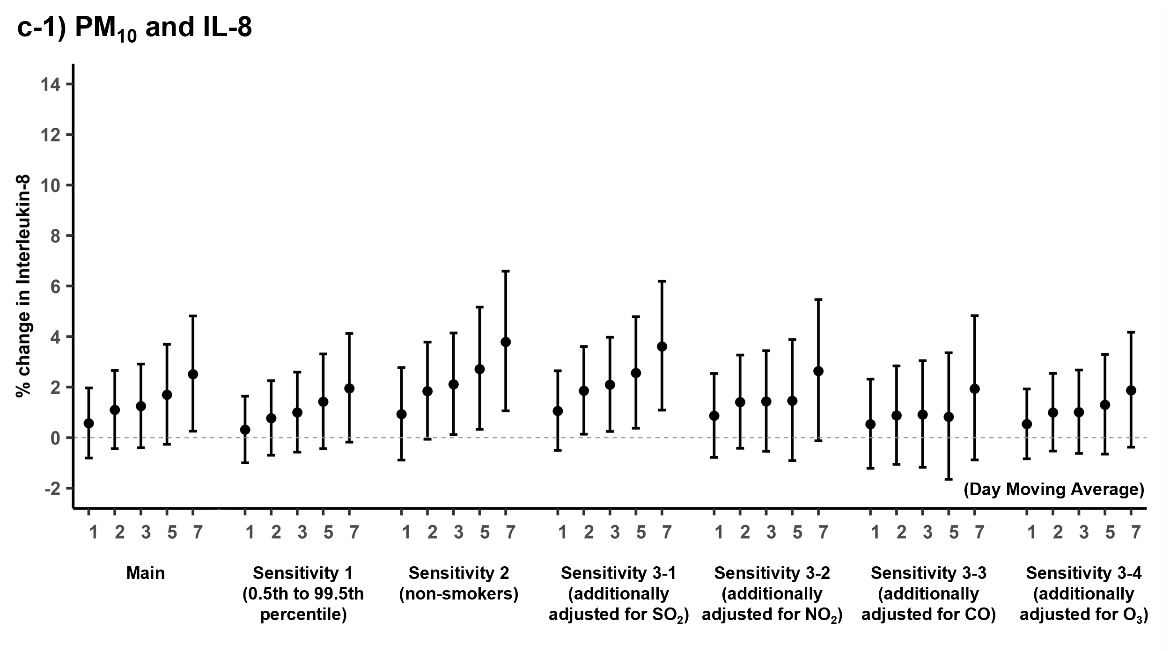 | 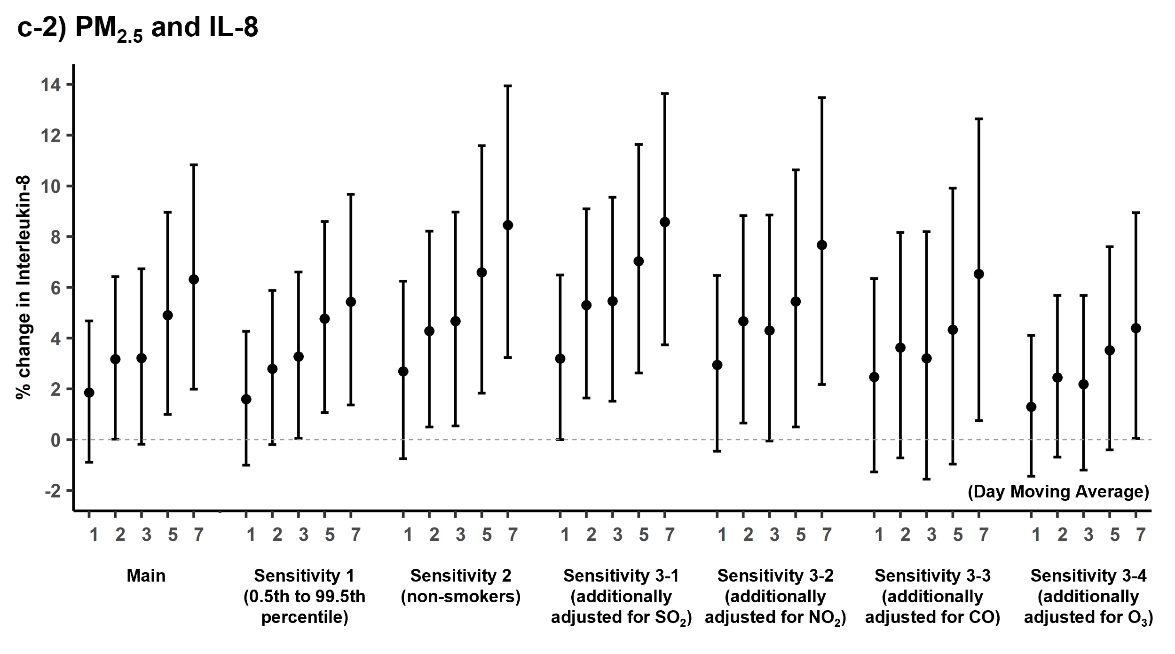 |
| --- | --- |
| 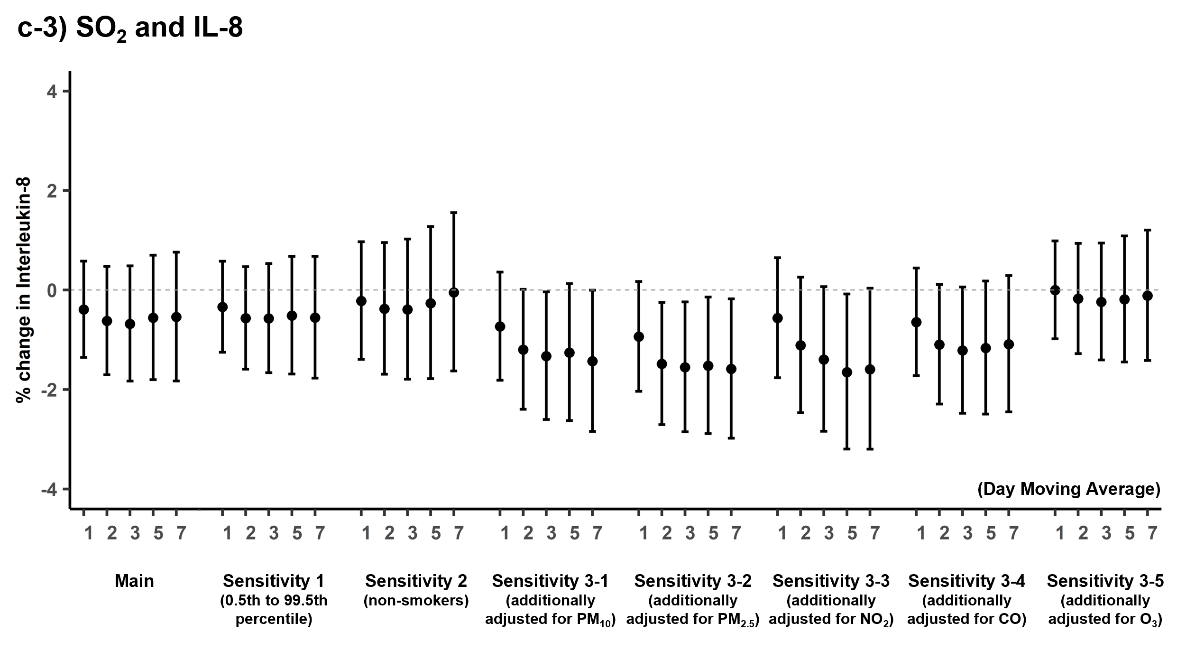 | 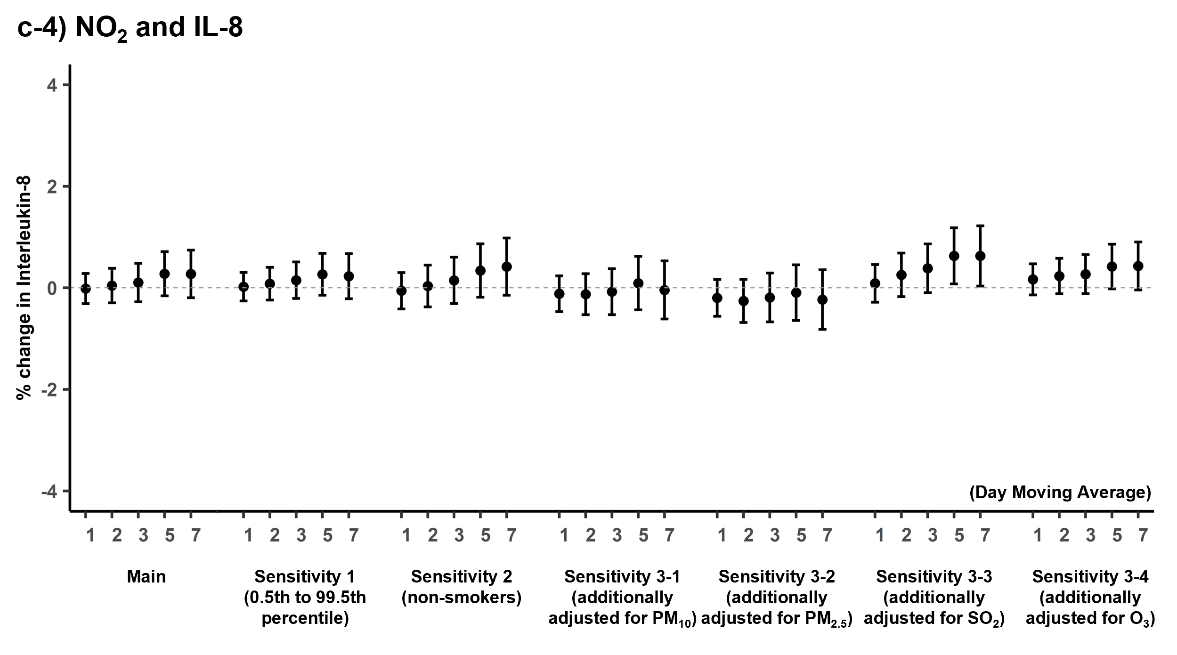 |

**Supplementary Figure 3.** Summary of main and sensitivity analyses on the associations between short-term exposure to air pollutants and IL-8. The models are adjusted for date of examination as a linear term, continuous variables of age, body mass index, and the corresponding moving averages of temperature and relative humidity; and categorical variables of season, weekday of examination, residential region, sex, smoking history, alcohol consumption status, regular exercise, education, occupational status, and marital status. The sensitivity analysis on non-smokers did not account for smoking history; and two-pollutant model additionally controlled for the other air pollutant shown above. Estimates are presented as percentage changes with 95% confidence intervals in each biomarker level per 1-unit increase in 1- to 7-day average ambient air pollution exposure (units for PM: 10 μg/m^3^; SO_2_, NO_2_, and O_3_: 1 ppb; and CO: 0.1 ppm). PM_10_, PM_2.5_: particulate matter with aerodynamic diameter < 10 μm and < 2.5 μm, respectively; SO_2_: sulfur dioxide; NO_2_: nitrogen dioxide; CO: carbon monoxide; O_3_: ozone; IL: interleukin.

| 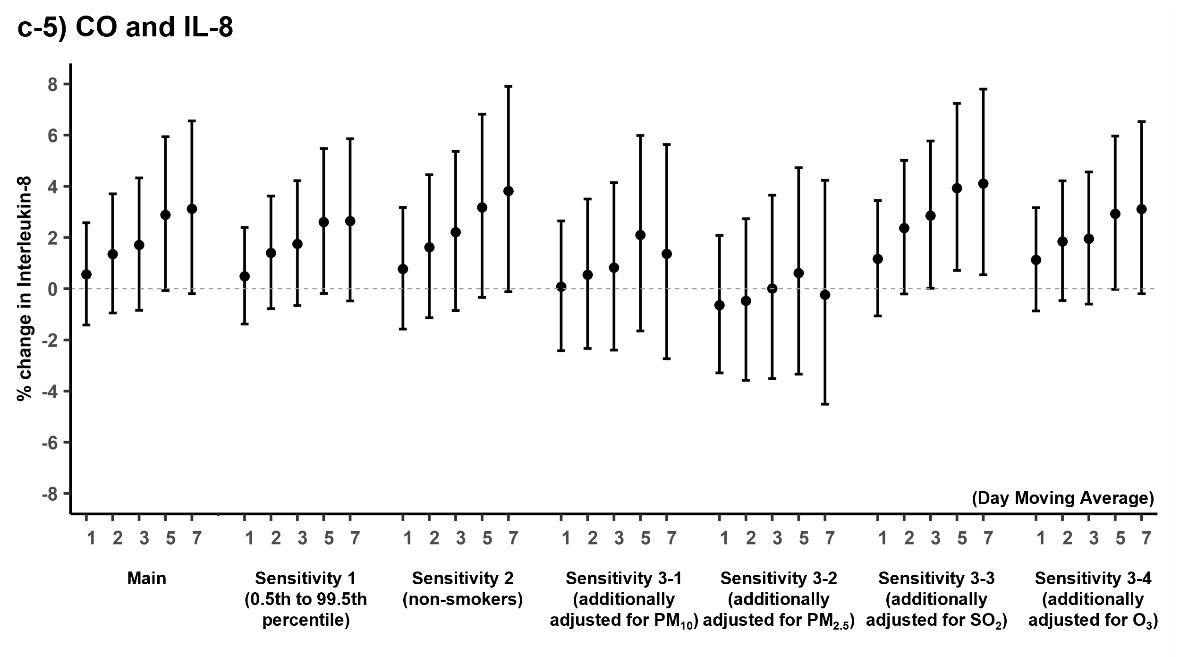 | 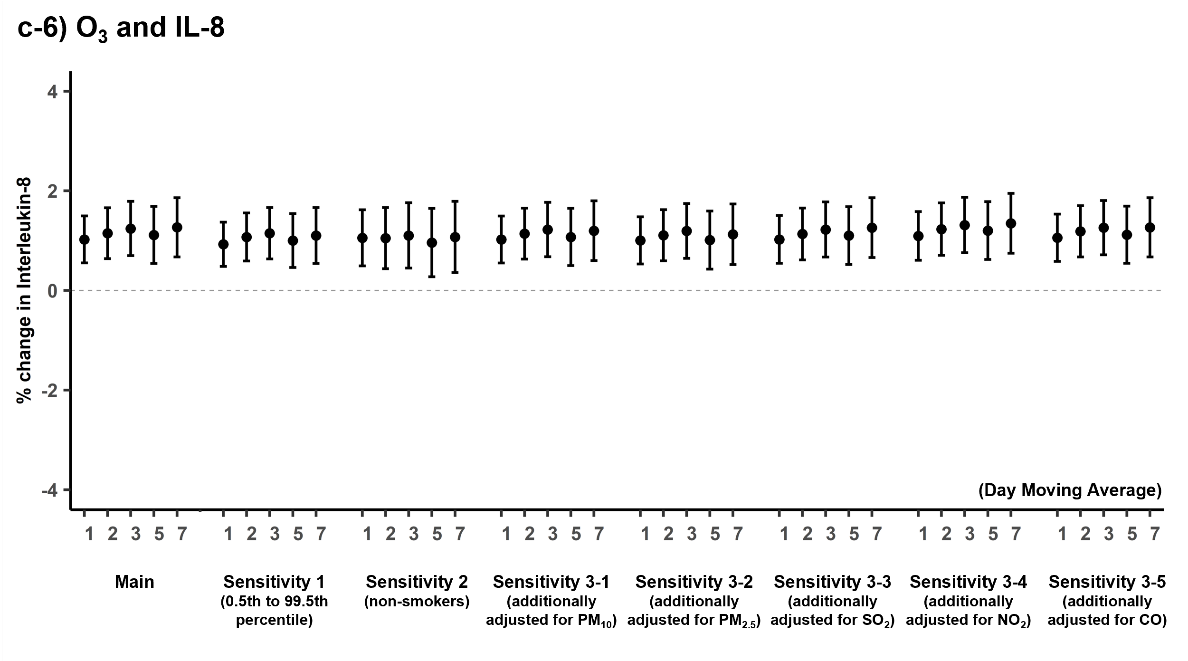 |
| --- | --- |

**Supplementary Figure 3.** Cont’d.

| 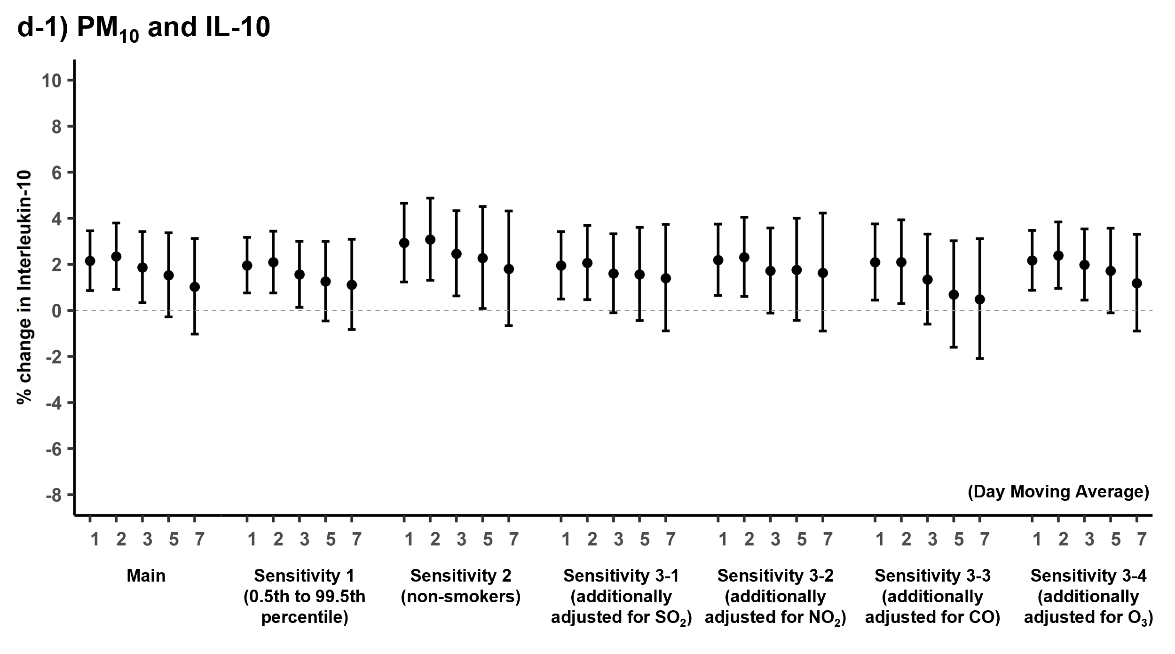 | 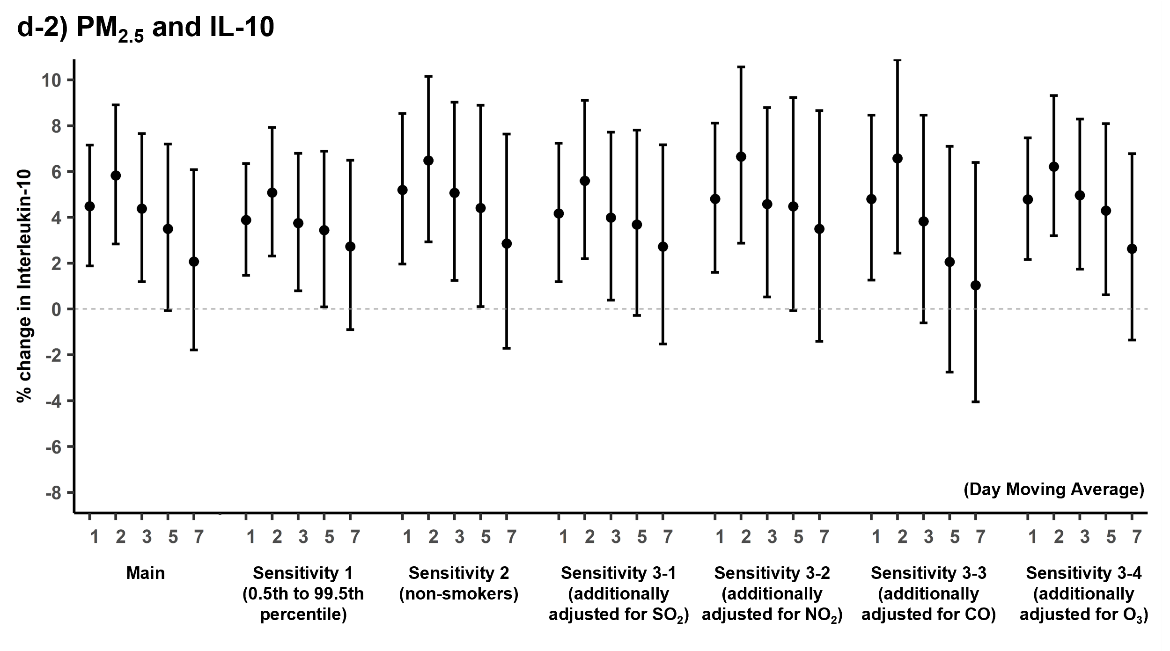 |
| --- | --- |
| 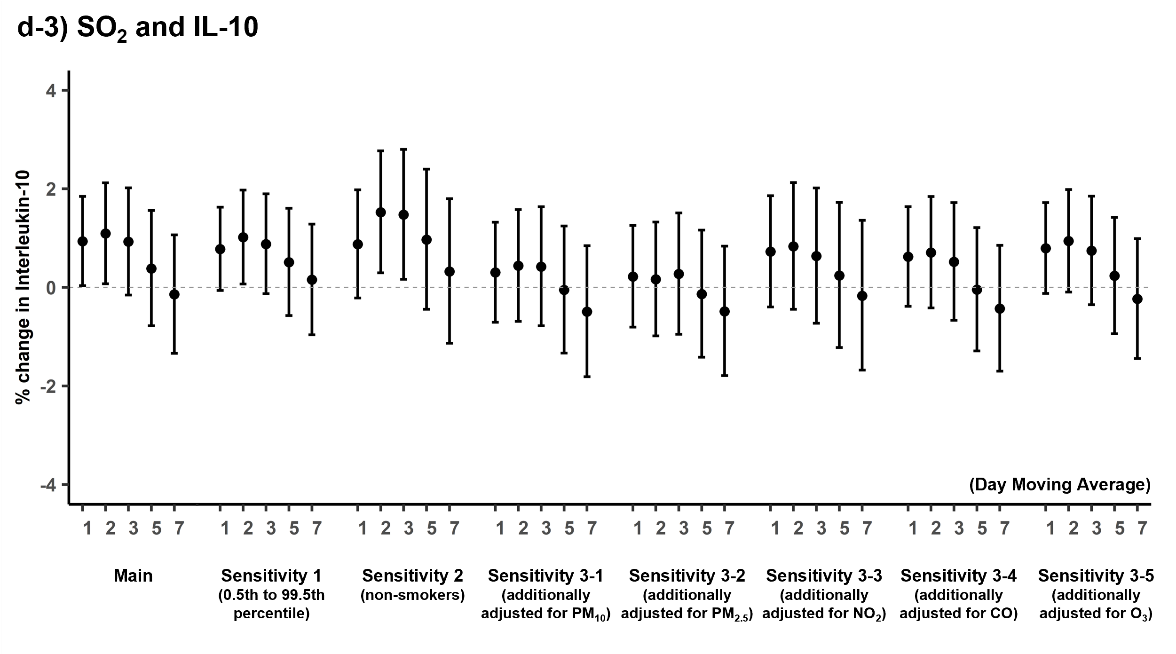 | 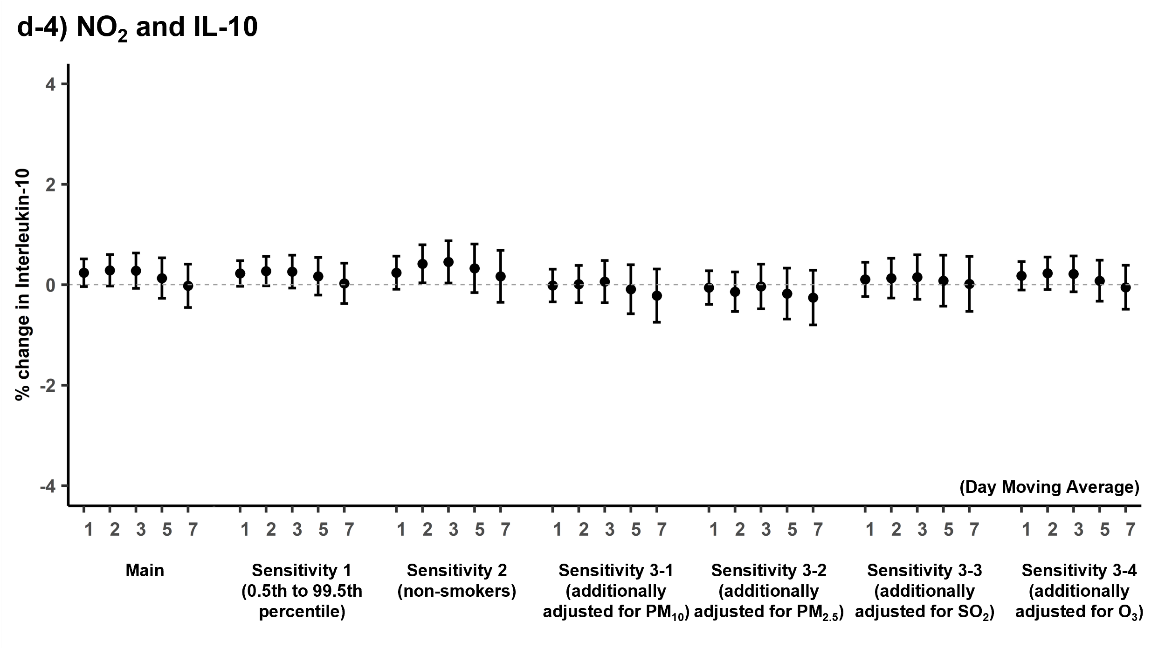 |

**Supplementary Figure 4.** Summary of main and sensitivity analyses on the associations between short-term exposure to air pollutants and IL-10. The models are adjusted for date of examination as a linear term, continuous variables of age, body mass index, and the corresponding moving averages of temperature and relative humidity; and categorical variables of season, weekday of examination, residential region, sex, smoking history, alcohol consumption status, regular exercise, education, occupational status, and marital status. The sensitivity analysis on non-smokers did not account for smoking history; and two-pollutant model additionally controlled for the other air pollutant shown above. Estimates are presented as percentage changes with 95% confidence intervals in each biomarker level per 1-unit increase in 1- to 7-day average ambient air pollution exposure (units for PM: 10 μg/m^3^; SO_2_, NO_2_, and O_3_: 1 ppb; and CO: 0.1 ppm). PM_10_, PM_2.5_: particulate matter with aerodynamic diameter < 10 μm and < 2.5 μm, respectively; SO_2_: sulfur dioxide; NO_2_: nitrogen dioxide; CO: carbon monoxide; O_3_: ozone; IL: interleukin.

| 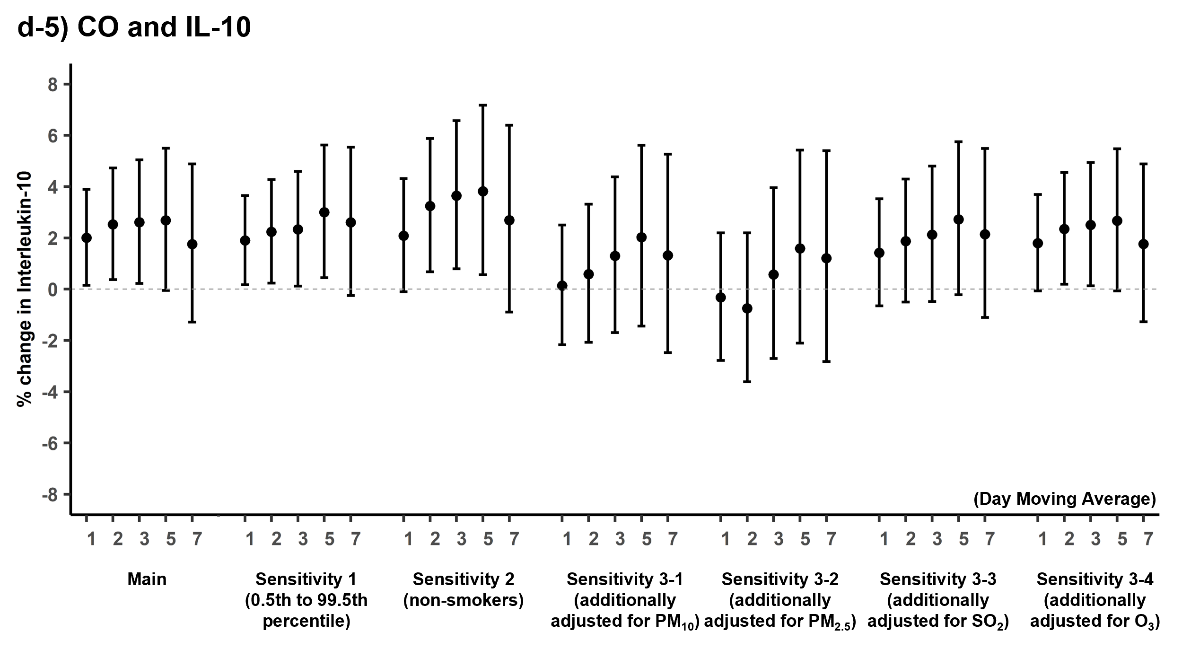 | 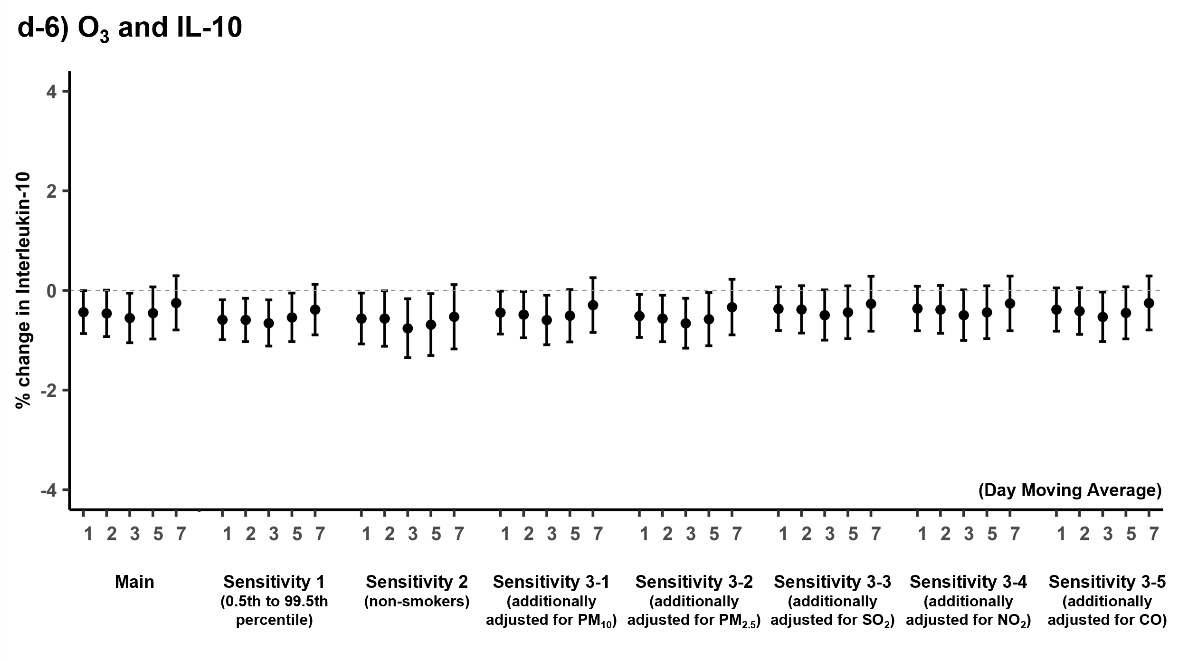 |
| --- | --- |

**Supplementary Figure 4.** Cont’d.

| 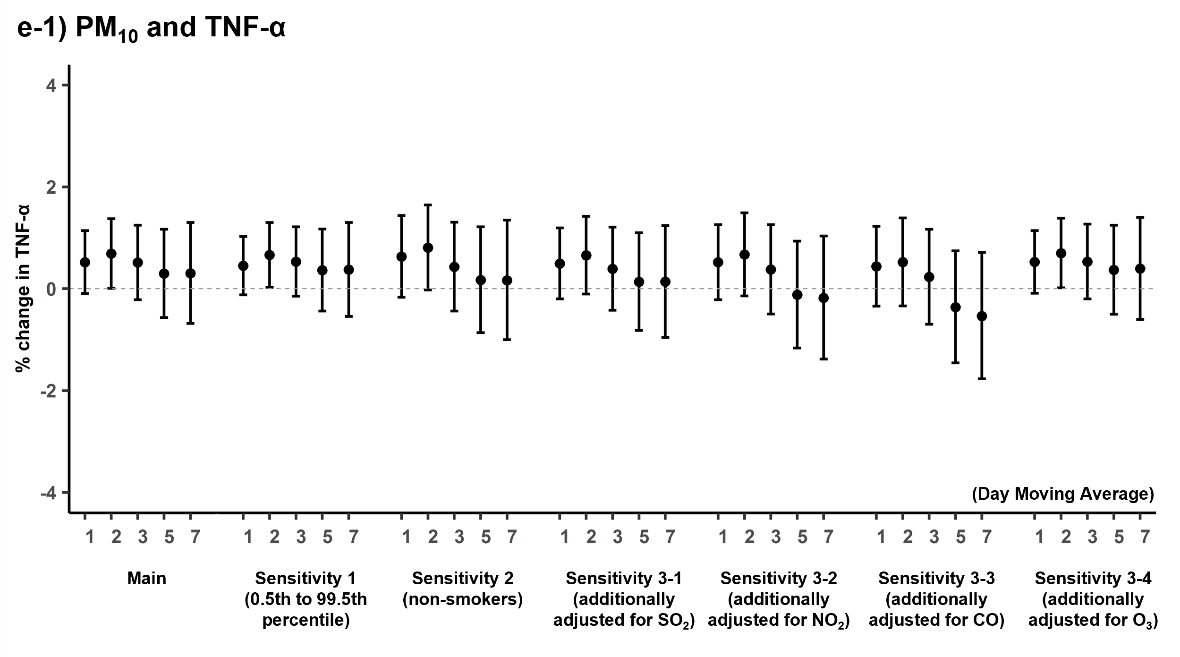 | 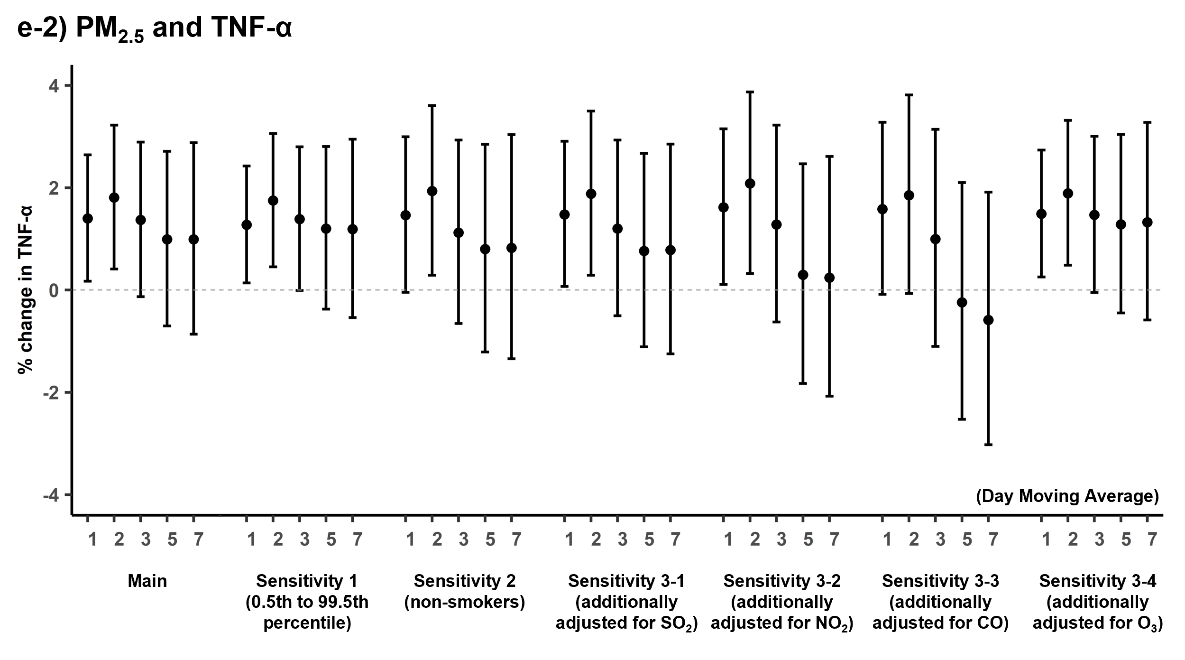 |
| --- | --- |
| 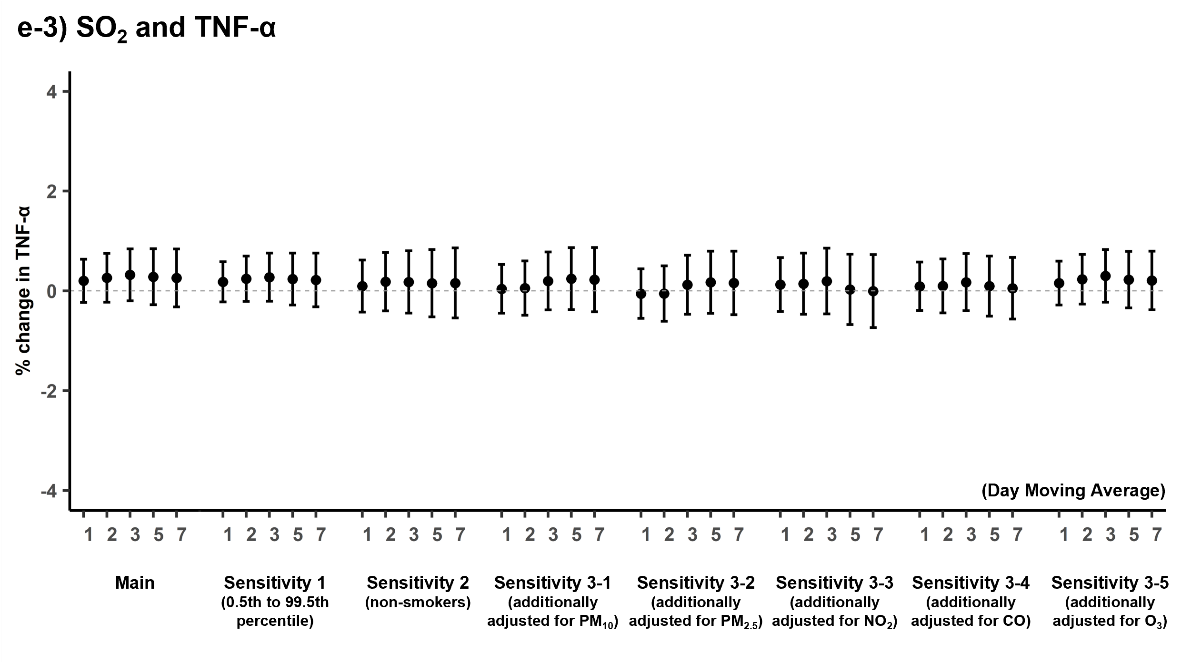 | 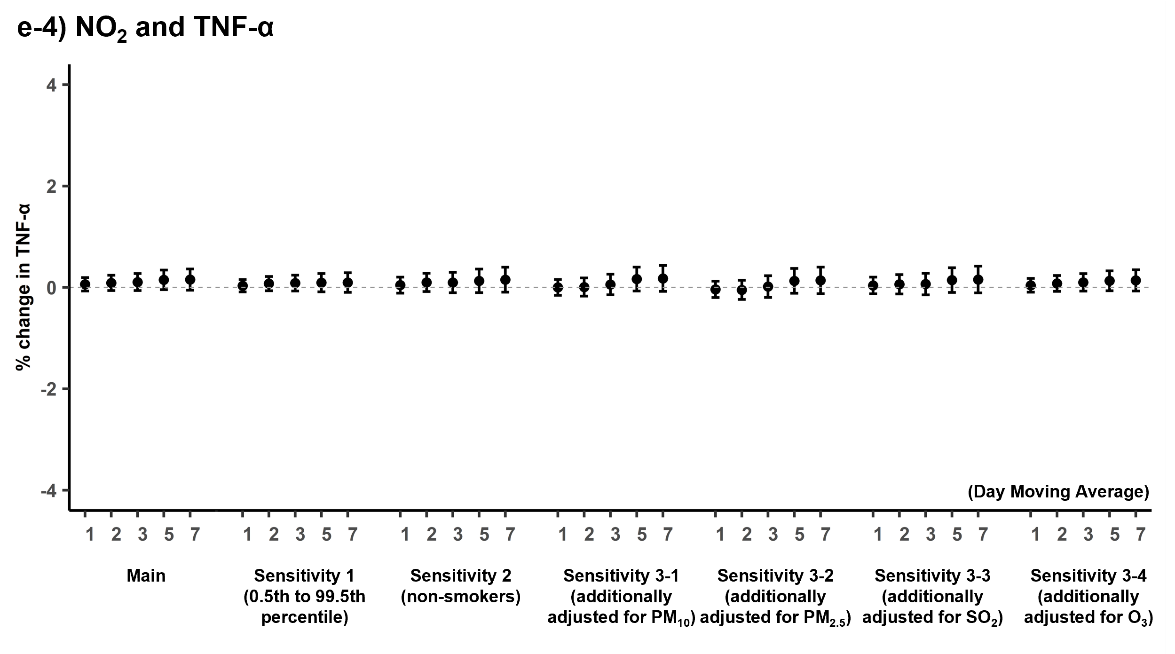 |

**Supplementary Figure 5.** Summary of main and sensitivity analyses on the associations between short-term exposure to air pollutants and TNF-α. The models are adjusted for date of examination as a linear term, continuous variables of age, body mass index, and the corresponding moving averages of temperature and relative humidity; and categorical variables of season, weekday of examination, residential region, sex, smoking history, alcohol consumption status, regular exercise, education, occupational status, and marital status. The sensitivity analysis on non-smokers did not account for smoking history; and two-pollutant model additionally controlled for the other air pollutant shown above. Estimates are presented as percentage changes with 95% confidence intervals in each biomarker level per 1-unit increase in 1- to 7-day average ambient air pollution exposure (units for PM: 10 μg/m^3^; SO_2_, NO_2_, and O_3_: 1 ppb; and CO: 0.1 ppm). PM_10_, PM_2.5_: particulate matter with aerodynamic diameter < 10 μm and < 2.5 μm, respectively; SO_2_: sulfur dioxide; NO_2_: nitrogen dioxide; CO: carbon monoxide; O_3_: ozone; TNF: tumor necrosis factor.

| 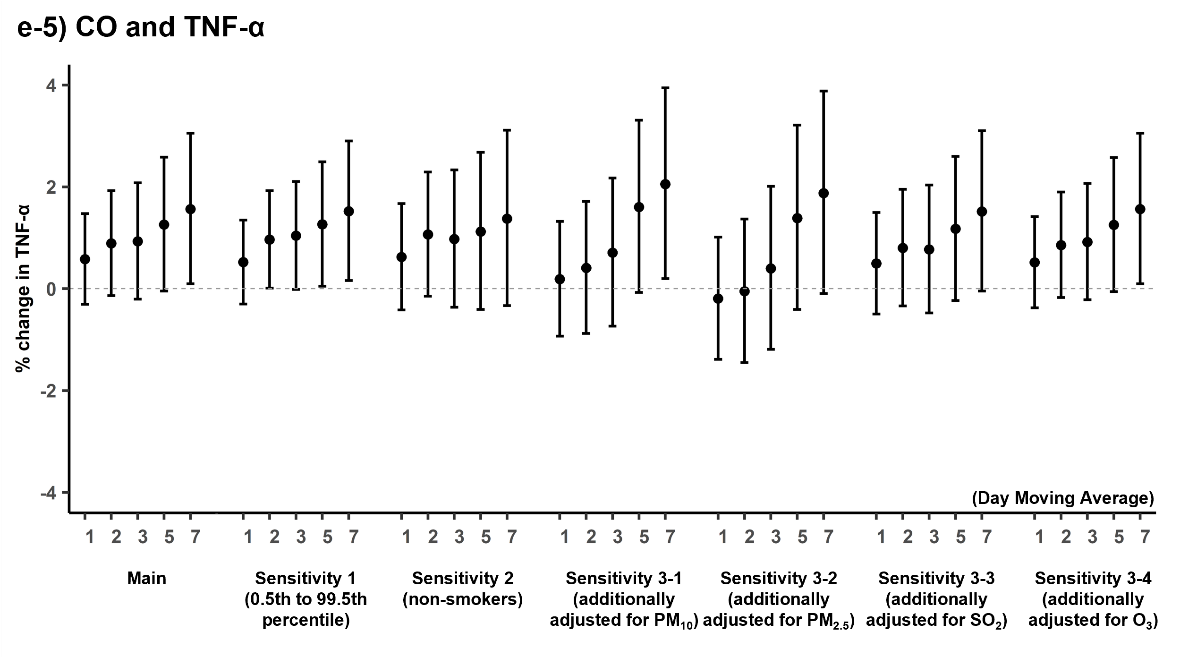 | 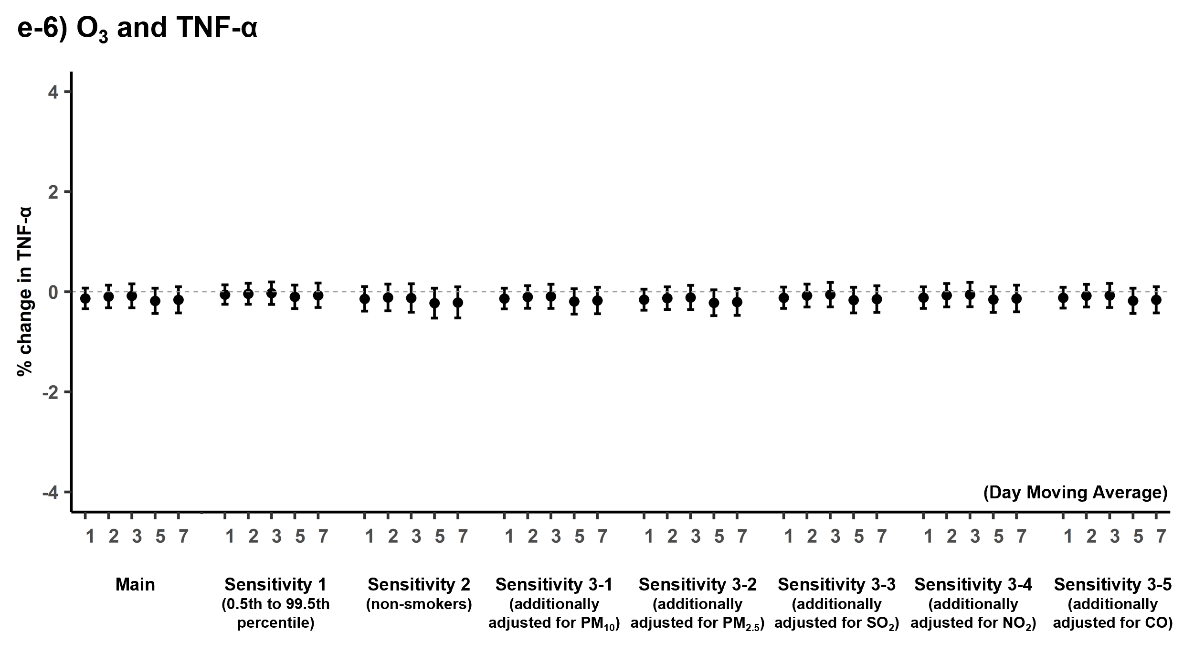 |
| --- | --- |

**Supplementary Figure 5.** Cont’d.

| 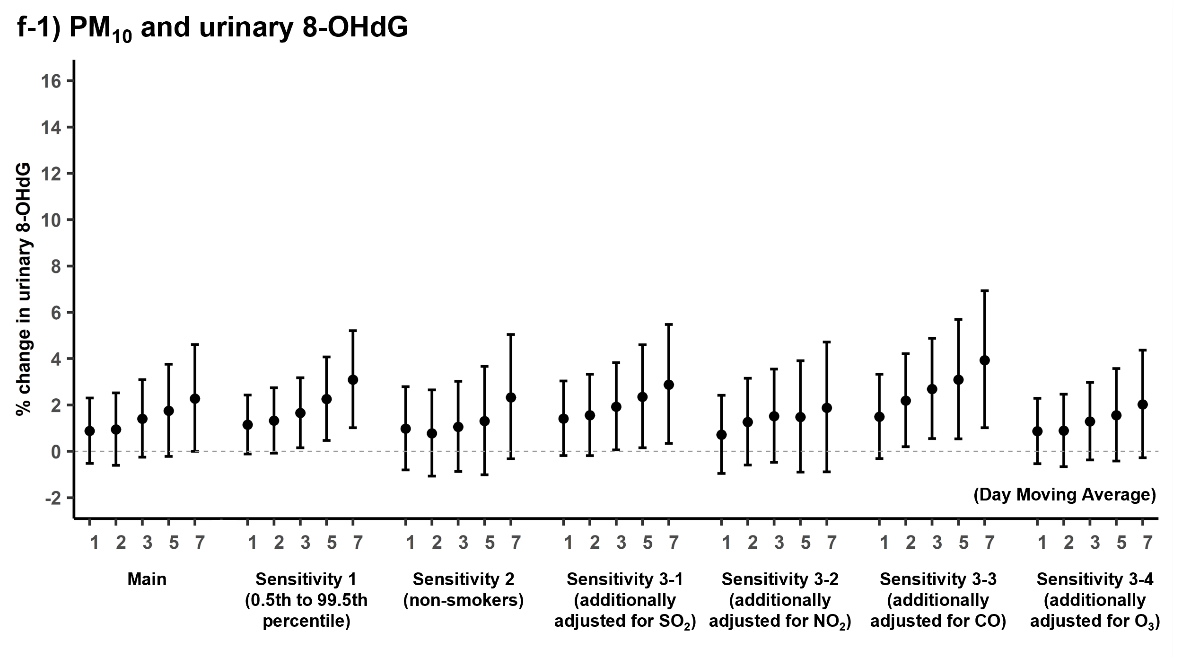 | 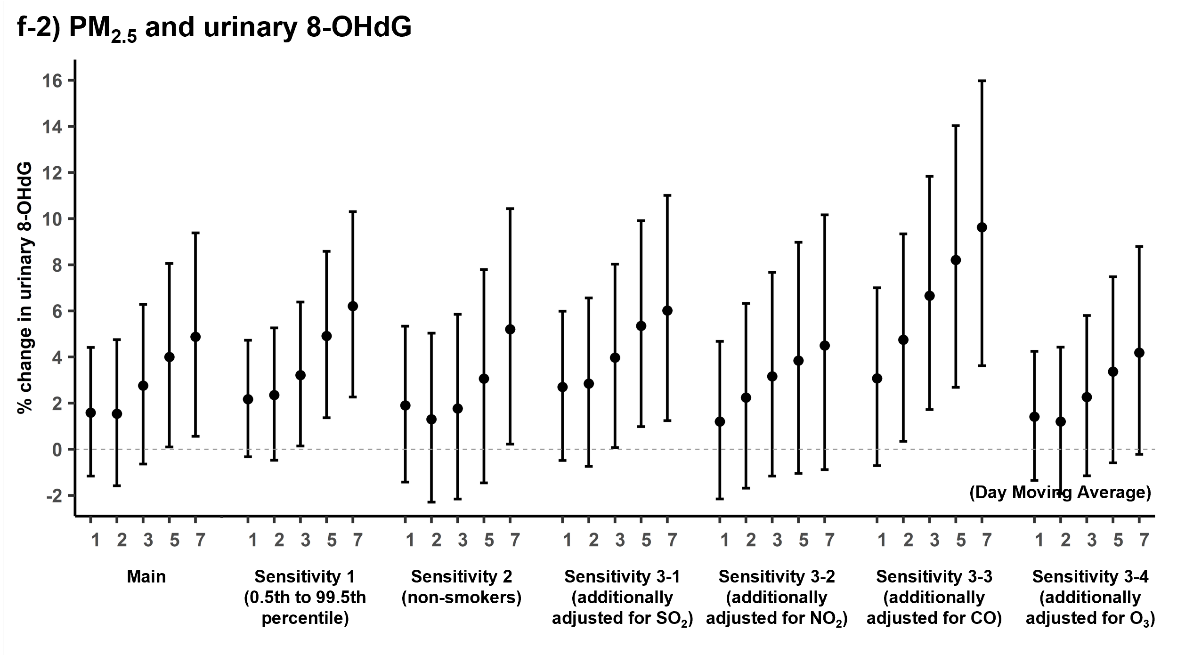 |
| --- | --- |
| 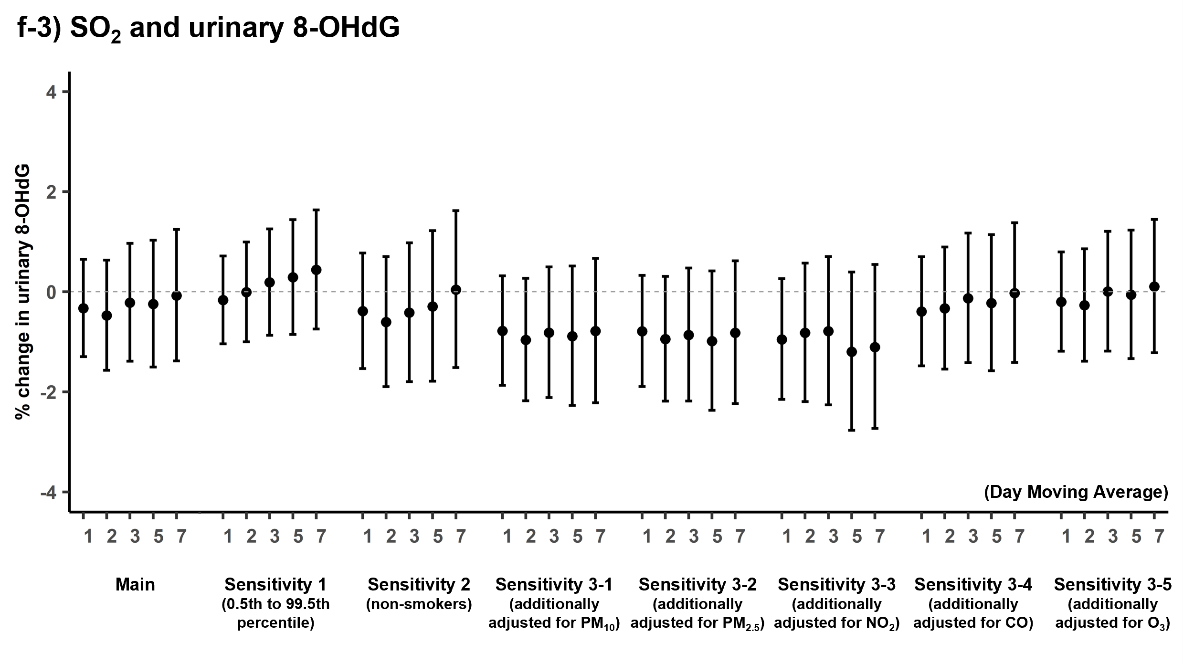 | 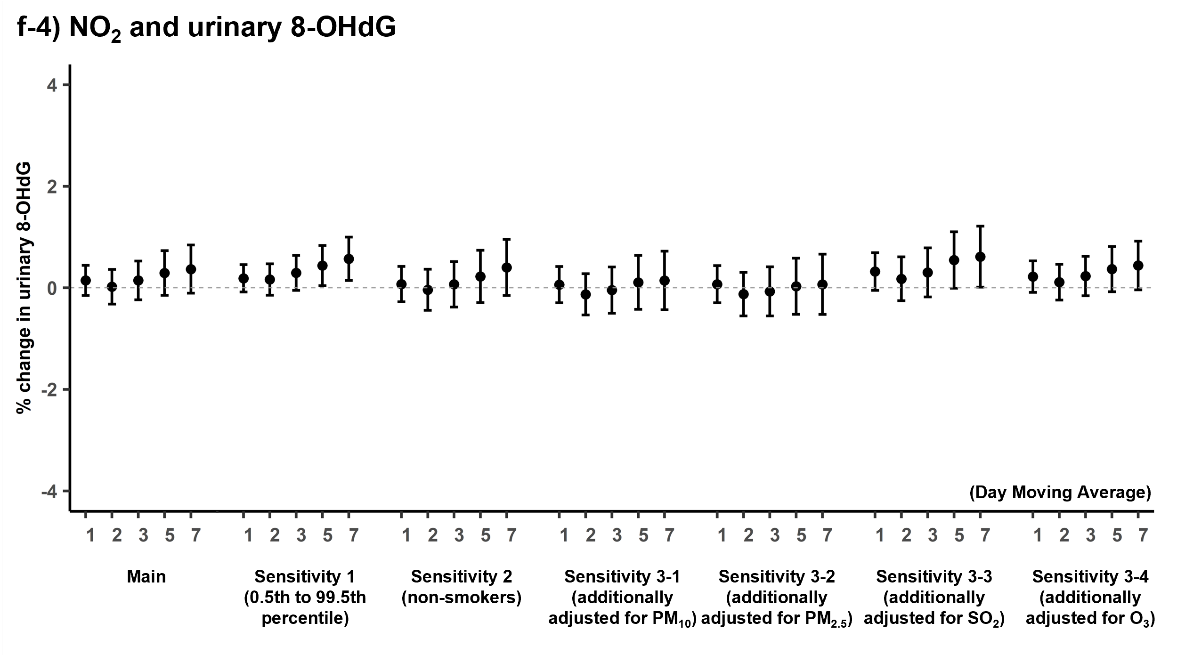 |

**Supplementary Figure 6.** Summary of main and sensitivity analyses on the associations between short-term exposure to air pollutants and 8-OHdG. The models are adjusted for date of examination as a linear term, continuous variables of age, body mass index, and the corresponding moving averages of temperature and relative humidity; and categorical variables of season, weekday of examination, residential region, sex, smoking history, alcohol consumption status, regular exercise, education, occupational status, and marital status. The sensitivity analysis on non-smokers did not account for smoking history; and two-pollutant model additionally controlled for the other air pollutant shown above. Estimates are presented as percentage changes with 95% confidence intervals in each biomarker level per 1-unit increase in 1- to 7-day average ambient air pollution exposure (units for PM: 10 μg/m^3^; SO_2_, NO_2_, and O_3_: 1 ppb; and CO: 0.1 ppm). PM_10_, PM_2.5_: particulate matter with aerodynamic diameter < 10 μm and < 2.5 μm, respectively; SO_2_: sulfur dioxide; NO_2_: nitrogen dioxide; CO: carbon monoxide; O_3_: ozone; 8-OHdG: 8-hydroxy-2′-deoxyguanosine.

| 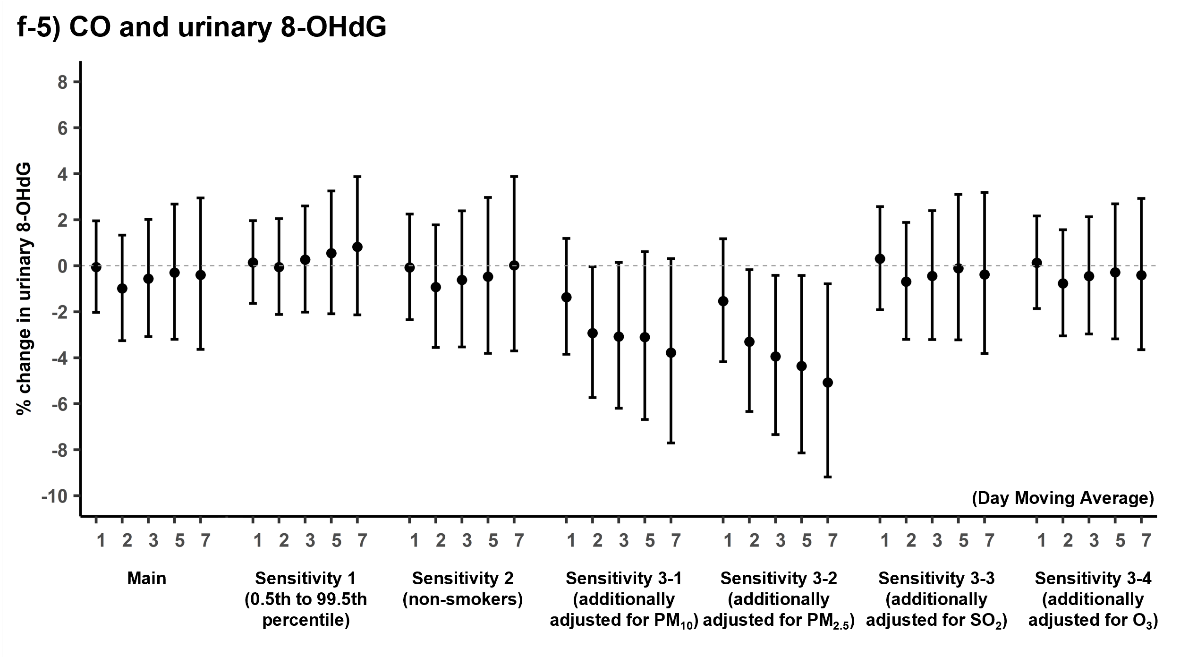 | 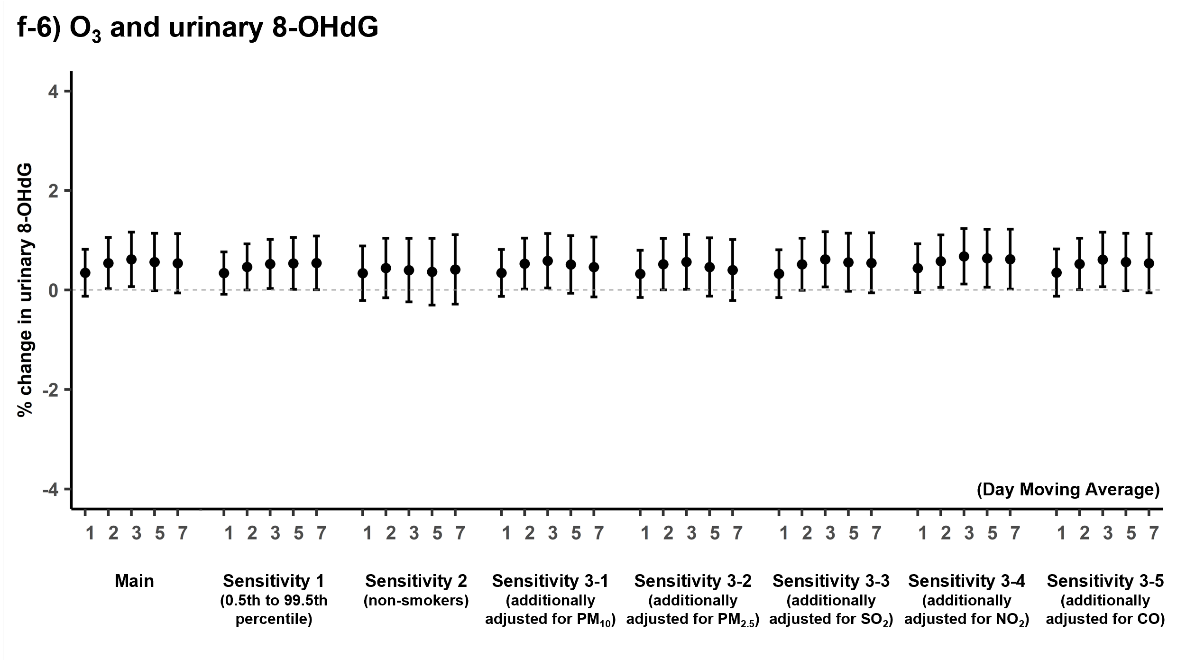 |
| --- | --- |

**Supplementary Figure 6.** Cont’d.

**Supplementary Table 1.** Associations between short-term exposure to air pollutants and biomarkers of inflammation and oxidative stress

| **Exposure window** | **Ambient air pollutants** | | | | | |
| --- | --- | --- | --- | --- | --- | --- |
| **(No. of observations)** | **PM_10_** | **PM_2.5_** | **SO_2_** | **NO_2_** | **CO** | **O_3_** |
| **Serum IL-1β (n=2199)** |  |  |  |  |  |  |
| 1-day average | **1.60 (0.33, 2.89)** | **3.65 (1.08, 6.28)** | 0.19 (–0.70, 1.09) | 0.10 (–0.17, 0.37) | 1.23 (–0.60, 3.09) | **0.59 (0.16, 1.02)** |
| 2-day average | **2.03 (0.62, 3.47)** | **5.23 (2.28, 8.26)** | 0.49 (–0.51, 1.51) | 0.25 (–0.06, 0.56) | 1.91 (–0.22, 4.08) | **0.62 (0.15, 1.09)** |
| 3-day average | **1.71 (0.20, 3.25)** | **4.84 (1.67, 8.10)** | 0.45 (–0.62, 1.53) | 0.28 (–0.06, 0.63) | 2.05 (–0.30, 4.46) | **0.71 (0.21, 1.21)** |
| 5-day average | 1.74 (–0.06, 3.57) | **5.09 (1.49, 8.81)** | 0.36 (–0.79, 1.53) | 0.27 (–0.13, 0.67) | 2.62 (–0.09, 5.41) | **0.70 (0.17, 1.22)** |
| 7-day average | **2.69 (0.62, 4.80)** | **6.38 (2.40, 10.52)** | 0.33 (–0.86, 1.53) | 0.27 (–0.16, 0.70) | **3.31 (0.26, 6.46)** | **0.74 (0.20, 1.28)** |
| **Serum IL-6 (n=2199)** |  |  |  |  |  |  |
| 1-day average | 0.18 (–0.98, 1.35) | 0.54 (–1.76, 2.89) | –0.14 (–0.95, 0.68) | 0.01 (–0.24, 0.27) | 0.43 (–1.24, 2.13) | –0.07 (–0.46, 0.33) |
| 2-day average | 0.08 (–1.20, 1.39) | 0.52 (–2.08, 3.20) | –0.09 (–1.01, 0.83) | 0.04 (–0.24, 0.33) | 0.50 (–1.43, 2.47) | –0.12 (–0.55, 0.31) |
| 3-day average | –0.19 (–1.56, 1.20) | –0.37 (–3.15, 2.49) | –0.31 (–1.28, 0.68) | –0.01 (–0.33, 0.31) | 0.13 (–1.99, 2.31) | –0.08 (–0.53, 0.38) |
| 5-day average | 0.03 (–1.60, 1.68) | 0.06 (–3.10, 3.33) | –0.48 (–1.53, 0.58) | –0.12 (–0.49, 0.25) | 0.63 (–1.82, 3.15) | –0.04 (–0.52, 0.45) |
| 7-day average | 0.29 (–1.57, 2.20) | 0.71 (–2.77, 4.32) | –0.70 (–1.79, 0.40) | –0.19 (–0.59, 0.20) | 1.04 (–1.72, 3.87) | 0.05 (–0.44, 0.55) |
| **Serum IL-8 (n=2199)** |  |  |  |  |  |  |
| 1-day average | 0.57 (–0.81, 1.97) | 1.86 (–0.89, 4.68) | –0.39 (–1.35, 0.58) | –0.02 (–0.31, 0.28) | 0.56 (–1.42, 2.58) | **1.02 (0.55, 1.50)** |
| 2-day average | 1.10 (–0.43, 2.66) | **3.17 (0.02, 6.43)** | –0.62 (–1.70, 0.47) | 0.04 (–0.30, 0.38) | 1.35 (–0.95, 3.71) | **1.15 (0.64, 1.66)** |
| 3-day average | 1.25 (–0.40, 2.92) | 3.22 (–0.18, 6.73) | –0.68 (–1.83, 0.49) | 0.10 (–0.27, 0.48) | 1.71 (–0.84, 4.33) | **1.24 (0.70, 1.79)** |
| 5-day average | 1.70 (–0.26, 3.69) | **4.90 (1.00, 8.96)** | –0.56 (–1.80, 0.70) | 0.28 (–0.16, 0.71) | 2.89 (–0.07, 5.94) | **1.11 (0.54, 1.69)** |
| 7-day average | **2.51 (0.26, 4.81)** | **6.32 (1.99, 10.84)** | –0.54 (–1.83, 0.76) | 0.27 (–0.20, 0.74) | 3.12 (–0.20, 6.56) | **1.27 (0.68, 1.86)** |
| **Serum IL-10 (n=2199)** |  |  |  |  |  |  |
| 1-day average | **2.15 (0.86, 3.46)** | **4.49 (1.88, 7.16)** | **0.94 (0.04, 1.85)** | 0.24 (–0.03, 0.51) | **2.00 (0.15, 3.89)** | –0.44 (–0.87, 0.00) |
| 2-day average | **2.34 (0.91, 3.80)** | **5.83 (2.84, 8.91)** | **1.09 (0.07, 2.12)** | 0.29 (–0.03, 0.60) | **2.53 (0.37, 4.73)** | –0.46 (–0.93, 0.01) |
| 3-day average | **1.87 (0.34, 3.42)** | **4.38 (1.20, 7.66)** | 0.93 (–0.16, 2.02) | 0.28 (–0.07, 0.63) | **2.61 (0.23, 5.05)** | **–0.55 (–1.05, –0.05)** |
| 5-day average | 1.53 (–0.28, 3.37) | 3.50 (–0.07, 7.20) | 0.38 (–0.78, 1.56) | 0.13 (–0.27, 0.53) | 2.68 (–0.05, 5.50) | –0.45 (–0.98, 0.07) |
| 7-day average | 1.03 (–1.03, 3.13) | 2.07 (–1.79, 6.08) | –0.14 (–1.34, 1.07) | –0.02 (–0.45, 0.41) | 1.76 (–1.28, 4.89) | –0.25 (–0.79, 0.29) |
| **Serum TNF-α (n=2199)** |  |  |  |  |  |  |
| 1-day average | 0.52 (–0.09, 1.14) | **1.40 (0.17, 2.64)** | 0.20 (–0.23, 0.64) | 0.06 (–0.07, 0.19) | 0.58 (–0.30, 1.47) | –0.13 (–0.34, 0.07) |
| 2-day average | **0.69 (0.01, 1.38)** | **1.81 (0.41, 3.22)** | 0.26 (–0.22, 0.75) | 0.09 (–0.06, 0.24) | 0.89 (–0.14, 1.93) | –0.10 (–0.32, 0.13) |
| 3-day average | 0.51 (–0.22, 1.25) | 1.37 (–0.13, 2.89) | 0.32 (–0.20, 0.84) | 0.11 (–0.06, 0.27) | 0.93 (–0.20, 2.08) | –0.08 (–0.32, 0.16) |
| 5-day average | 0.30 (–0.57, 1.17) | 0.99 (–0.70, 2.71) | 0.28 (–0.28, 0.84) | 0.15 (–0.04, 0.34) | 1.26 (–0.05, 2.58) | –0.18 (–0.43, 0.07) |
| 7-day average | 0.30 (–0.68, 1.30) | 0.99 (–0.86, 2.88) | 0.26 (–0.32, 0.84) | 0.15 (–0.05, 0.36) | **1.56 (0.10, 3.05)** | –0.16 (–0.42, 0.10) |
| **Urinary 8-OHdG (n=2138)** |  |  |  |  |  |  |
| 1-day average | 0.88 (–0.52, 2.31) | 1.59 (–1.16, 4.42) | –0.33 (–1.30, 0.65) | 0.15 (–0.15, 0.44) | –0.06 (–2.03, 1.95) | 0.35 (–0.12, 0.82) |
| 2-day average | 0.95 (–0.61, 2.52) | 1.54 (–1.58, 4.76) | –0.47 (–1.57, 0.63) | 0.02 (–0.32, 0.37) | –0.99 (–3.25, 1.33) | **0.54 (0.03, 1.05)** |
| 3-day average | 1.41 (–0.25, 3.10) | 2.76 (–0.64, 6.28) | –0.22 (–1.39, 0.97) | 0.15 (–0.23, 0.53) | –0.57 (–3.08, 2.01) | **0.62 (0.07, 1.16)** |
| 5-day average | 1.75 (–0.22, 3.76) | **4.00 (0.10, 8.06)** | –0.24 (–1.50, 1.03) | 0.29 (–0.15, 0.73) | –0.30 (–3.20, 2.68) | 0.56 (–0.01, 1.14) |
| 7-day average | 2.28 (–0.001, 4.61) | **4.88 (0.56, 9.38)** | –0.08 (–1.38, 1.25) | 0.37 (–0.11, 0.84) | –0.40 (–3.64, 2.94) | 0.54 (–0.06, 1.14) |

The models are adjusted for date of examination as a linear term, continuous variables of age, body mass index, and the corresponding moving averages of temperature and relative humidity; and categorical variables of season, weekday of examination, residential region, sex, smoking history, alcohol consumption status, regular exercise, education, occupation, and marital status. Estimates are presented as percentage changes with 95% confidence intervals in each biomarker level per 1-unit increase in 1- to 7-day average ambient air pollution exposure (units for PM: 10 µg/m^3^; SO_2_, NO_2_, and O_3_: 1 ppb; and CO: 0.1 ppm). PM_10_, PM_2.5:_ particulate matter with aerodynamic diameter < 10 µm and < 2.5 µm, respectively; SO_2_: sulfur dioxide; NO_2_: nitrogen dioxide; CO: carbon monoxide; O_3_: ozone; IL: interleukin; TNF: tumor necrosis factor; 8-OHdG: 8-hydroxy-2′-deoxyguanosine.

**Supplementary Table 2.** Summary statistics of the levels of biomarkers after excluding outliers

| **Biomarkers (unit)** | **No. of participants** | **Mean ± SD** | **Median** | **IQR** |
| --- | --- | --- | --- | --- |
| IL-1β (pg/mL) | 2172 | 1.26 ± 0.97 | 0.98 | 0.90 |
| IL-6 (pg/mL) | 2177 | 1.85 ± 1.23 | 1.53 | 1.23 |
| IL-8 (pg/mL) | 2178 | 21.73 ± 23.12 | 14.99 | 13.42 |
| IL-10 (pg/mL) | 2180 | 1.09 ± 0.72 | 0.89 | 0.82 |
| TNF-α (pg/mL) | 2177 | 12.54 ± 3.90 | 12.09 | 4.27 |
| 8-OHdG (μg/g creatinine) | 2117 | 5.04 ± 3.14 | 4.34 | 3.96 |

IL: interleukin; TNF: tumor necrosis factor; 8-OHdG: 8-hydroxy-2′-deoxyguanosine; IQR: interquartile range.

**Supplementary Table 3.** Associations between ambient air pollutants and biomarkers of inflammation and oxidative stress after excluding outliers

| **Exposure window** | **Ambient air pollutants** | | | | | |
| --- | --- | --- | --- | --- | --- | --- |
| **(No. of observations)** | **PM_10_** | **PM_2.5_** | **SO_2_** | **NO_2_** | **CO** | **O_3_** |
| **Serum IL-1β (n=2172)** |  |  |  |  |  |  |
| 1-day average | **1.39 (0.18, 2.60)** | **3.10 (0.68, 5.58)** | 0.09 (–0.75, 0.94) | 0.11 (–0.14, 0.37) | 1.33 (–0.40, 3.09) | **0.47 (0.07, 0.88)** |
| 2-day average | **1.85 (0.51, 3.21)** | **4.76 (1.98, 7.62)** | 0.43 (–0.51, 1.39) | 0.22 (–0.07, 0.52) | 1.93 (–0.08, 3.99) | **0.54 (0.10, 0.99)** |
| 3-day average | **1.77 (0.34, 3.23)** | **4.90 (1.89, 7.99)** | 0.48 (–0.53, 1.50) | 0.29 (–0.04, 0.62) | **2.38 (0.14, 4.66)** | **0.62 (0.15, 1.10)** |
| 5-day average | **2.04 (0.33, 3.78)** | **5.48 (2.06, 9.01)** | 0.44 (–0.65, 1.54) | 0.31 (–0.07, 0.69) | **3.26 (0.67, 5.92)** | **0.56 (0.07, 1.06)** |
| 7-day average | **2.62 (0.66, 4.62)** | **5.90 (2.13, 9.81)** | 0.37 (–0.75, 1.51) | 0.27 (–0.14, 0.68) | **3.60 (0.69, 6.59)** | **0.61 (0.09, 1.12)** |
| **Serum IL-6 (n=2177)** |  |  |  |  |  |  |
| 1-day average | 0.29 (–0.79, 1.38) | 0.87 (–1.27, 3.06) | 0.003 (–0.75, 0.77) | 0.11 (–0.12, 0.34) | 0.98 (–0.58, 2.57) | –0.07 (–0.44, 0.30) |
| 2-day average | 0.20 (–1.00, 1.40) | 1.02 (–1.41, 3.51) | 0.05 (–0.80, 0.91) | 0.15 (–0.12, 0.41) | 1.19 (–0.61, 3.03) | –0.06 (–0.46, 0.34) |
| 3-day average | –0.08 (–1.36, 1.21) | 0.35 (–2.25, 3.02) | –0.13 (–1.04, 0.79) | 0.10 (–0.19, 0.40) | 1.03 (–0.96, 3.07) | 0.02 (–0.40, 0.45) |
| 5-day average | 0.05 (–1.47, 1.59) | 0.69 (–2.26, 3.74) | –0.30 (–1.28, 0.69) | 0.01 (–0.34, 0.35) | 1.61 (–0.69, 3.97) | 0.05 (–0.40, 0.50) |
| 7-day average | –0.08 (–1.81, 1.68) | 0.51 (–2.73, 3.85) | –0.56 (–1.57, 0.46) | –0.09 (–0.46, 0.28) | 1.76 (–0.83, 4.41) | 0.10 (–0.36, 0.57) |
| **Serum IL-8 (n=2178)** |  |  |  |  |  |  |
| 1-day average | 0.32 (–0.99, 1.64) | 1.60 (–1.00, 4.27) | –0.34 (–1.25, 0.58) | 0.02 (–0.26, 0.30) | 0.49 (–1.38, 2.39) | **0.93 (0.48, 1.37)** |
| 2-day average | 0.77 (–0.69, 2.26) | 2.80 (–0.19, 5.88) | –0.57 (–1.59, 0.47) | 0.08 (–0.24, 0.40) | 1.40 (–0.78, 3.62) | **1.07 (0.59, 1.56)** |
| 3-day average | 1.00 (–0.57, 2.59) | **3.28 (0.05, 6.61)** | –0.57 (–1.66, 0.53) | 0.15 (–0.21, 0.51) | 1.75 (–0.66, 4.22) | **1.15 (0.63, 1.66)** |
| 5-day average | 1.42 (–0.43, 3.31) | **4.77 (1.07, 8.60)** | –0.51 (–1.69, 0.68) | 0.27 (–0.15, 0.68) | 2.61 (–0.18, 5.47) | **1.00 (0.46, 1.54)** |
| 7-day average | 1.95 (–0.17, 4.12) | **5.43 (1.36, 9.67)** | –0.55 (–1.77, 0.68) | 0.23 (–0.21, 0.67) | 2.64 (–0.48, 5.86) | **1.10 (0.54, 1.66)** |
| **Serum IL-10 (n=2180)** |  |  |  |  |  |  |
| 1-day average | **1.96 (0.76, 3.17)** | **3.88 (1.47, 6.35)** | 0.78 (–0.06, 1.63) | 0.23 (–0.03, 0.48) | **1.90 (0.17, 3.66)** | **–0.59 (–0.99, –0.19)** |
| 2-day average | **2.09 (0.76, 3.44)** | **5.08 (2.31, 7.93)** | **1.02 (0.07, 1.97)** | 0.27 (–0.02, 0.57) | **2.24 (0.24, 4.28)** | **–0.59 (–1.03, –0.16)** |
| 3-day average | **1.56 (0.13, 3.01)** | **3.75 (0.80, 6.79)** | 0.88 (–0.13, 1.90) | 0.26 (–0.06, 0.59) | **2.33 (0.12, 4.59)** | **–0.65 (–1.12, –0.19)** |
| 5-day average | 1.26 (–0.46, 3.00) | **3.44 (0.10, 6.89)** | 0.51 (–0.57, 1.61) | 0.17 (–0.21, 0.54) | **3.00 (0.45, 5.62)** | **–0.54 (–1.03, –0.05)** |
| 7-day average | 1.11 (–0.83, 3.09) | 2.73 (–0.90, 6.49) | 0.16 (–0.96, 1.29) | 0.03 (–0.37, 0.43) | 2.61 (–0.25, 5.54) | –0.38 (–0.89, 0.12) |
| **Serum TNF-α (n=2177)** |  |  |  |  |  |  |
| 1-day average | 0.45 (–0.12, 1.02) | **1.28 (0.14, 2.43)** | 0.18 (–0.22, 0.58) | 0.03 (–0.09, 0.16) | 0.52 (–0.30, 1.35) | –0.06 (–0.25, 0.14) |
| 2-day average | **0.66 (0.03, 1.30)** | **1.75 (0.45, 3.06)** | 0.24 (–0.21, 0.70) | 0.07 (–0.07, 0.22) | **0.97 (0.01, 1.93)** | –0.04 (–0.25, 0.17) |
| 3-day average | 0.53 (–0.15, 1.21) | 1.39 (–0.01, 2.80) | 0.27 (–0.21, 0.76) | 0.09 (–0.07, 0.24) | 1.04 (–0.01, 2.11) | –0.03 (–0.25, 0.20) |
| 5-day average | 0.36 (–0.44, 1.17) | 1.20 (–0.37, 2.80) | 0.24 (–0.28, 0.76) | 0.09 (–0.09, 0.27) | **1.26 (0.05, 2.49)** | –0.10 (–0.34, 0.14) |
| 7-day average | 0.38 (–0.54, 1.30) | 1.19 (–0.54, 2.95) | 0.22 (–0.32, 0.76) | 0.10 (–0.10, 0.29) | **1.52 (0.16, 2.90)** | –0.07 (–0.31, 0.17) |
| **Urinary 8-OHdG (n=2117)** |  |  |  |  |  |  |
| 1-day average | 1.15 (–0.12, 2.43) | 2.17 (–0.32, 4.73) | –0.17 (–1.04, 0.72) | 0.19 (–0.08, 0.46) | 0.14 (–1.64, 1.96) | 0.34 (–0.08, 0.77) |
| 2-day average | 1.33 (–0.08, 2.75) | 2.35 (–0.48, 5.27) | –0.01 (–1.00, 1.00) | 0.16 (–0.14, 0.47) | –0.06 (–2.12, 2.05) | **0.46 (0.001, 0.93)** |
| 3-day average | **1.66 (0.16, 3.18)** | **3.22 (0.14, 6.39)** | 0.19 (–0.87, 1.26) | 0.30 (–0.05, 0.64) | 0.26 (–2.02, 2.60) | **0.52 (0.03, 1.02)** |
| 5-day average | **2.26 (0.47, 4.07)** | **4.91 (1.37, 8.59)** | 0.29 (–0.85, 1.44) | **0.44 (0.04, 0.84)** | 0.54 (–2.09, 3.25) | **0.53 (0.02, 1.06)** |
| 7-day average | **3.09 (1.02, 5.20)** | **6.21 (2.26, 10.30)** | 0.44 (–0.74, 1.63) | **0.57 (0.14, 1.00)** | 0.82 (–2.14, 3.87) | **0.54 (0.01, 1.08)** |

The models are adjusted for date of examination as a linear term, continuous variables of age, body mass index, and the corresponding moving averages of temperature and relative humidity; and categorical variables of season, weekday of examination, residential region, sex, smoking history, alcohol consumption status, regular exercise, education, occupation, and marital status. Estimates are presented as percentage changes with 95% confidence intervals in each biomarker level per 1-unit increase in 1- to 7-day average ambient air pollution exposure (units for PM: 10 µg/m^3^; SO_2_, NO_2_, and O_3_: 1 ppb; and CO: 0.1 ppm). PM_10_, PM_2.5:_ particulate matter with aerodynamic diameter < 10 µm and < 2.5 µm, respectively; SO_2_: sulfur dioxide; NO_2_: nitrogen dioxide; CO: carbon monoxide; O_3_: ozone; IL: interleukin; TNF: tumor necrosis factor; 8-OHdG: 8-hydroxy-2′-deoxyguanosine.

**Supplementary Table 4.** Associations between ambient air pollutants and biomarkers of inflammation and oxidative stress among non-smokers

| **Exposure window** | **Ambient air pollutants** | | | | | |
| --- | --- | --- | --- | --- | --- | --- |
| **(No. of observations)** | **PM_10_** | **PM_2.5_** | **SO_2_** | **NO_2_** | **CO** | **O_3_** |
| **Serum IL-1β (n=1527)** |  |  |  |  |  |  |
| 1-day average | **2.25 (0.59, 3.94)** | **4.44 (1.27, 7.70)** | –0.47 (–1.53, 0.60) | –0.02 (–0.34, 0.30) | 1.38 (–0.76, 3.57) | **0.69 (0.18, 1.20)** |
| 2-day average | **2.60 (0.86, 4.36)** | **5.72 (2.25, 9.32)** | –0.08 (–1.27, 1.13) | 0.15 (–0.22, 0.53) | 2.15 (–0.36, 4.72) | **0.68 (0.13, 1.23)** |
| 3-day average | **2.19 (0.39, 4.03)** | **5.35 (1.59, 9.26)** | –0.10 (–1.38, 1.18) | 0.22 (–0.19, 0.64) | 2.58 (–0.20, 5.45) | **0.73 (0.14, 1.32)** |
| 5-day average | **2.65 (0.49, 4.86)** | **6.91 (2.58, 11.42)** | –0.10 (–1.48, 1.30) | 0.24 (–0.23, 0.72) | **3.69 (0.49, 6.99)** | **0.68 (0.06, 1.30)** |
| 7-day average | **3.95 (1.48, 6.48)** | **8.72 (3.98, 13.68)** | 0.01 (–1.42, 1.46) | 0.33 (–0.17, 0.84) | **5.07 (1.47, 8.80)** | **0.69 (0.04, 1.33)** |
| **Serum IL-6 (n=1527)** |  |  |  |  |  |  |
| 1-day average | 0.86 (–0.68, 2.41) | 1.55 (–1.33, 4.52) | –0.22 (–1.21, 0.79) | 0.03 (–0.28, 0.33) | 1.32 (–0.67, 3.36) | –0.04 (–0.51, 0.44) |
| 2-day average | 0.71 (–0.88, 2.34) | 1.25 (–1.87, 4.47) | –0.12 (–1.23, 1.01) | 0.12 (–0.23, 0.47) | 1.60 (–0.73, 3.98) | –0.13 (–0.65, 0.38) |
| 3-day average | 0.21 (–1.44, 1.90) | –0.13 (–3.48, 3.33) | –0.43 (–1.61, 0.77) | 0.08 (–0.30, 0.47) | 1.01 (–1.55, 3.64) | –0.20 (–0.75, 0.35) |
| 5-day average | 0.40 (–1.58, 2.42) | 0.41 (–3.41, 4.37) | –0.63 (–1.91, 0.66) | –0.05 (–0.50, 0.39) | 1.40 (–1.53, 4.42) | –0.20 (–0.77, 0.38) |
| 7-day average | 0.79 (–1.46, 3.08) | 1.31 (–2.84, 5.63) | –0.81 (–2.14, 0.53) | –0.12 (–0.60, 0.35) | 1.93 (–1.35, 5.31) | –0.15 (–0.74, 0.45) |
| **Serum IL-8 (n=1527)** |  |  |  |  |  |  |
| 1-day average | 0.93 (–0.88, 2.78) | 2.69 (–0.75, 6.25) | –0.22 (–1.40, 0.97) | –0.06 (–0.42, 0.30) | 0.77 (–1.58, 3.18) | **1.06 (0.49, 1.62)** |
| 2-day average | 1.84 (–0.07, 3.78) | **4.28 (0.49, 8.22)** | –0.38 (–1.69, 0.96) | 0.04 (–0.37, 0.45) | 1.62 (–1.13, 4.45) | **1.05 (0.44, 1.66)** |
| 3-day average | **2.11 (0.12, 4.14)** | **4.67 (0.54, 8.97)** | –0.39 (–1.79, 1.03) | 0.15 (–0.31, 0.61) | 2.21 (–0.85, 5.37) | **1.10 (0.45, 1.76)** |
| 5-day average | **2.72 (0.33, 5.16)** | **6.60 (1.83, 11.58)** | –0.27 (–1.78, 1.28) | 0.34 (–0.18, 0.87) | 3.18 (–0.34, 6.82) | **0.96 (0.28, 1.65)** |
| 7-day average | **3.79 (1.06, 6.59)** | **8.46 (3.24, 13.94)** | –0.05 (–1.63, 1.56) | 0.42 (–0.14, 0.98) | 3.82 (–0.12, 7.91) | **1.07 (0.36, 1.79)** |
| **Serum IL-10 (n=1527)** |  |  |  |  |  |  |
| 1-day average | **2.93 (1.24, 4.65)** | **5.20 (1.97, 8.53)** | 0.88 (–0.22, 1.98) | 0.24 (–0.09, 0.57) | 2.08 (–0.10, 4.32) | **–0.56 (–1.07, –0.05)** |
| 2-day average | **3.08 (1.31, 4.88)** | **6.48 (2.93, 10.15)** | **1.53 (0.30, 2.77)** | **0.42 (0.04, 0.80)** | **3.24 (0.68, 5.88)** | **–0.56 (–1.12, –0.01)** |
| 3-day average | **2.46 (0.63, 4.33)** | **5.07 (1.25, 9.03)** | **1.47 (0.17, 2.80)** | **0.45 (0.03, 0.88)** | **3.64 (0.79, 6.58)** | **–0.76 (–1.35, –0.17)** |
| 5-day average | **2.27 (0.08, 4.51)** | **4.41 (0.11, 8.89)** | 0.97 (–0.44, 2.40) | 0.33 (–0.15, 0.81) | **3.82 (0.57, 7.18)** | **–0.69 (–1.31, –0.06)** |
| 7-day average | 1.80 (–0.66, 4.32) | 2.86 (–1.71, 7.63) | 0.32 (–1.13, 1.80) | 0.17 (–0.35, 0.68) | 2.69 (–0.89, 6.40) | –0.53 (–1.17, 0.12) |
| **Serum TNF-α (n=1527)** |  |  |  |  |  |  |
| 1-day average | 0.63 (–0.17, 1.44) | 1.46 (–0.04, 2.99) | 0.10 (–0.42, 0.62) | 0.04 (–0.11, 0.20) | 0.62 (–0.41, 1.67) | –0.14 (–0.39, 0.11) |
| 2-day average | 0.81 (–0.03, 1.65) | **1.94 (0.29, 3.61)** | 0.18 (–0.40, 0.77) | 0.10 (–0.08, 0.28) | 1.07 (–0.15, 2.29) | –0.11 (–0.38, 0.15) |
| 3-day average | 0.43 (–0.44, 1.31) | 1.12 (–0.65, 2.93) | 0.18 (–0.44, 0.80) | 0.10 (–0.11, 0.30) | 0.98 (–0.37, 2.33) | –0.12 (–0.41, 0.16) |
| 5-day average | 0.17 (–0.86, 1.21) | 0.80 (–1.21, 2.85) | 0.15 (–0.52, 0.83) | 0.13 (–0.10, 0.36) | 1.12 (–0.41, 2.68) | –0.23 (–0.52, 0.07) |
| 7-day average | 0.17 (–1.00, 1.35) | 0.82 (–1.34, 3.04) | 0.16 (–0.54, 0.86) | 0.15 (–0.09, 0.40) | 1.38 (–0.33, 3.11) | –0.21 (–0.52, 0.10) |
| **Urinary 8-OHdG (n=1483)** |  |  |  |  |  |  |
| 1-day average | 0.98 (–0.80, 2.79) | 1.90 (–1.42, 5.34) | –0.39 (–1.53, 0.78) | 0.07 (–0.28, 0.42) | –0.08 (–2.35, 2.24) | 0.34 (–0.21, 0.89) |
| 2-day average | 0.78 (–1.07, 2.65) | 1.30 (–2.30, 5.03) | –0.60 (–1.89, 0.70) | –0.04 (–0.44, 0.37) | –0.93 (–3.55, 1.78) | 0.44 (–0.15, 1.04) |
| 3-day average | 1.06 (–0.87, 3.02) | 1.77 (–2.16, 5.85) | –0.42 (–1.79, 0.98) | 0.07 (–0.38, 0.52) | –0.62 (–3.53, 2.39) | 0.40 (–0.24, 1.04) |
| 5-day average | 1.30 (–1.01, 3.67) | 3.06 (–1.46, 7.80) | –0.29 (–1.79, 1.22) | 0.22 (–0.29, 0.74) | –0.48 (–3.81, 2.97) | 0.37 (–0.30, 1.04) |
| 7-day average | 2.33 (–0.32, 5.05) | **5.21 (0.22, 10.43)** | 0.04 (–1.51, 1.62) | 0.40 (–0.15, 0.96) | 0.01 (–3.70, 3.88) | 0.41 (–0.28, 1.11) |

The models are adjusted for date of examination as a linear term, continuous variables of age, body mass index, and the corresponding moving averages of temperature and relative humidity; and categorical variables of season, weekday of examination, residential region, sex, alcohol consumption status, regular exercise, education, occupation, and marital status. Estimates are presented as percentage changes with 95% confidence intervals in each biomarker level per 1-unit increase in 1- to 7-day average ambient air pollution exposure (units for PM: 10 µg/m^3^; SO_2_, NO_2_, and O_3_: 1 ppb; and CO: 0.1 ppm). PM_10_, PM_2.5:_ particulate matter with aerodynamic diameter < 10 µm and < 2.5 µm, respectively; SO_2_: sulfur dioxide; NO_2_: nitrogen dioxide; CO: carbon monoxide; O_3_: ozone; IL: interleukin; TNF: tumor necrosis factor; 8-OHdG: 8-hydroxy-2′-deoxyguanosine.

**Supplementary Table 5-1.** Associations between ambient air pollutants and IL-1β in the two-pollutant model

| **Exposure window** | **Ambient air pollutants** | | | | | |
| --- | --- | --- | --- | --- | --- | --- |
| **(No. of observations)** | **PM_10_** | **PM_2.5_** | **SO_2_** | **NO_2_** | **CO** | **O_3_** |
| **Serum IL-1β (n=2199)** |  |  |  |  |  |  |
|  | **With SO_2_** | **With SO_2_** | **With PM_10_** | **With PM_10_** | **With PM_10_** | **With PM_10_** |
| 1-day average | **1.88 (0.44, 3.34)** | **4.48 (1.51, 7.53)** | –0.42 (–1.41, 0.59) | –0.13 (–0.45, 0.20) | –0.31 (–2.60, 2.03) | **0.58 (0.15, 1.01)** |
| 2-day average | **2.15 (0.57, 3.76)** | **5.92 (2.54, 9.41)** | –0.18 (–1.29, 0.94) | 0.01 (–0.36, 0.38) | 0.06 (–2.56, 2.76) | **0.60 (0.14, 1.07)** |
| 3-day average | **1.78 (0.09, 3.50)** | **5.46 (1.84, 9.21)** | –0.11 (–1.29, 1.09) | 0.10 (–0.32, 0.52) | 0.64 (–2.31, 3.68) | **0.68 (0.18, 1.18)** |
| 5-day average | 1.84 (–0.15, 3.87) | **5.68 (1.68, 9.85)** | –0.15 (–1.42, 1.14) | 0.08 (–0.41, 0.56) | 1.61 (–1.82, 5.15) | **0.65 (0.12, 1.18)** |
| 7-day average | **3.01 (0.71, 5.36)** | **7.17 (2.77, 11.74)** | –0.42 (–1.73, 0.90) | –0.08 (–0.61, 0.44) | 1.41 (–2.35, 5.32) | **0.65 (0.11, 1.20)** |
|  | **With NO_2_** | **With NO_2_** | **With PM_2.5_** | **With PM_2.5_** | **With PM_2.5_** | **With PM_2.5_** |
| 1-day average | **1.92 (0.40, 3.47)** | **4.73 (1.56, 8.01)** | –0.57 (–1.58, 0.45) | –0.19 (–0.53, 0.14) | –0.99 (–3.41, 1.49) | **0.53 (0.10, 0.96)** |
| 2-day average | **2.02 (0.33, 3.73)** | **6.05 (2.33, 9.92)** | –0.48 (–1.61, 0.66) | –0.14 (–0.53, 0.25) | –1.34 (–4.16, 1.57) | **0.54 (0.07, 1.01)** |
| 3-day average | 1.48 (–0.34, 3.32) | **5.27 (1.23, 9.47)** | –0.43 (–1.63, 0.79) | –0.07 (–0.52, 0.37) | –0.98 (–4.18, 2.32) | **0.62 (0.12, 1.12)** |
| 5-day average | 1.54 (–0.63, 3.76) | **5.78 (1.22, 10.55)** | –0.43 (–1.70, 0.85) | –0.12 (–0.63, 0.38) | –0.04 (–3.65, 3.70) | **0.57 (0.04, 1.11)** |
| 7-day average | **2.92 (0.38, 5.52)** | **7.82 (2.75, 13.13)** | –0.56 (–1.85, 0.75) | –0.25 (–0.78, 0.29) | 0.04 (–3.90, 4.14) | **0.57 (0.01, 1.12)** |
|  | **With CO** | **With CO** | **With NO_2_** | **With SO_2_** | **With SO_2_** | **With SO_2_** |
| 1-day average | **1.74 (0.11, 3.39)** | **4.62 (1.11, 8.24)** | –0.001 (–1.11, 1.12) | 0.10 (–0.24, 0.44) | 1.33 (–0.72, 3.42) | **0.62 (0.19, 1.06)** |
| 2-day average | **2.01 (0.22, 3.83)** | **6.55 (2.45, 10.82)** | 0.01 (–1.24, 1.29) | 0.25 (–0.15, 0.64) | 1.79 (–0.56, 4.20) | **0.69 (0.21, 1.16)** |
| 3-day average | 1.45 (–0.47, 3.42) | **5.80 (1.32, 10.47)** | –0.14 (–1.48, 1.22) | 0.31 (–0.13, 0.75) | 1.98 (–0.61, 4.63) | **0.77 (0.26, 1.28)** |
| 5-day average | 1.07 (–1.21, 3.40) | **5.12 (0.22, 10.27)** | –0.19 (–1.63, 1.28) | 0.31 (–0.19, 0.82) | 2.68 (–0.24, 5.68) | **0.74 (0.21, 1.27)** |
| 7-day average | 2.09 (–0.50, 4.75) | **6.34 (1.04, 11.92)** | –0.20 (–1.70, 1.31) | 0.31 (–0.23, 0.86) | **3.43 (0.18, 6.80)** | **0.78 (0.23, 1.33)** |
|  | **With O_3_** | **With O_3_** | **With CO** | **With O_3_** | **With O_3_** | **With NO_2_** |
| 1-day average | **1.58 (0.31, 2.87)** | **3.34 (0.78, 5.98)** | –0.10 (–1.09, 0.90) | 0.21 (–0.07, 0.49) | 1.57 (–0.27, 3.45) | **0.67 (0.23, 1.12)** |
| 2-day average | **1.98 (0.56, 3.42)** | **4.86 (1.91, 7.90)** | 0.13 (–0.98, 1.25) | **0.36 (0.04, 0.68)** | **2.19 (0.05, 4.37)** | **0.75 (0.27, 1.23)** |
| 3-day average | **1.58 (0.07, 3.12)** | **4.29 (1.11, 7.57)** | 0.07 (–1.10, 1.26) | **0.39 (0.03, 0.74)** | 2.19 (–0.16, 4.60) | **0.81 (0.30, 1.32)** |
| 5-day average | 1.50 (–0.30, 3.33) | **4.30 (0.67, 8.07)** | –0.06 (–1.29, 1.19) | 0.36 (–0.04, 0.77) | 2.65 (–0.06, 5.43) | **0.77 (0.24, 1.30)** |
| 7-day average | **2.34 (0.26, 4.46)** | **5.41 (1.36, 9.62)** | –0.13 (–1.39, 1.14) | 0.36 (–0.07, 0.80) | **3.31 (0.26, 6.45)** | **0.81 (0.26, 1.36)** |
|  |  |  | **With O_3_** |  |  | **With CO** |
| 1-day average | - | - | 0.43 (–0.47, 1.34) | - | - | **0.63 (0.20, 1.07)** |
| 2-day average | - | - | 0.77 (–0.26, 1.80) | - | - | **0.67 (0.20, 1.14)** |
| 3-day average | - | - | 0.73 (–0.35, 1.83) | - | - | **0.73 (0.23, 1.23)** |
| 5-day average | - | - | 0.61 (–0.55, 1.79) | - | - | **0.70 (0.18, 1.22)** |
| 7-day average | - | - | 0.60 (–0.61, 1.82) | - | - | **0.74 (0.20, 1.28)** |

The models are adjusted for date of examination as a linear term, continuous variables of age, body mass index, corresponding moving averages of temperature and relative humidity, and the co-pollutant shown above; and categorical variables of season, weekday of examination, residential region, sex, smoking history, alcohol consumption status, regular exercise, education, occupation, and marital status.

Estimates are presented as percentage changes with 95% confidence intervals in each biomarker level per 1-unit increase in 1- to 7-day average ambient air pollution exposure (units for PM: 10 µg/m^3^; SO_2_, NO_2_, and O_3_: 1 ppb; and CO: 0.1 ppm). PM_10_, PM_2.5:_ particulate matter with aerodynamic diameter < 10 µm and < 2.5 µm, respectively; SO_2_: sulfur dioxide; NO_2_: nitrogen dioxide; CO: carbon monoxide; O_3_: ozone; IL: interleukin.

**Supplementary Table 5-2.** Associations between ambient air pollutants and IL-6 in the two-pollutant model

| **Exposure window** | **Ambient air pollutants** | | | | | |
| --- | --- | --- | --- | --- | --- | --- |
| **(No. of observations)** | **PM_10_** | **PM_2.5_** | **SO_2_** | **NO_2_** | **CO** | **O_3_** |
| **Serum IL-6 (n=2199)** |  |  |  |  |  |  |
|  | **With SO_2_** | **With SO_2_** | **With PM_10_** | **With PM_10_** | **With PM_10_** | **With PM_10_** |
| 1-day average | 0.34 (–0.97, 1.67) | 0.97 (–1.68, 3.68) | –0.25 (–1.17, 0.68) | –0.01 (–0.31, 0.29) | 0.44 (–1.68, 2.61) | –0.07 (–0.46, 0.33) |
| 2-day average | 0.18 (–1.26, 1.63) | 0.85 (–2.13, 3.91) | –0.15 (–1.18, 0.89) | 0.05 (–0.30, 0.39) | 0.67 (–1.77, 3.17) | –0.12 (–0.55, 0.31) |
| 3-day average | 0.01 (–1.53, 1.56) | 0.06 (–3.11, 3.34) | –0.31 (–1.40, 0.79) | 0.02 (–0.36, 0.40) | 0.51 (–2.21, 3.31) | –0.07 (–0.53, 0.39) |
| 5-day average | 0.43 (–1.38, 2.28) | 0.86 (–2.67, 4.52) | –0.60 (–1.77, 0.58) | –0.18 (–0.63, 0.26) | 1.00 (–2.14, 4.24) | –0.04 (–0.52, 0.45) |
| 7-day average | 1.00 (–1.08, 3.13) | 1.99 (–1.88, 6.00) | –0.95 (–2.15, 0.26) | –0.34 (–0.82, 0.14) | 1.23 (–2.24, 4.83) | 0.04 (–0.46, 0.55) |
|  | **With NO_2_** | **With NO_2_** | **With PM_2.5_** | **With PM_2.5_** | **With PM_2.5_** | **With PM_2.5_** |
| 1-day average | 0.20 (–1.19, 1.60) | 0.69 (–2.13, 3.60) | –0.31 (–1.24, 0.64) | –0.03 (–0.34, 0.28) | 0.30 (–1.96, 2.62) | –0.08 (–0.47, 0.32) |
| 2-day average | –0.03 (–1.55, 1.52) | 0.45 (–2.81, 3.83) | –0.24 (–1.28, 0.82) | 0.01 (–0.35, 0.37) | 0.44 (–2.21, 3.17) | –0.13 (–0.56, 0.30) |
| 3-day average | –0.23 (–1.87, 1.44) | –0.51 (–4.03, 3.15) | –0.32 (–1.43, 0.81) | 0.02 (–0.38, 0.43) | 0.67 (–2.34, 3.76) | –0.07 (–0.53, 0.39) |
| 5-day average | 0.49 (–1.49, 2.52) | 1.15 (–2.88, 5.34) | –0.61 (–1.77, 0.57) | –0.20 (–0.66, 0.26) | 1.14 (–2.24, 4.62) | –0.04 (–0.53, 0.45) |
| 7-day average | 1.24 (–1.06, 3.60) | 2.83 (–1.64, 7.50) | –0.95 (–2.14, 0.25) | –0.38 (–0.88, 0.12) | 1.19 (–2.49, 5.02) | 0.03 (–0.48, 0.55) |
|  | **With CO** | **With CO** | **With NO_2_** | **With SO_2_** | **With SO_2_** | **With SO_2_** |
| 1-day average | –0.01 (–1.49, 1.48) | 0.25 (–2.85, 3.46) | –0.26 (–1.28, 0.77) | 0.06 (–0.25, 0.38) | 0.70 (–1.18, 2.61) | –0.08 (–0.49, 0.32) |
| 2-day average | –0.19 (–1.80, 1.46) | 0.11 (–3.45, 3.80) | –0.28 (–1.44, 0.89) | 0.10 (–0.27, 0.46) | 0.72 (–1.43, 2.91) | –0.14 (–0.57, 0.30) |
| 3-day average | –0.39 (–2.14, 1.39) | –0.99 (–4.86, 3.05) | –0.46 (–1.69, 0.79) | 0.08 (–0.32, 0.49) | 0.51 (–1.84, 2.91) | –0.10 (–0.57, 0.36) |
| 5-day average | –0.38 (–2.46, 1.73) | –0.94 (–5.21, 3.53) | –0.43 (–1.75, 0.92) | –0.03 (–0.49, 0.43) | 1.22 (–1.43, 3.95) | –0.07 (–0.56, 0.42) |
| 7-day average | –0.21 (–2.55, 2.18) | –0.29 (–4.89, 4.52) | –0.60 (–1.97, 0.79) | –0.06 (–0.56, 0.44) | 1.88 (–1.08, 4.93) | 0.005 (–0.50, 0.51) |
|  | **With O_3_** | **With O_3_** | **With CO** | **With O_3_** | **With O_3_** | **With NO_2_** |
| 1-day average | 0.18 (–0.98, 1.35) | 0.58 (–1.73, 2.94) | –0.29 (–1.21, 0.63) | 0.003 (–0.26, 0.26) | 0.40 (–1.29, 2.11) | –0.07 (–0.48, 0.34) |
| 2-day average | 0.10 (–1.19, 1.40) | 0.61 (–2.01, 3.30) | –0.24 (–1.26, 0.79) | 0.02 (–0.27, 0.32) | 0.45 (–1.49, 2.43) | –0.12 (–0.55, 0.33) |
| 3-day average | –0.17 (–1.55, 1.22) | –0.32 (–3.13, 2.58) | –0.40 (–1.48, 0.68) | –0.02 (–0.35, 0.30) | 0.12 (–2.01, 2.30) | –0.08 (–0.54, 0.38) |
| 5-day average | 0.04 (–1.60, 1.71) | 0.11 (–3.11, 3.45) | –0.67 (–1.80, 0.47) | –0.13 (–0.50, 0.24) | 0.63 (–1.82, 3.15) | –0.06 (–0.55, 0.42) |
| 7-day average | 0.27 (–1.61, 2.19) | 0.66 (–2.92, 4.37) | –0.95 (–2.11, 0.21) | –0.19 (–0.59, 0.21) | 1.04 (–1.72, 3.87) | 0.02 (–0.48, 0.52) |
|  |  |  | **With O_3_** |  |  | **With CO** |
| 1-day average | - | - | –0.17 (–1.00, 0.67) | - | - | –0.06 (–0.45, 0.34) |
| 2-day average | - | - | –0.15 (–1.08, 0.80) | - | - | –0.11 (–0.54, 0.32) |
| 3-day average | - | - | –0.34 (–1.34, 0.66) | - | - | –0.07 (–0.53, 0.38) |
| 5-day average | - | - | –0.51 (–1.57, 0.57) | - | - | –0.04 (–0.51, 0.45) |
| 7-day average | - | - | –0.70 (–1.80, 0.41) | - | - | 0.05 (–0.44, 0.55) |

The models are adjusted for date of examination as a linear term, continuous variables of age, body mass index, corresponding moving averages of temperature and relative humidity, and the co-pollutant shown above; and categorical variables of season, weekday of examination, residential region, sex, smoking history, alcohol consumption status, regular exercise, education, occupation, and marital status. Estimates are presented as percentage changes with 95% confidence intervals in each biomarker level per 1-unit increase in 1- to 7-day average ambient air pollution exposure (units for PM: 10 µg/m^3^; SO_2_, NO_2_, and O_3_: 1 ppb; and CO: 0.1 ppm). PM_10_, PM_2.5:_ particulate matter with aerodynamic diameter < 10 µm and < 2.5 µm, respectively; SO_2_: sulfur dioxide; NO_2_: nitrogen dioxide; CO: carbon monoxide; O_3_: ozone; IL: interleukin.

**Supplementary Table 5-3.** Associations between ambient air pollutants and IL-8 in the two-pollutant model

| **Exposure window** | **Ambient air pollutants** | | | | | |
| --- | --- | --- | --- | --- | --- | --- |
| **(No. of observations)** | **PM_10_** | **PM_2.5_** | **SO_2_** | **NO_2_** | **CO** | **O_3_** |
| **Serum IL-8 (n=2199)** |  |  |  |  |  |  |
|  | **With SO_2_** | **With SO_2_** | **With PM_10_** | **With PM_10_** | **With PM_10_** | **With PM_10_** |
| 1-day average | 1.06 (–0.50, 2.64) | **3.20 (0.004, 6.49)** | –0.73 (–1.81, 0.36) | –0.12 (–0.47, 0.24) | 0.08 (–2.42, 2.65) | **1.02 (0.55, 1.49)** |
| 2-day average | **1.86 (0.14, 3.61)** | **5.30 (1.64, 9.09)** | –1.20 (–2.40, 0.01) | –0.13 (–0.53, 0.28) | 0.54 (–2.33, 3.50) | **1.14 (0.63, 1.65)** |
| 3-day average | **2.09 (0.25, 3.98)** | **5.46 (1.52, 9.55)** | **–1.33 (–2.60, –0.04)** | –0.08 (–0.53, 0.38) | 0.82 (–2.40, 4.15) | **1.22 (0.68, 1.77)** |
| 5-day average | **2.56 (0.37, 4.79)** | **7.03 (2.62, 11.63)** | –1.26 (–2.62, 0.13) | 0.09 (–0.44, 0.62) | 2.10 (–1.65, 5.99) | **1.07 (0.50, 1.65)** |
| 7-day average | **3.61 (1.09, 6.19)** | **8.58 (3.74, 13.64)** | **–1.43 (–2.84, –0.001)** | –0.04 (–0.61, 0.53) | 1.36 (–2.73, 5.63) | **1.20 (0.60, 1.80)** |
|  | **With NO_2_** | **With NO_2_** | **With PM_2.5_** | **With PM_2.5_** | **With PM_2.5_** | **With PM_2.5_** |
| 1-day average | 0.87 (–0.78, 2.54) | 2.95 (–0.46, 6.47) | –0.94 (–2.04, 0.17) | –0.20 (–0.56, 0.17) | –0.64 (–3.29, 2.08) | **1.00 (0.53, 1.48)** |
| 2-day average | 1.41 (–0.42, 3.27) | **4.67 (0.66, 8.83)** | **–1.49 (–2.71, –0.25)** | –0.26 (–0.68, 0.17) | –0.47 (–3.58, 2.73) | **1.11 (0.59, 1.62)** |
| 3-day average | 1.43 (–0.54, 3.44) | 4.30 (–0.06, 8.85) | **–1.55 (–2.85, –0.24)** | –0.19 (–0.67, 0.29) | 0.01 (–3.51, 3.65) | **1.19 (0.64, 1.75)** |
| 5-day average | 1.46 (–0.90, 3.88) | **5.44 (0.50, 10.63)** | **–1.52 (–2.88, –0.14)** | –0.10 (–0.64, 0.45) | 0.62 (–3.34, 4.73) | **1.01 (0.43, 1.60)** |
| 7-day average | 2.63 (–0.12, 5.47) | **7.68 (2.17, 13.48)** | **–1.59 (–2.98, –0.18)** | –0.23 (–0.82, 0.36) | –0.23 (–4.51, 4.24) | **1.13 (0.52, 1.74)** |
|  | **With CO** | **With CO** | **With NO_2_** | **With SO_2_** | **With SO_2_** | **With SO_2_** |
| 1-day average | 0.54 (–1.21, 2.32) | 2.47 (–1.27, 6.35) | –0.56 (–1.76, 0.65) | 0.09 (–0.28, 0.46) | 1.16 (–1.06, 3.44) | **1.02 (0.55, 1.50)** |
| 2-day average | 0.88 (–1.05, 2.84) | 3.63 (–0.72, 8.17) | –1.11 (–2.47, 0.26) | 0.25 (–0.17, 0.68) | 2.37 (–0.20, 5.01) | **1.13 (0.62, 1.66)** |
| 3-day average | 0.91 (–1.17, 3.05) | 3.21 (–1.55, 8.20) | –1.40 (–2.84, 0.07) | 0.38 (–0.09, 0.87) | **2.85 (0.02, 5.77)** | **1.22 (0.67, 1.78)** |
| 5-day average | 0.82 (–1.65, 3.36) | 4.33 (–0.96, 9.91) | **–1.65 (–3.20, –0.08)** | **0.63 (0.08, 1.18)** | **3.93 (0.71, 7.24)** | **1.10 (0.52, 1.68)** |
| 7-day average | 1.94 (–0.88, 4.83) | **6.53 (0.75, 12.64)** | –1.59 (–3.20, 0.04) | **0.63 (0.04, 1.22)** | **4.11 (0.54, 7.80)** | **1.26 (0.66, 1.86)** |
|  | **With O_3_** | **With O_3_** | **With CO** | **With O_3_** | **With O_3_** | **With NO_2_** |
| 1-day average | 0.54 (–0.84, 1.93) | 1.29 (–1.44, 4.11) | –0.65 (–1.72, 0.44) | 0.17 (–0.14, 0.47) | 1.13 (–0.87, 3.17) | **1.09 (0.61, 1.58)** |
| 2-day average | 1.00 (–0.53, 2.55) | 2.45 (–0.69, 5.68) | –1.10 (–2.29, 0.11) | 0.23 (–0.12, 0.58) | 1.85 (–0.46, 4.22) | **1.23 (0.71, 1.76)** |
| 3-day average | 1.01 (–0.63, 2.68) | 2.19 (–1.20, 5.69) | –1.22 (–2.48, 0.06) | 0.27 (–0.11, 0.65) | 1.95 (–0.60, 4.56) | **1.31 (0.76, 1.87)** |
| 5-day average | 1.30 (–0.65, 3.29) | 3.52 (–0.40, 7.60) | –1.17 (–2.49, 0.18) | 0.42 (–0.02, 0.86) | 2.93 (–0.03, 5.97) | **1.20 (0.62, 1.78)** |
| 7-day average | 1.87 (–0.38, 4.18) | **4.40 (0.04, 8.94)** | –1.09 (–2.45, 0.29) | 0.43 (–0.04, 0.90) | 3.11 (–0.20, 6.53) | **1.35 (0.75, 1.95)** |
|  |  |  | **With O_3_** |  |  | **With CO** |
| 1-day average | - | - | –0.002 (–0.98, 0.99) | - | - | **1.06 (0.58, 1.53)** |
| 2-day average | - | - | –0.17 (–1.28, 0.94) | - | - | **1.19 (0.67, 1.70)** |
| 3-day average | - | - | –0.24 (–1.41, 0.94) | - | - | **1.26 (0.71, 1.81)** |
| 5-day average | - | - | –0.19 (–1.45, 1.09) | - | - | **1.12 (0.55, 1.69)** |
| 7-day average | - | - | –0.11 (–1.42, 1.21) | - | - | **1.27 (0.67, 1.86)** |

The models are adjusted for date of examination as a linear term, continuous variables of age, body mass index, corresponding moving averages of temperature and relative humidity, and the co-pollutant shown above; and categorical variables of season, weekday of examination, residential region, sex, smoking history, alcohol consumption status, regular exercise, education, occupation, and marital status. Estimates are presented as percentage changes with 95% confidence intervals in each biomarker level per 1-unit increase in 1- to 7-day average ambient air pollution exposure (units for PM: 10 µg/m^3^; SO_2_, NO_2_, and O_3_: 1 ppb; and CO: 0.1 ppm). PM_10_, PM_2.5:_ particulate matter with aerodynamic diameter < 10 µm and < 2.5 µm, respectively; SO_2_: sulfur dioxide; NO_2_: nitrogen dioxide; CO: carbon monoxide; O_3_: ozone; IL: interleukin.

**Supplementary Table 5-4.** Associations between ambient air pollutants and IL-10 in the two-pollutant model

| **Exposure window** | **Ambient air pollutants** | | | | | |
| --- | --- | --- | --- | --- | --- | --- |
| **(No. of observations)** | **PM_10_** | **PM_2.5_** | **SO_2_** | **NO_2_** | **CO** | **O_3_** |
| **Serum IL-10 (n=2199)** |  |  |  |  |  |  |
|  | **With SO_2_** | **With SO_2_** | **With PM_10_** | **With PM_10_** | **With PM_10_** | **With PM_10_** |
| 1-day average | **1.95 (0.49, 3.42)** | **4.17 (1.19, 7.23)** | 0.30 (–0.71, 1.32) | –0.01 (–0.34, 0.31) | 0.14 (–2.17, 2.50) | **–0.44 (–0.87, –0.01)** |
| 2-day average | **2.07 (0.47, 3.69)** | **5.59 (2.20, 9.10)** | 0.44 (–0.69, 1.58) | 0.01 (–0.36, 0.39) | 0.59 (–2.07, 3.32) | **–0.48 (–0.95, –0.02)** |
| 3-day average | 1.60 (–0.10, 3.33) | **3.99 (0.39, 7.72)** | 0.42 (–0.78, 1.64) | 0.06 (–0.36, 0.48) | 1.30 (–1.69, 4.38) | **–0.59 (–1.09, –0.09)** |
| 5-day average | 1.56 (–0.44, 3.61) | 3.68 (–0.28, 7.81) | –0.05 (–1.33, 1.25) | –0.09 (–0.58, 0.40) | 2.03 (–1.44, 5.61) | –0.51 (–1.03, 0.02) |
| 7-day average | 1.40 (–0.89, 3.74) | 2.73 (–1.53, 7.16) | –0.49 (–1.81, 0.85) | –0.22 (–0.75, 0.31) | 1.32 (–2.48, 5.27) | –0.29 (–0.84, 0.26) |
|  | **With NO_2_** | **With NO_2_** | **With PM_2.5_** | **With PM_2.5_** | **With PM_2.5_** | **With PM_2.5_** |
| 1-day average | **2.19 (0.65, 3.75)** | **4.81 (1.60, 8.11)** | 0.22 (–0.81, 1.26) | –0.06 (–0.39, 0.28) | –0.32 (–2.77, 2.19) | **–0.51 (–0.94, –0.08)** |
| 2-day average | **2.31 (0.61, 4.05)** | **6.65 (2.87, 10.56)** | 0.17 (–0.98, 1.33) | –0.14 (–0.53, 0.26) | –0.75 (–3.61, 2.20) | **–0.56 (–1.03, –0.09)** |
| 3-day average | 1.72 (–0.11, 3.58) | **4.58 (0.53, 8.78)** | 0.27 (–0.95, 1.51) | –0.03 (–0.48, 0.41) | 0.57 (–2.70, 3.95) | **–0.66 (–1.16, –0.16)** |
| 5-day average | 1.76 (–0.44, 4.01) | 4.48 (–0.06, 9.23) | –0.14 (–1.42, 1.16) | –0.18 (–0.68, 0.33) | 1.59 (–2.11, 5.43) | **–0.58 (–1.11, –0.04)** |
| 7-day average | 1.63 (–0.90, 4.23) | 3.50 (–1.41, 8.65) | –0.49 (–1.79, 0.84) | –0.26 (–0.80, 0.29) | 1.21 (–2.82, 5.41) | –0.34 (–0.89, 0.22) |
|  | **With CO** | **With CO** | **With NO_2_** | **With SO_2_** | **With SO_2_** | **With SO_2_** |
| 1-day average | **2.09 (0.45, 3.76)** | **4.80 (1.26, 8.46)** | 0.73 (–0.40, 1.86) | 0.11 (–0.23, 0.45) | 1.42 (–0.65, 3.53) | –0.37 (–0.80, 0.07) |
| 2-day average | **2.10 (0.29, 3.93)** | **6.57 (2.44, 10.87)** | 0.83 (–0.44, 2.13) | 0.13 (–0.26, 0.53) | 1.87 (–0.50, 4.30) | –0.38 (–0.86, 0.09) |
| 3-day average | 1.34 (–0.60, 3.32) | 3.83 (–0.61, 8.46) | 0.64 (–0.73, 2.02) | 0.15 (–0.29, 0.60) | 2.13 (–0.48, 4.80) | –0.49 (–1.00, 0.01) |
| 5-day average | 0.69 (–1.60, 3.03) | 2.06 (–2.75, 7.10) | 0.24 (–1.22, 1.72) | 0.08 (–0.43, 0.59) | 2.72 (–0.22, 5.75) | –0.44 (–0.97, 0.09) |
| 7-day average | 0.48 (–2.09, 3.12) | 1.03 (–4.05, 6.39) | –0.17 (–1.68, 1.36) | 0.02 (–0.53, 0.57) | 2.14 (–1.10, 5.49) | –0.27 (–0.82, 0.28) |
|  | **With O_3_** | **With O_3_** | **With CO** | **With O_3_** | **With O_3_** | **With NO_2_** |
| 1-day average | **2.17 (0.88, 3.47)** | **4.78 (2.16, 7.47)** | 0.62 (–0.38, 1.64) | 0.18 (–0.11, 0.46) | 1.79 (–0.07, 3.69) | –0.36 (–0.81, 0.09) |
| 2-day average | **2.39 (0.95, 3.84)** | **6.21 (3.20, 9.31)** | 0.71 (–0.42, 1.84) | 0.23 (–0.09, 0.55) | **2.35 (0.19, 4.55)** | –0.38 (–0.86, 0.10) |
| 3-day average | **1.98 (0.45, 3.54)** | **4.96 (1.74, 8.29)** | 0.52 (–0.67, 1.72) | 0.22 (–0.14, 0.57) | **2.51 (0.13, 4.94)** | –0.50 (–1.00, 0.01) |
| 5-day average | 1.72 (–0.10, 3.57) | **4.29 (0.62, 8.09)** | –0.04 (–1.29, 1.21) | 0.08 (–0.33, 0.49) | 2.67 (–0.07, 5.48) | –0.44 (–0.97, 0.09) |
| 7-day average | 1.18 (–0.90, 3.31) | 2.63 (–1.36, 6.78) | –0.43 (–1.70, 0.85) | –0.05 (–0.49, 0.39) | 1.76 (–1.28, 4.89) | –0.26 (–0.81, 0.29) |
|  |  |  | **With O_3_** |  |  | **With CO** |
| 1-day average | - | - | 0.80 (–0.12, 1.72) | - | - | –0.38 (–0.82, 0.05) |
| 2-day average | - | - | 0.94 (–0.09, 1.98) | - | - | –0.41 (–0.88, 0.06) |
| 3-day average | - | - | 0.74 (–0.35, 1.85) | - | - | –0.53 (–1.03, –0.03) |
| 5-day average | - | - | 0.23 (–0.94, 1.42) | - | - | –0.45 (–0.97, 0.07) |
| 7-day average | - | - | –0.23 (–1.44, 0.99) | - | - | –0.25 (–0.79, 0.29) |

The models are adjusted for date of examination as a linear term, continuous variables of age, body mass index, corresponding moving averages of temperature and relative humidity, and the co-pollutant shown above; and categorical variables of season, weekday of examination, residential region, sex, smoking history, alcohol consumption status, regular exercise, education, occupation, and marital status. Estimates are presented as percentage changes with 95% confidence intervals in each biomarker level per 1-unit increase in 1- to 7-day average ambient air pollution exposure (units for PM: 10 µg/m^3^; SO_2_, NO_2_, and O_3_: 1 ppb; and CO: 0.1 ppm). PM_10_, PM_2.5:_ particulate matter with aerodynamic diameter < 10 µm and < 2.5 µm, respectively; SO_2_: sulfur dioxide; NO_2_: nitrogen dioxide; CO: carbon monoxide; O_3_: ozone; IL: interleukin.

**Supplementary Table 5-5.** Associations between ambient air pollutants and TNF-α in the two-pollutant model

| **Exposure window** | **Ambient air pollutants** | | | | | |
| --- | --- | --- | --- | --- | --- | --- |
| **(No. of observations)** | **PM_10_** | **PM_2.5_** | **SO_2_** | **NO_2_** | **CO** | **O_3_** |
| **Serum TNF-α (n=2199)** |  |  |  |  |  |  |
|  | **With SO_2_** | **With SO_2_** | **With PM_10_** | **With PM_10_** | **With PM_10_** | **With PM_10_** |
| 1-day average | 0.49 (–0.20, 1.19) | 1.48 (0.07, 2.91) | 0.04 (–0.45, 0.53) | 0.001 (–0.16, 0.16) | 0.19 (–0.93, 1.32) | –0.14 (–0.34, 0.07) |
| 2-day average | 0.65 (–0.11, 1.42) | 1.88 (0.29, 3.50) | 0.05 (–0.49, 0.60) | 0.01 (–0.17, 0.19) | 0.41 (–0.88, 1.71) | –0.10 (–0.33, 0.12) |
| 3-day average | 0.39 (–0.42, 1.21) | 1.20 (–0.50, 2.93) | 0.20 (–0.38, 0.78) | 0.06 (–0.14, 0.26) | 0.71 (–0.74, 2.17) | –0.09 (–0.33, 0.15) |
| 5-day average | 0.14 (–0.82, 1.10) | 0.76 (–1.11, 2.67) | 0.24 (–0.38, 0.87) | 0.16 (–0.07, 0.40) | 1.60 (–0.07, 3.31) | –0.19 (–0.45, 0.06) |
| 7-day average | 0.14 (–0.96, 1.24) | 0.78 (–1.25, 2.85) | 0.22 (–0.42, 0.87) | 0.18 (–0.08, 0.43) | **2.05 (0.20, 3.95)** | –0.17 (–0.44, 0.09) |
|  | **With NO_2_** | **With NO_2_** | **With PM_2.5_** | **With PM_2.5_** | **With PM_2.5_** | **With PM_2.5_** |
| 1-day average | 0.52 (–0.21, 1.26) | **1.62 (0.11, 3.15)** | –0.06 (–0.55, 0.44) | –0.04 (–0.20, 0.12) | –0.19 (–1.38, 1.01) | –0.16 (–0.37, 0.05) |
| 2-day average | 0.67 (–0.14, 1.49) | **2.08 (0.33, 3.87)** | –0.05 (–0.61, 0.50) | –0.05 (–0.24, 0.14) | –0.05 (–1.45, 1.37) | –0.13 (–0.35, 0.10) |
| 3-day average | 0.38 (–0.50, 1.26) | 1.28 (–0.63, 3.22) | 0.12 (–0.47, 0.72) | 0.02 (–0.20, 0.23) | 0.40 (–1.19, 2.01) | –0.11 (–0.36, 0.13) |
| 5-day average | –0.12 (–1.16, 0.93) | 0.30 (–1.82, 2.47) | 0.17 (–0.45, 0.80) | 0.13 (–0.12, 0.37) | 1.39 (–0.40, 3.21) | –0.22 (–0.48, 0.04) |
| 7-day average | –0.18 (–1.38, 1.04) | 0.24 (–2.08, 2.61) | 0.16 (–0.48, 0.80) | 0.14 (–0.12, 0.40) | 1.88 (–0.09, 3.88) | –0.20 (–0.47, 0.07) |
|  | **With CO** | **With CO** | **With NO_2_** | **With SO_2_** | **With SO_2_** | **With SO_2_** |
| 1-day average | 0.44 (–0.34, 1.23) | 1.58 (–0.08, 3.28) | 0.13 (–0.41, 0.67) | 0.04 (–0.13, 0.20) | 0.50 (–0.50, 1.50) | –0.12 (–0.33, 0.09) |
| 2-day average | 0.52 (–0.34, 1.39) | 1.85 (–0.07, 3.82) | 0.14 (–0.47, 0.76) | 0.06 (–0.13, 0.25) | 0.80 (–0.34, 1.95) | –0.08 (–0.31, 0.15) |
| 3-day average | 0.23 (–0.70, 1.17) | 1.00 (–1.10, 3.14) | 0.20 (–0.46, 0.86) | 0.07 (–0.15, 0.28) | 0.77 (–0.47, 2.04) | –0.06 (–0.30, 0.19) |
| 5-day average | –0.36 (–1.46, 0.75) | –0.24 (–2.53, 2.10) | 0.03 (–0.68, 0.74) | 0.14 (–0.10, 0.39) | 1.18 (–0.23, 2.60) | –0.17 (–0.42, 0.09) |
| 7-day average | –0.54 (–1.77, 0.71) | –0.59 (–3.02, 1.91) | –0.01 (–0.73, 0.73) | 0.16 (–0.11, 0.42) | 1.52 (–0.05, 3.10) | –0.15 (–0.41, 0.12) |
|  | **With O_3_** | **With O_3_** | **With CO** | **With O_3_** | **With O_3_** | **With NO_2_** |
| 1-day average | 0.53 (–0.09, 1.14) | **1.49 (0.26, 2.74)** | 0.09 (–0.39, 0.58) | 0.04 (–0.09, 0.18) | 0.52 (–0.37, 1.42) | –0.12 (–0.33, 0.10) |
| 2-day average | **0.70 (0.02, 1.39)** | **1.89 (0.49, 3.32)** | 0.10 (–0.44, 0.64) | 0.08 (–0.08, 0.23) | 0.86 (–0.17, 1.90) | –0.07 (–0.30, 0.16) |
| 3-day average | 0.53 (–0.20, 1.27) | 1.47 (–0.05, 3.01) | 0.17 (–0.40, 0.75) | 0.10 (–0.07, 0.27) | 0.92 (–0.22, 2.07) | –0.06 (–0.30, 0.19) |
| 5-day average | 0.37 (–0.50, 1.25) | 1.28 (–0.45, 3.04) | 0.10 (–0.51, 0.70) | 0.13 (–0.06, 0.33) | 1.25 (–0.05, 2.58) | –0.15 (–0.41, 0.10) |
| 7-day average | 0.40 (–0.60, 1.40) | 1.33 (–0.58, 3.28) | 0.05 (–0.56, 0.67) | 0.14 (–0.07, 0.35) | **1.57 (0.10, 3.05)** | –0.13 (–0.40, 0.13) |
|  |  |  | **With O_3_** |  |  | **With CO** |
| 1-day average | - | - | 0.16 (–0.28, 0.60) | - | - | –0.12 (–0.33, 0.09) |
| 2-day average | - | - | 0.23 (–0.26, 0.73) | - | - | –0.08 (–0.30, 0.15) |
| 3-day average | - | - | 0.30 (–0.23, 0.83) | - | - | –0.07 (–0.31, 0.17) |
| 5-day average | - | - | 0.22 (–0.34, 0.79) | - | - | –0.18 (–0.43, 0.07) |
| 7-day average | - | - | 0.21 (–0.38, 0.80) | - | - | –0.16 (–0.42, 0.10) |

The models are adjusted for date of examination as a linear term, continuous variables of age, body mass index, corresponding moving averages of temperature and relative humidity, and the co-pollutant shown above; and categorical variables of season, weekday of examination, residential region, sex, smoking history, alcohol consumption status, regular exercise, education, occupation, and marital status. Estimates are presented as percentage changes with 95% confidence intervals in each biomarker level per 1-unit increase in 1- to 7-day average ambient air pollution exposure (units for PM: 10 µg/m^3^; SO_2_, NO_2_, and O_3_: 1 ppb; and CO: 0.1 ppm). PM_10_, PM_2.5:_ particulate matter with aerodynamic diameter < 10 µm and < 2.5 µm, respectively; SO_2_: sulfur dioxide; NO_2_: nitrogen dioxide; CO: carbon monoxide; O_3_: ozone; TNF: tumor necrosis factor.

**Supplementary Table 5-6.** Associations between ambient air pollutants and urinary 8-OHdG in the two-pollutant model

| **Exposure window** | **Ambient air pollutants** | | | | | |
| --- | --- | --- | --- | --- | --- | --- |
| **(No. of observations)** | **PM_10_** | **PM_2.5_** | **SO_2_** | **NO_2_** | **CO** | **O_3_** |
| **Urinary 8-OHdG (n=2138)** |  |  |  |  |  |  |
|  | **With SO_2_** | **With SO_2_** | **With PM_10_** | **With PM_10_** | **With PM_10_** | **With PM_10_** |
| 1-day average | 1.41 (–0.18, 3.03) | 2.70 (–0.48, 5.99) | –0.78 (–1.87, 0.32) | 0.06 (–0.29, 0.42) | –1.37 (–3.85, 1.18) | 0.34 (–0.13, 0.82) |
| 2-day average | 1.56 (–0.19, 3.33) | 2.85 (–0.74, 6.56) | –0.96 (–2.18, 0.27) | –0.13 (–0.54, 0.28) | **–2.93 (–5.74, –0.04)** | **0.53 (0.02, 1.05)** |
| 3-day average | **1.93 (0.07, 3.82)** | **3.98 (0.07, 8.03)** | –0.82 (–2.11, 0.50) | –0.04 (–0.50, 0.41) | –3.08 (–6.20, 0.14) | **0.59 (0.04, 1.14)** |
| 5-day average | **2.35 (0.16, 4.60)** | **5.35 (0.98, 9.91)** | –0.89 (–2.27, 0.52) | 0.11 (–0.42, 0.64) | –3.11 (–6.69, 0.61) | 0.51 (–0.06, 1.09) |
| 7-day average | **2.88 (0.34, 5.48)** | **6.01 (1.24, 11.01)** | –0.79 (–2.22, 0.67) | 0.14 (–0.43, 0.72) | –3.78 (–7.71, 0.31) | 0.46 (–0.14, 1.07) |
|  | **With NO_2_** | **With NO_2_** | **With PM_2.5_** | **With PM_2.5_** | **With PM_2.5_** | **With PM_2.5_** |
| 1-day average | 0.72 (–0.95, 2.42) | 1.20 (–2.16, 4.68) | –0.79 (–1.89, 0.33) | 0.07 (–0.29, 0.44) | –1.54 (–4.18, 1.17) | 0.32 (–0.15, 0.80) |
| 2-day average | 1.27 (–0.59, 3.16) | 2.24 (–1.69, 6.32) | –0.95 (–2.19, 0.31) | –0.12 (–0.55, 0.31) | **–3.30 (–6.34, –0.17)** | **0.52 (0.005, 1.04)** |
| 3-day average | 1.52 (–0.47, 3.55) | 3.16 (–1.16, 7.67) | –0.86 (–2.18, 0.48) | –0.07 (–0.55, 0.41) | **–3.94 (–7.34, –0.42)** | **0.57 (0.01, 1.12)** |
| 5-day average | 1.48 (–0.90, 3.91) | 3.84 (–1.04, 8.97) | –0.99 (–2.37, 0.42) | 0.03 (–0.52, 0.58) | **–4.36 (–8.14, –0.42)** | 0.46 (–0.12, 1.05) |
| 7-day average | 1.88 (–0.89, 4.72) | 4.50 (–0.88, 10.17) | –0.82 (–2.23, 0.62) | 0.07 (–0.52, 0.66) | **–5.08 (–9.18, –0.79)** | 0.40 (–0.21, 1.02) |
|  | **With CO** | **With CO** | **With NO_2_** | **With SO_2_** | **With SO_2_** | **With SO_2_** |
| 1-day average | 1.49 (–0.31, 3.33) | 3.07 (–0.71, 7.00) | –0.95 (–2.15, 0.26) | 0.32 (–0.05, 0.69) | 0.30 (–1.91, 2.57) | 0.33 (–0.15, 0.81) |
| 2-day average | **2.19 (0.20, 4.22)** | 4.75 (0.34, 9.34) | –0.82 (–2.19, 0.57) | 0.18 (–0.25, 0.61) | –0.69 (–3.21, 1.89) | 0.52 (–0.004, 1.04) |
| 3-day average | **2.69 (0.55, 4.88)** | 6.66 (1.72, 11.83) | –0.79 (–2.26, 0.71) | 0.30 (–0.18, 0.79) | –0.45 (–3.21, 2.39) | **0.62 (0.06, 1.17)** |
| 5-day average | **3.09 (0.54, 5.70)** | 8.21 (2.68, 14.03) | –1.20 (–2.77, 0.40) | 0.55 (–0.01, 1.10) | –0.11 (–3.22, 3.10) | 0.56 (–0.02, 1.14) |
| 7-day average | **3.93 (1.02, 6.93)** | 9.63 (3.62, 15.98) | –1.11 (–2.73, 0.55) | **0.61 (0.01, 1.21)** | –0.38 (–3.82, 3.18) | 0.54 (–0.06, 1.15) |
|  | **With O_3_** | **With O_3_** | **With CO** | **With O_3_** | **With O_3_** | **With NO_2_** |
| 1-day average | 0.87 (–0.53, 2.29) | 1.41 (–1.35, 4.25) | –0.39 (–1.48, 0.70) | 0.22 (–0.09, 0.53) | 0.13 (–1.86, 2.16) | 0.44 (–0.05, 0.93) |
| 2-day average | 0.89 (–0.66, 2.47) | 1.20 (–1.92, 4.43) | –0.33 (–1.55, 0.90) | 0.11 (–0.24, 0.46) | –0.77 (–3.05, 1.56) | **0.58 (0.05, 1.11)** |
| 3-day average | 1.29 (–0.37, 2.98) | 2.26 (–1.15, 5.80) | –0.13 (–1.42, 1.17) | 0.23 (–0.16, 0.62) | –0.45 (–2.97, 2.13) | **0.68 (0.12, 1.23)** |
| 5-day average | 1.56 (–0.42, 3.58) | 3.37 (–0.59, 7.48) | –0.23 (–1.58, 1.14) | 0.37 (–0.08, 0.81) | –0.29 (–3.18, 2.69) | **0.64 (0.06, 1.22)** |
| 7-day average | 2.02 (–0.27, 4.37) | 4.19 (–0.22, 8.79) | –0.03 (–1.41, 1.38) | 0.44 (–0.04, 0.92) | –0.41 (–3.65, 2.93) | **0.62 (0.02, 1.22)** |
|  |  |  | **With O_3_** |  |  | **With CO** |
| 1-day average | - | - | –0.20 (–1.19, 0.80) | - | - | 0.35 (–0.12, 0.83) |
| 2-day average | - | - | –0.27 (–1.39, 0.86) | - | - | **0.52 (0.01, 1.04)** |
| 3-day average | - | - | 0.01 (–1.18, 1.21) | - | - | **0.61 (0.07, 1.16)** |
| 5-day average | - | - | –0.06 (–1.34, 1.23) | - | - | 0.56 (–0.01, 1.14) |
| 7-day average | - | - | 0.11 (–1.22, 1.44) | - | - | 0.54 (–0.06, 1.14) |

The models are adjusted for date of examination as a linear term, continuous variables of age, body mass index, corresponding moving averages of temperature and relative humidity, and the co-pollutant shown above; and categorical variables of season, weekday of examination, residential region, sex, smoking history, alcohol consumption status, regular exercise, education, occupation, and marital status. Estimates are presented as percentage changes with 95% confidence intervals in each biomarker level per 1-unit increase in 1- to 7-day average ambient air pollution exposure (units for PM: 10 µg/m^3^; SO_2_, NO_2_, and O_3_: 1 ppb; and CO: 0.1 ppm). PM_10_, PM_2.5:_ particulate matter with aerodynamic diameter < 10 µm and < 2.5 µm, respectively; SO_2_: sulfur dioxide; NO_2_: nitrogen dioxide; CO: carbon monoxide; O_3_: ozone; 8-OHdG: 8-hydroxy-2′-deoxyguanosine.
